# First Report of CRISPR/Cas9 Gene Editing in *Castanea sativa* Mill

**DOI:** 10.3389/fpls.2021.728516

**Published:** 2021-08-25

**Authors:** Vera Pavese, Andrea Moglia, Elena Corredoira, Mª Teresa Martínez, Daniela Torello Marinoni, Roberto Botta

**Affiliations:** ^1^Dipartimento di Scienze Agrarie, Forestali e Alimentari (DISAFA), Università degli Studi di Torino, Grugliasco, Italy; ^2^Instituto de Investigaciones Agrobiológicas de Galicia (IIAG)-Consejo Superior de Investigaciones Científicas, Santiago de Compostela, Spain

**Keywords:** European chestnut, *Agrobacterium*-mediated transformation, phytoene desaturase, somatic embryos, targeted mutagenesis, gene knockout

## Abstract

CRISPR/Cas9 has emerged as the most important tool for genome engineering due to its simplicity, design flexibility, and high efficiency. This technology makes it possible to induce point mutations in one or some target sequences simultaneously, as well as to introduce new genetic variants by homology-directed recombination. However, this approach remains largely unexplored in forest species. In this study, we reported the first example of CRISPR/Cas9-mediated gene editing in *Castanea* genus. As a proof of concept, we targeted the gene encoding *phytoene desaturase* (*pds*), whose mutation disrupts chlorophyll biosynthesis allowing for the visual assessment of knockout efficiency. Globular and early torpedo-stage somatic embryos of *Castanea sativa* (European chestnut) were cocultured for 5 days with a CRISPR/Cas9 construct targeting two conserved gene regions of *pds* and subsequently cultured on a selection medium with kanamycin. After 8 weeks of subculture on selection medium, four kanamycin-resistant embryogenetic lines were isolated. Genotyping of these lines through target Sanger sequencing of amplicons revealed successful gene editing. Cotyledonary somatic embryos were maturated on maltose 3% and cold-stored at 4°C for 2 months. Subsequently, embryos were subjected to the germination process to produce albino plants. This study opens the way to the use of the CRISPR/Cas9 system in European chestnut for biotechnological applications

## Introduction

European chestnut or sweet chestnut (*Castanea sativa* Mill.) is a worldwide widely distributed tree species with an important economic role in Spain and Italy. It is highly appreciated for both timber and fruit production (Conedera et al., 2004, 2016). In addition to its main productive role, European chestnut may also play a key role in wildlife and landscape conservation, rural tourism, recreation, and protection from erosion (Merkle et al., 2020). However, European chestnut populations are seriously threatened by two severe diseases as follows: (1) ink disease mainly caused by the oomycete *Phytophthora cinnamomi* and (2) chestnut blight provoked by the fungus *Cryphonectria parasitica*. During the first third of the twentieth century, the crossings between European chestnut and Asian tolerant species (i.e., *Castanea crenata* and *Castanea mollissima*) were the only valid option to deal with the ink disease. Although the hybrids obtained showed tolerance to *P. cinnamomi*, their nut quality was lower when compared to the European chestnut cultivars. The spread of hypovirulence has reduced the impact of canker blight in Europe, but the disease is still a threat in orchards and young plantings, since extensive breeding of *C. sativa* for resistance to *C. parasitica* has not been carried out yet.

Biotechnological methods, such as genetic transformation, can represent an interesting alternative to the traditional breeding of chestnut and could contribute to overcome the disease issue, a major limiting factor for the expansion of modern orchard planting. A prerequisite for transformation is the availability of an adequate *in vitro* plant regeneration procedure. Somatic embryos are considered the best explant to be used as a target in the transformation systems (Corredoira et al., 2019), due to the higher transformation rate than other regeneration methods and to the reduced number of escapes and chimeric plants. In recent years, several procedures have been reported for the establishment of European chestnut somatic embryos lines from zygotic embryos and from shoot apices and leaves isolated from axillary shoot cultures (Corredoira et al., 2006; Merkle et al., 2020). Using these embryogenic systems, efficient protocols of genetic transformation were set up; chestnuts were transformed introducing both marker genes (Corredoira et al., 2004) and genes coding for pathogenesis-related proteins such as thaumatin-like proteins and chitinases, in order to confer tolerance against ink and blight diseases, respectively (Corredoira et al., 2012, 2016).

Targeted genome editing allows the introduction of precise modifications directly into a commercial cultivar, offering a viable alternative to traditional breeding methods. The 2020 Nobel Prize CRISPR/Cas9 technology (www.nobelprize.org/prizes/chemistry/2020) has shown high efficiency in the knockout, insertion, and correction of genes and has sparked great enthusiasm in the scientific community being expected to play a key role in future efforts to improve crop traits (Santillán Martínez et al., 2020). This technology makes it possible to induce point mutations in one or some target sequences simultaneously, as well as to introduce new genetic variants by the homology-directed recombination or to target and modify the transcription. In addition, CRISPR/Cas9 is inexpensive, simple, and highly flexible allowing the rapid target plant genome editing (Walawage et al., 2019). The development of new genome editing technologies in plant breeding fostered a growing interest for *in vitro* culture and regeneration protocols, which represent a major bottleneck in the application of these techniques in many plant species of agricultural and industrial interest.

So far, reports of genome editing in tree species are still limited (Bewg et al., 2018). Nowadays, gene editing has been only reported in a few fruit tree species such as coffee (Breitler et al., 2018), apple (Nishitani et al., 2016), grape (Wang et al., 2018), cocoa (Fister et al., 2018), and walnut (Walawage et al., 2019), while in forest species, gene editing has been only achieved in poplar (Fan et al., 2015).

*Phytoene desaturase* (*pds*) gene plays a central role in chlorophyll biosynthesis and is considered a popular marker for CRISPR/Cas9 editing since its knockout leads to an albino phenotype (Pan et al., 2016; Qin et al., 2007). For this reason, the *pds* gene is used as an endogenous reporter gene for proof-of-concept gene editing in plants (Odipio et al., 2017; Shan et al., 2018; Bernard et al., 2019; Charrier et al., 2019; Ma et al., 2019; Wilson et al., 2019).

In this study, we reported, for the first time, the establishment of a CRISPR/Cas9-based transformation protocol in *C. sativa* using somatic embryos. Our results demonstrated that genome editing through CRISPR/Cas9 can be efficiently applied for chestnut genome modification.

## Materials and Methods

### Plant Material

The embryogenic line CI-3 initiated from zygotic embryos of *C. sativa* Mill. (Corredoira et al., 2006) was used for gene editing experiments. This embryogenic line was maintained by secondary embryogenesis with sequential subcultures at 5/6 weeks intervals onto a multiplication medium consisting of MS (Murashige and Skoog, 1962) mineral salts (half-strength macronutrients; ½ MS) and vitamins, 3 mM glutamine, 0.1 mg/L benzyladenine (BA), 0.1 mg/L 1-naphthaleneacetic acid, 3% sucrose (w/v), and 0.7% Sigma agar (w/v). The pH was adjusted to 5.6–5.7, and the medium was autoclaved at 115°C for 20 min. Cultures were incubated under a 16-h photoperiod (provided by cool-white fluorescent lamps at a photon flux density of 50–60 μmol m^−2^ s^−1^) and 25°C light/20°C dark temperatures (standard conditions).

### Mining of *pds* in the *C. sativa* Transcriptome and Phylogenetic Analysis

*Castanea sativa pds* (*Cspds*) gene sequence was kindly provided by Dr. Susana Serrazina, from the *C. sativa* transcriptome database (Serrazina et al., 2015). *Cspds* gene was analyzed using PROSITE database (https://prosite.expasy.org/; accessed date: January 1, 2021) to annotate functional domains.

The alignment of the *Cspds* gene sequence together with 44 other plant *pds* coding sequences, available on NCBI database (accessed date: January 1, 2021), was performed by multiple sequence alignment using the ClustalW algorithm (http://www.clustal.org/; accessed date: January 1, 2021). MEGA X software (https://www.megasoftware.net/; accessed date: January 1, 2021) was adopted to generate the phylogenetic tree, by applying the maximum-likelihood algorithm. The individual branch statistical significance was assessed by the bootstrap analysis with 1,000 iterations.

### Vector Design

The two gRNAs were designed on the *Cspds* sequence using CRISPR-P 2.0 (http://crispr.hzau.edu.cn/CRISPR2; accessed date: January 1, 2021) (Supplementary File 1). Putative off-target sites were identified with the CRISPOR software (http://crispor.tefor.net/crispor.py), using the *C. mollissima* genome as a reference. The gRNAs were assembled into a CRISPR/Cas9 vector carrying the h*Cas9* and the *nptII* gene for kanamycin (kan) resistance, using the GoldenBraid (GB) assembly system and following the GB software-directed procedures (https://gbcloning.upv.es/; accessed date: January 1, 2021). CaMV 35S promoter and AtU6-26 RNA Pol III promoter were used to drive the hCas9 and gRNA expression, respectively. The final vector named nptII_Cas9_*pds*_gRNA1_gRNA2 (Supplementary File 2) was transferred into *Agrobacterium tumefaciens* strain EHA105 (Hood et al., 1993) by using the freeze/thaw method (Xu and Li, 2008).

### Somatic Embryo Genetic Transformation

Cultures of EHA105-nptII_Cas9_*pds*_gRNA1_gRNA2 for transformation experiments were prepared according to the study by Corredoira et al. (2015). A single colony was inoculated in 2 ml of Luria-Bertani medium (Sambrook et al., 1989) supplemented with 50 mg/L kan, and the culture was grown overnight at 28°C with shaking (200 rpm) in darkness. Of note, 1 ml of this bacterial suspension was added into 600 ml of LB liquid medium added with 50 mg/L kan and was grown at 28°C at 100 rpm in darkness until the attainment of an optical density_600_ = 0.6. Bacteria were recovered by centrifugation (i.e., 6,500 rpm for 10 min at 10°C), and the pellet was resuspended in 200 ml of MS liquid medium supplemented with 5% sucrose (w/v) (i.e., infection medium).

**Figure 1:**
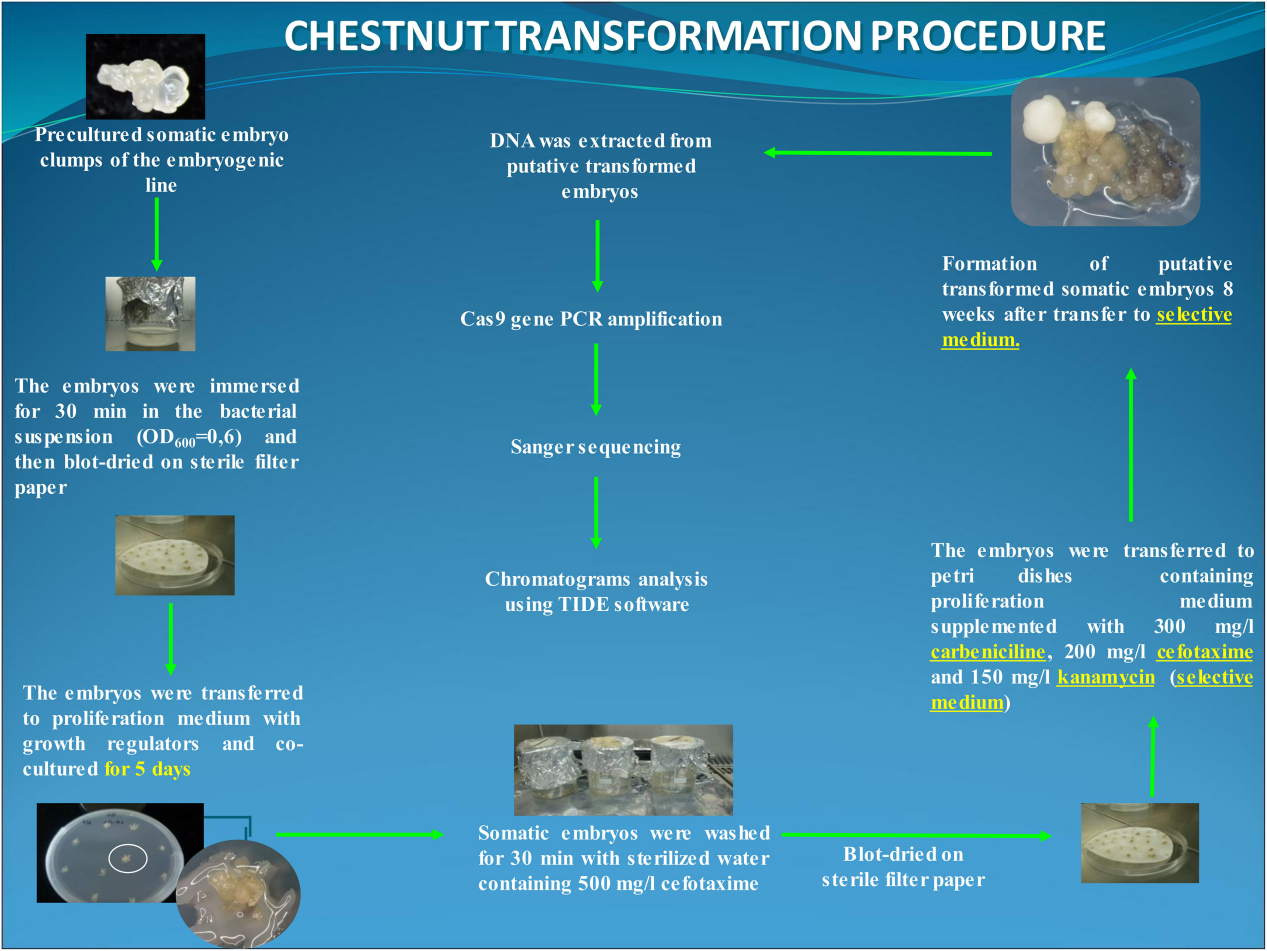
Flowchart of the chestnut somatic embryo transformation protocol using EHA105-nptII_Cas9_*pds*_gRNA1_gRNA2 construct. The putative transformed lines were analyzed at DNA level through the amplification of the *Cas9* gene and Sanger sequencing.

For transformation, clumps of 2–3 somatic embryos at globular or early torpedo stage were employed as target explants. Somatic embryos were precultured on a free plant growth regulator multiplication medium for 1 day before the transformation trial. The explants were immersed for 30 min in the infection medium and transferred to the multiplication medium for cocultivation in dark at 25°C. After 5 days of cocultivation, explants were washed for 30 min with sterilized water with 500 mg/L cefotaxime, and subsequently, somatic embryo groups were transferred to multiplication medium supplemented with carbenicillin (300 mg/L), cefotaxime (200 mg/L), and kanamycin (150 mg/L) (i.e., selection medium) and cultured under standard conditions. In this experiment, 60 explants (6 Petri dishes with 10 explants per dish) were transformed. In addition, 10 clumps of somatic embryos were transformed with the nptII/Cas9 vector (gRNA-free control), and 20 groups of uninfected embryos were cultured to be used as the negative and positive control, respectively (Figure 1).

After 8 weeks on the selection medium, kanamycin-resistant embryos were detected and transferred to a fresh selection medium to establish the different mutated lines. These transformed lines were routinely maintained by secondary embryogenesis with sequential subcultures at 6-week intervals on the selection medium according to the study by Corredoira et al. (2015). The multiplication capacity of two transformed/mutated lines was evaluated by recording the number of somatic embryos produced by explant and compared with those of the control embryogenic line.

### Plant Regeneration

Plant regeneration was performed according to the study by Corredoira et al. (2008). Cotyledonary somatic embryos (>5 mm) were isolated and cultured on a maturation medium consisting of ½ MS medium supplemented with 3% maltose (w/v) and 0.8% Sigma agar (w/v). After 4 weeks of culture on the maturation medium at standard conditions, somatic embryos were transferred to empty Petri dishes and stored at 4°C. After 2 months, somatic embryos were cultured on the germination medium consisting of ½ MS supplemented with 0.1 mg/L BA, 0.1 mg/L indole-3-butyric acid, glutamine (200 mg/L), and 0.7% Sigma agar (w/v). After 8 weeks at standard conditions, the percentage of regenerated plants/shoot development was evaluated.

### Molecular Analysis of Kanamycin-Resistant Embryos

Genomic DNA was extracted from 0.1 g of white kanamycin-resistant embryos (obtained through the transformation with nptII_Cas9_*pds*_gRNA1_gRNA2) and from nptII_Cas9 control using the DNeasy Plant Pro Kit, Qiagen, Hilden, Germany. The transgene integration was investigated through h*Cas9* gene amplification (in three technical replicates) using SYBR Green PCR Master Mix (Applied Biosystems, Forster City, CA, USA) and the following PCR program: 95°C/10 min, followed by 40 cycles of 95°C/15 s and 60°C/1 min cycle. All primer sequences are available in Supplementary File 3.

Mutation frequencies at the target sites were evaluated through PCR amplification using primers designed on gRNA flanking regions (Supplementary File 3). DNA was amplified using KAPA HIFI Taq, Roche, Mannheim, Germany and the following PCR program: 95°C/3 min, followed by 30 cycles of 98°C/20 s, 60°C/20 s, 72°C/45 s, and 72°C/3 min. The PCR products were purified using Nucleic Acid Purification, DNA/RNA Clean Up E.Z.N.A.® kit, Gel Extraction, Omega Bio-tek, Norcross, GA, USA. Samples were sequenced using the Sanger method, and the chromatograms obtained were analyzed using the TIDE online software (http://shinyapps.datacurators.nl/tide/; accessed date: January 1, 2021).

### Statistical Analysis

The influence of mutation on somatic embryo proliferation (Table 1) was evaluated by one-way factorial ANOVA (ANOVA I) applying SPSS software for Windows (version 26.0, SPSS Inc., Chicago, IL, USA).

**Table 1:** Secondary embryogenesis ability of non-transgenic line (CI-3-wt) and two mutated lines (W1 and W2).

**Embryogenic line**	**Embryo stage**		
	**Globular-torpedo n°/explant**	**Cotyledonary n°/explant**	**Total n^**°**^of somatic embryos/explant**
CI-3-wt	7.0 ± 0.9	8.5 ± 0.9	15.5 ± 0.8
W1	6.2 ± 1.2	4.8 ± 0.8	11.0 ± 0.8
W2	7.5 ± 0.7	5.3 ± 0.7	12.8 ± 1.0
**ANOVA I**	ns	*p* ≤ 0.05	*p* ≤ 0.05

*The data show the total number of somatic embryos per explant and the number of embryos per developmental stage, recorded after 6 weeks of culture in selection medium. Values are means ± SE of 6 Petri dishes with 8 explants each. ANOVA I significances are shown for each parameter. ns, not significant*.

## Results and Discussion

### Gene Structure and Phylogenetic Analysis

**Figure 2:**
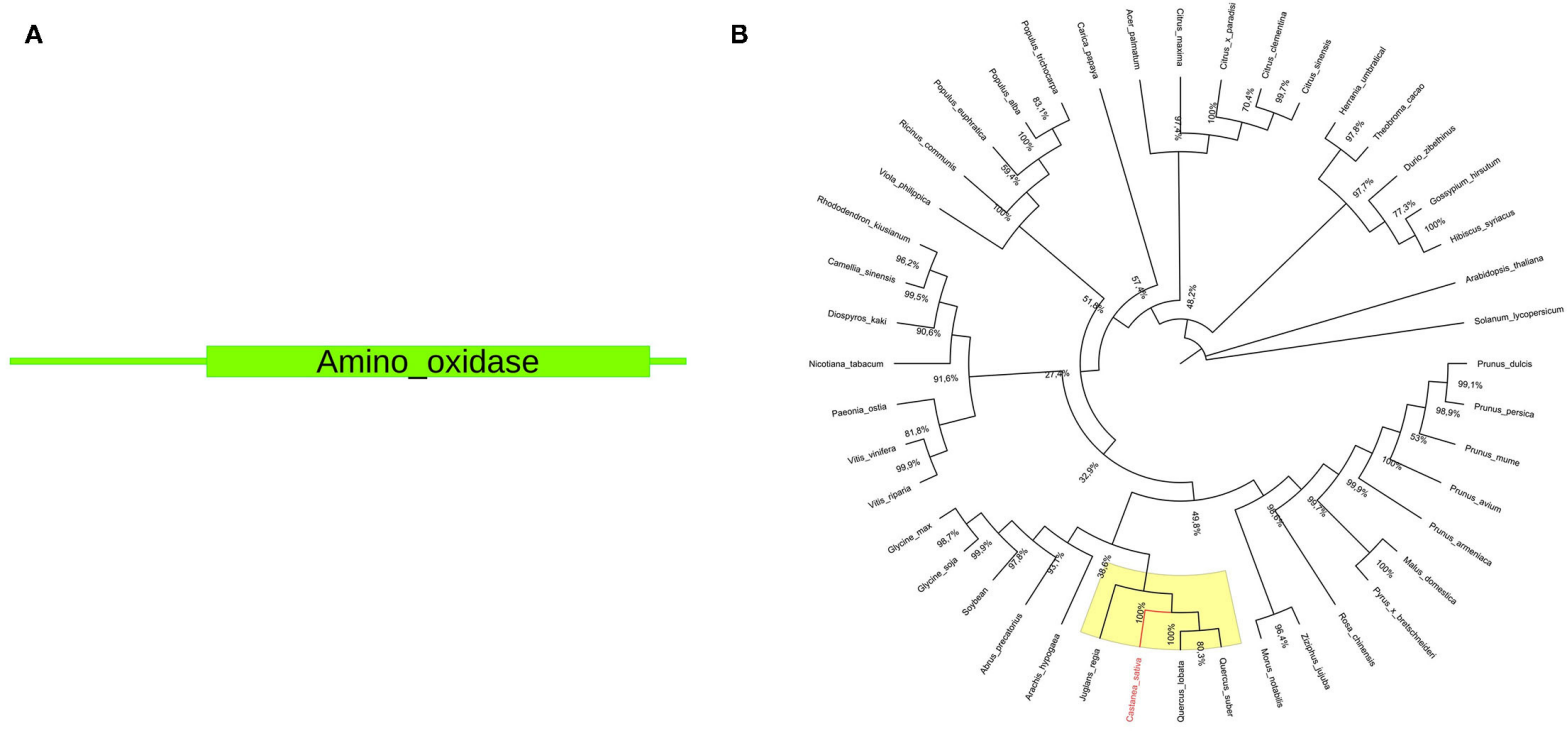
**(A)** Structural domains of *Cspds*. The amino_oxidase domain (PF01593) ranges from 71 to 467 amino acids. **(B)** Phylogenetic analysis of *pds* gene. The phylogenetic tree was constructed using MEGA X software by aligning chestnut *pds* coding sequences with NCBI S-gene ortholog coding sequences. The yellow color indicates the *Castanea sativa* clade. The *C. sativa* sequence is highlighted in red color.

The *pds* gene sequence is 1,498 bp long, and the protein size is 472 amino acids. A single amino_oxidase domain (PF01593) is present within the protein sequence (Figure 2). Available full-length NCBI *pds* ortholog coding sequences (Supplementary File 4) were used for phylogenetic tree construction. The resulting unrooted maximum-likelihood tree is shown in Figure 2.

Underlined in yellow a subclade containing *Cspds* together with gene sequences closely related to *Quercus* spp. and *Juglans regia* (100% bootstrap value) is reported. Both are nut species belonging to the Fagales order (Bernard et al., 2018). Genus *Castanea* and *Quercus* belong to the same Fagaceae family, while genus *Juglans* belongs to Juglandaceae family. This result suggests the common origin of chestnut, oak, and walnut *pds* gene. The phylogenetic relationship of gene sequences from *Quercus, Juglans*, and *Castanea* was also confirmed in the study by Pavese et al. (2021).

### CRISPR/Cas9-Mediated Transformation of Somatic Embryos

The main problems in the application of genome editing technologies in woody species include low transformation efficiency, recalcitrance to transformation, and difficulties in plant regeneration. Moreover, the predominantly outcrossing nature of trees and highly heterozygous genomes represent additional challenges. The limited number of whole-genome sequences available hampers the design of effective sgRNA and the reduction of off-target effects.

To determine whether the CRISPR/Cas9 system may be suitable for gene editing in *C. sativa*, we used the *pds* gene as a target. As this gene is related to chlorophyll biosynthesis (Walawage et al., 2019; Wang et al., 2020), an albino phenotype of somatic embryos is expected, which would allow easier discrimination of mutated embryos. This visual marker has also been applied in defining editing methods in other woody species such as cassava (Odipio et al., 2017), coffee (Breitler et al., 2018), and walnut (Walawage et al., 2019). Besides the proof-of-concept studies, the CRISPR/Cas9 system has been applied to obtain disease-resistant fruit trees (Bewg et al., 2018).

Two unique gRNAs were designed, directed at the coding sequences of *pds* gene. One gRNA targeted the amino_oxidase domain, while the other was chosen to be as close as possible to the 5′ end of the coding sequence, in order to ensure that mutations would affect the protein translation. No loci were found in the *Castanea* genome, which could be considered as a likely source of off-target gene editing. Each gRNA was put under the control of the Arabidopsis U6-26 promoter. The transcription efficiency of sgRNA is pivotal for an efficient CRISPR/Cas9 genome editing. Both endogenous and exogenous and U6 promoters have been successfully deployed for controlling sgRNA transcription in plants. The AtU6 promoter was used in poplar (Fan et al., 2015), apple (Nishitani et al., 2016), and grape (Wang et al., 2018).

The GB cloning system, suited for gene editing experiments, was previously used (Vazquez-Vilar et al., 2016; Maioli et al., 2020). We adopted a dual sgRNA construct to increase the genome editing efficiency either by increasing the possibility that at least one gRNA will be active for mutagenesis or by deleting large gene fragments, in case double-strand breaks are simultaneous (Supplementary File 5) (Xie et al., 2015; Pauwels et al., 2018).

**Figure 3:**
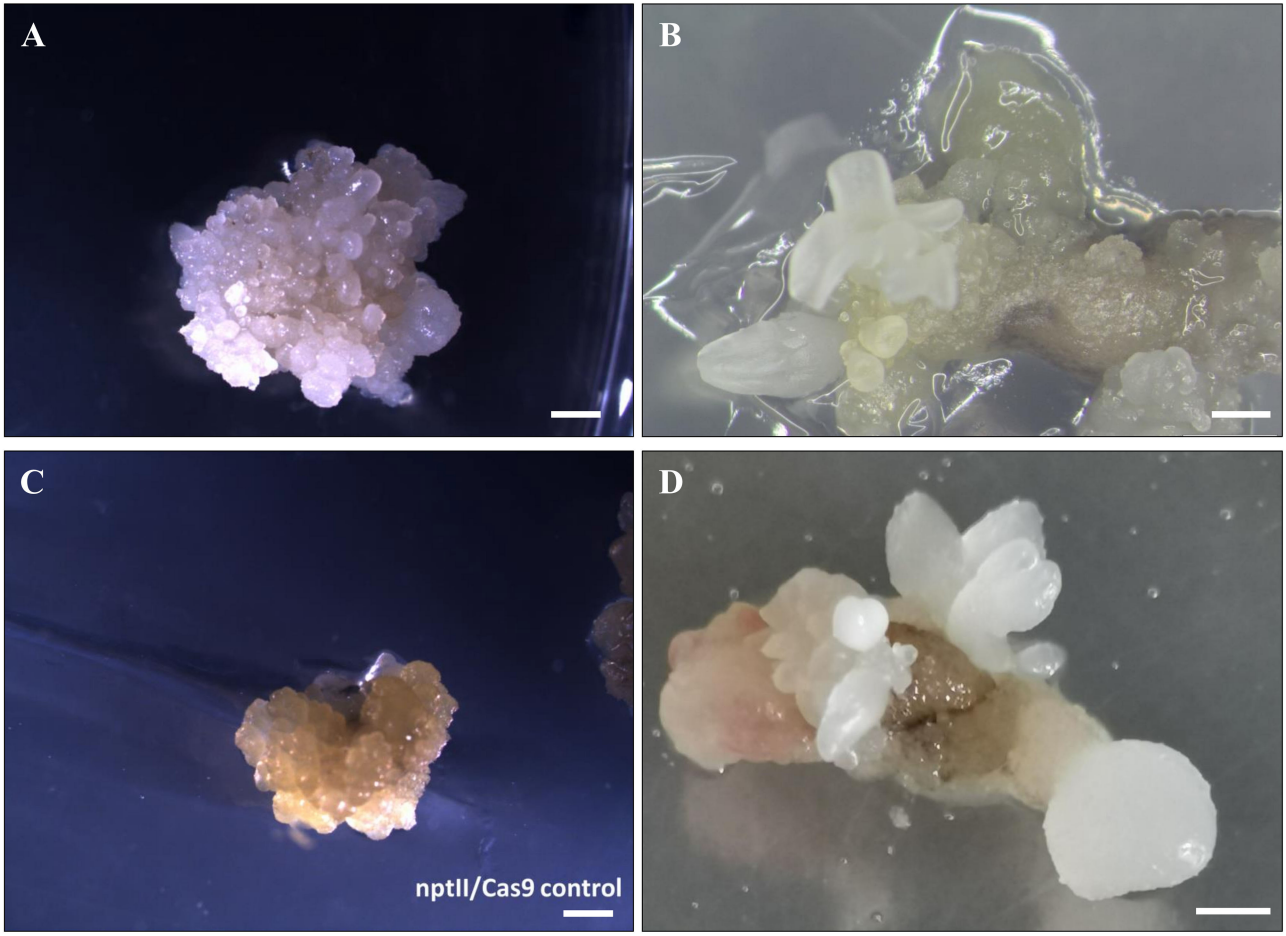
Gene editing of somatic embryos of European chestnut. **(A,B)** White transgenic somatic embryos at different developmental stages formed after 12 weeks on selection medium. **(C)** nptII/Cas9 control. **(D)** Secondary embryos of W1 line generated following 6 weeks of culture on selection medium. Bar: 1 mm.

Somatic embryos at the globular or torpedo stage were transformed with EHA105-nptII_Cas9_*pds*_gRNA1_gRNA2, by applying the previously defined protocol (Corredoira et al., 2012, 2016). Since many hardwood species are recalcitrant to *in vitro* regeneration by organogenesis, somatic embryogenesis (SE) is considered one of the best methods of producing modified plants by genetic engineering (Peña and Séguin, 2001; Corredoira et al., 2019). When somatic embryos are used as target explants, an important factor to achieve a successful genetic transformation is the election of the most suitable embryo developmental stage. It is known that in somatic embryos, at the early developmental stage, many cells are undergoing active cell division, and *Agrobacterium* infection is, therefore, more feasible (Corredoira et al., 2007). In this study, using globular and/or torpedo-stage embryos, 6.6% of kanamycin-resistant explants were obtained after 5 days of cocultivation with *Agrobacterium* and 8 weeks on the selection medium. This rate is similar to those reported in previous studies, in which the same embryogenic lines were transformed with a thaumatin-like gene (Corredoira et al., 2012) or a chitinase gene (Corredoira et al., 2016). In addition, visual evidence of altered *pds* function was given in all kanamycin-resistant explants by producing albino somatic embryos (Figures 3A,B). By contrast, white somatic embryos were not detected in the nptII/Cas9 control (Figure 3C).

### Somatic Embryo Proliferation, Maturation, and Germination Process

One somatic embryo was isolated from each kanamycin-resistant explant and independently cultured to establish four different embryogenetic lines, namely, W1, W2, W3, and W4. These mutated lines were successfully multiplied by secondary embryogenesis on selection medium (Figure 3D) to produce enough material for both maturation and germination steps and for molecular mutation screening. To ascertain whether the multiplication ability is affected by the transformation, the number of secondary somatic embryos of lines W1 and W2 relative to the non-transgenic line was determined after 6 weeks of culture on selective medium (Table 1). The mutated lines produced significantly fewer somatic embryos (11.0–12.8) than the non-transgenic line (15.5). Regarding the number of somatic embryos in relation to the developmental stage, we did not find any differences in the number of globular–torpedo developmental stages; however, the number of cotyledonary embryos was significantly higher in the non-transgenic line (*p* ≤ 0.05), which limited the subsequent maturation step (Table 1).

**Figure 4:**
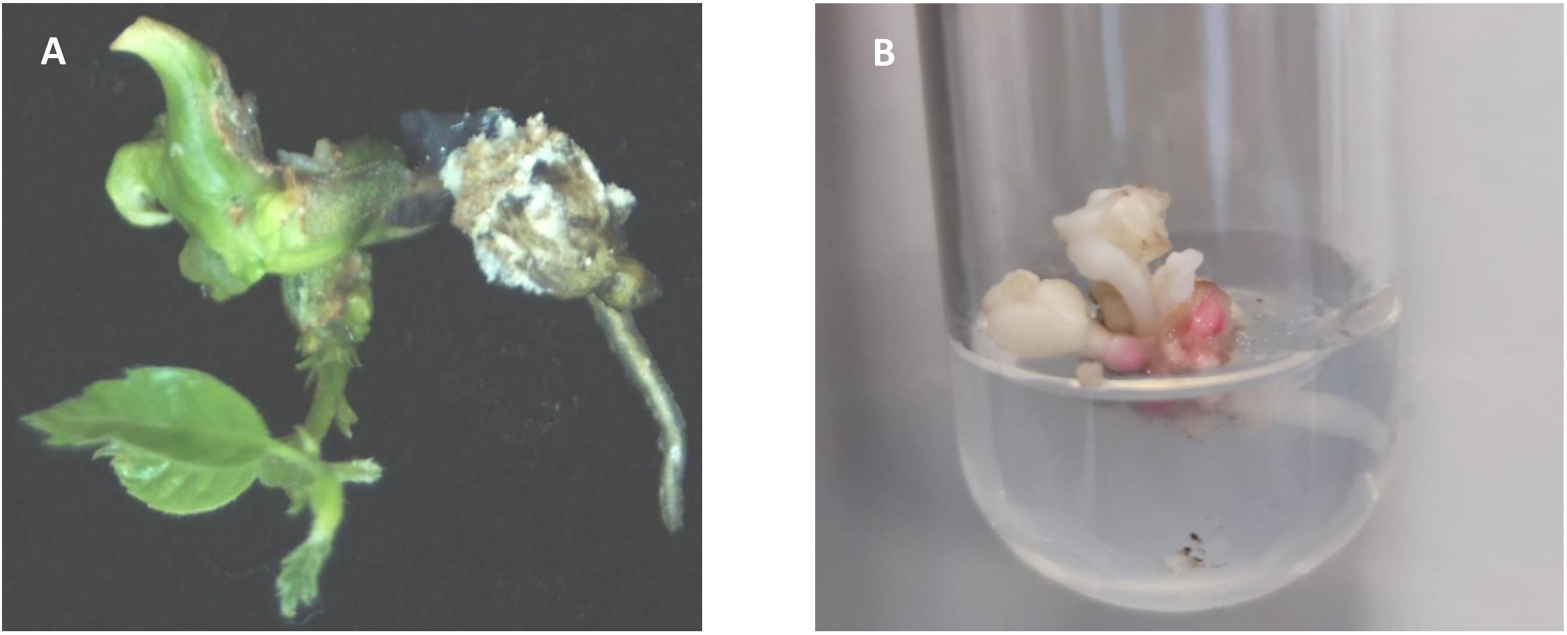
**(A)** Green plant regenerated from an untransformed somatic embryo. **(B)** Albino shoot originated from a germinated somatic embryo of transgenic line W1, showing a stunted morphology with shorter internodes and small leaves.

Mutated cotyledonary embryos were allowed to mature on maltose medium followed by a 2-month cold storage period. Only 50% of embryos of W1 line, 30% of W2 line, 13% of W3 line, and 6% of W4 line survived to cold period. Surviving explants were transferred to the germination medium. Only shoot development was observed (13% of W1 line, 3% of W2 line, 7% of W3, and 2% of W4 line). The low plant regeneration rate of somatic embryos is a common problem in SE systems in hardwoods (Corredoira et al., 2019). This problem has also been reported in European chestnut, especially in transgenic somatic embryos in which simultaneous development of shoot and root is occasionally observed (Corredoira et al., 2004, 2012, 2015). Moreover, it is known that the mutation of *pds* gene negatively affects plant conversion. Breitler et al. (2018) pointed out that the loss of function of *pds* gene causes a reduction in photosynthetic pigments that provoke the low germination rates. Similarly, Walawage et al. (2019) found in walnut that *pds* gene is essential for proper plant growth. As expected, chestnut-transformed somatic embryos showed shoots displaying the typical albino phenotype, but a stunted phenotype with shorter internodes and small leaves was also observed (Figures 4A,B). Similar phenotypic aberrations were described in rice, apple, sweet orange, and poplar (Odipio et al., 2017). In *Arabidopsis*, the alteration of *pds* gene function causes dwarfism and albino phenotypes due to impaired chlorophyll, gibberellin, and carotenoid biosynthesis (Breitler et al., 2018).

### Somatic Embryogenesis Mutation Screening

The qPCR analysis using Cas9 gene-specific primers revealed the genomic integration of the construct in W1–W4 embryo lines (Supplementary File 6). To detect the *pds* gene editing efficiency and the types of mutations, the Sanger sequencing was used in association with TIDE software (Table 2). The average gene editing efficiency was 61% for gRNA1 and 56% for gRNA2. Although the editing efficiency was different between the two gRNAs, it was not possible to attribute this dissimilarity to the different guanine and cytosine content of target sequences, or to the sgRNA secondary structure, or to the promoters that direct Cas9 and gRNAs expression (Ma et al., 2015). This reinforces the importance of assuring efficient knockout by employing different gRNAs.

**Table 2:** Genotyping of targeted gene mutations induced by CRISPR/Cas9 in T_0_ generations in the four edited lines.

**Sample**	**−10**	**−4**	**−3**	**−2**	**−1**	**0**	**1**	**Efficiency**	***R* ^**2**^**	**Zygosity**
W1_gRNA1				2.8	5.4	2.8	85.9	94.3%	0.97	CH
W2_gRNA1						19.1	76.5	77.7%	0.97	HE
W3_gRNA1						61.2	35.3	35.4%	0.97	HE
W4_gRNA1						59.2	36.8	36.8%	0.96	HE
W1_gRNA2				21.8	37.9		18.4	80.4%	0.8	CH
W2_gRNA2	10.9	15.8		8.3	30.7		14.1	79.8%	0.8	CH
W3_gRNA2						54.2	26.8	27.6%	0.82	HE
W4_gRNA2					35.4	48.7		36.1%	0.85	HE

*Editing efficiency, allelic asset (HE, heterozygous; CH, chimeric), and allele frequency are indicated*.

**Figure 5:**
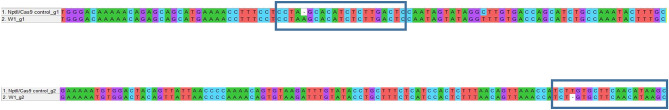
Example of genotyping of targeted gene mutations induced by CRISPR/Cas9 in the W1 line. The blue box underlines the gRNA sequences. When compared with nptII/Cas9 control, the W1 line showed a nucleotide insertion at gRNA1 level and a nucleotide deletion at gRNA2 level.

**Figure 6:**
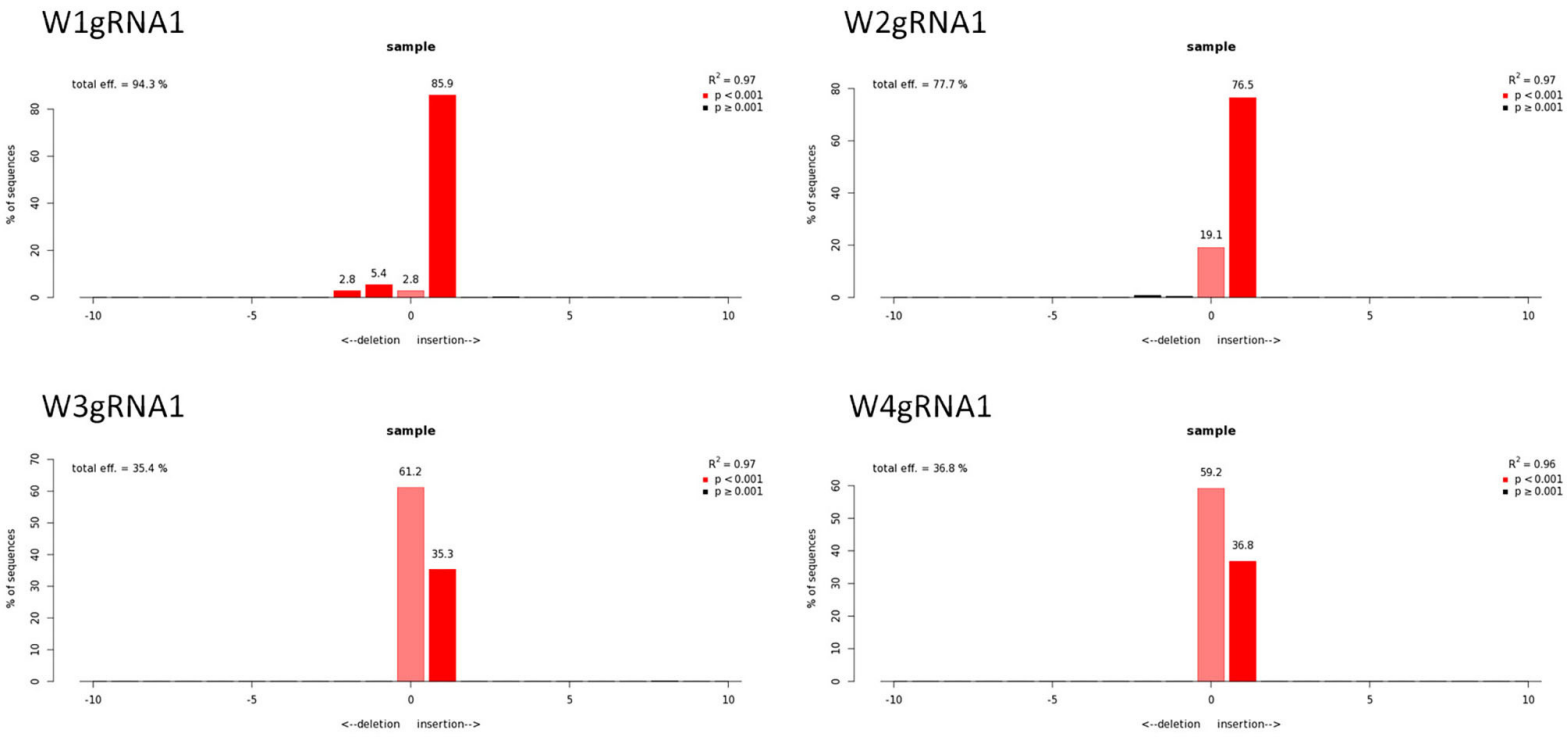
Genotyping of targeted gene mutations induced by CRISPR/Cas9 on gRNA1 in the transformed lines (W1–W4). Editing efficiency and mutagenesis frequencies are reported.

**Figure 7:**
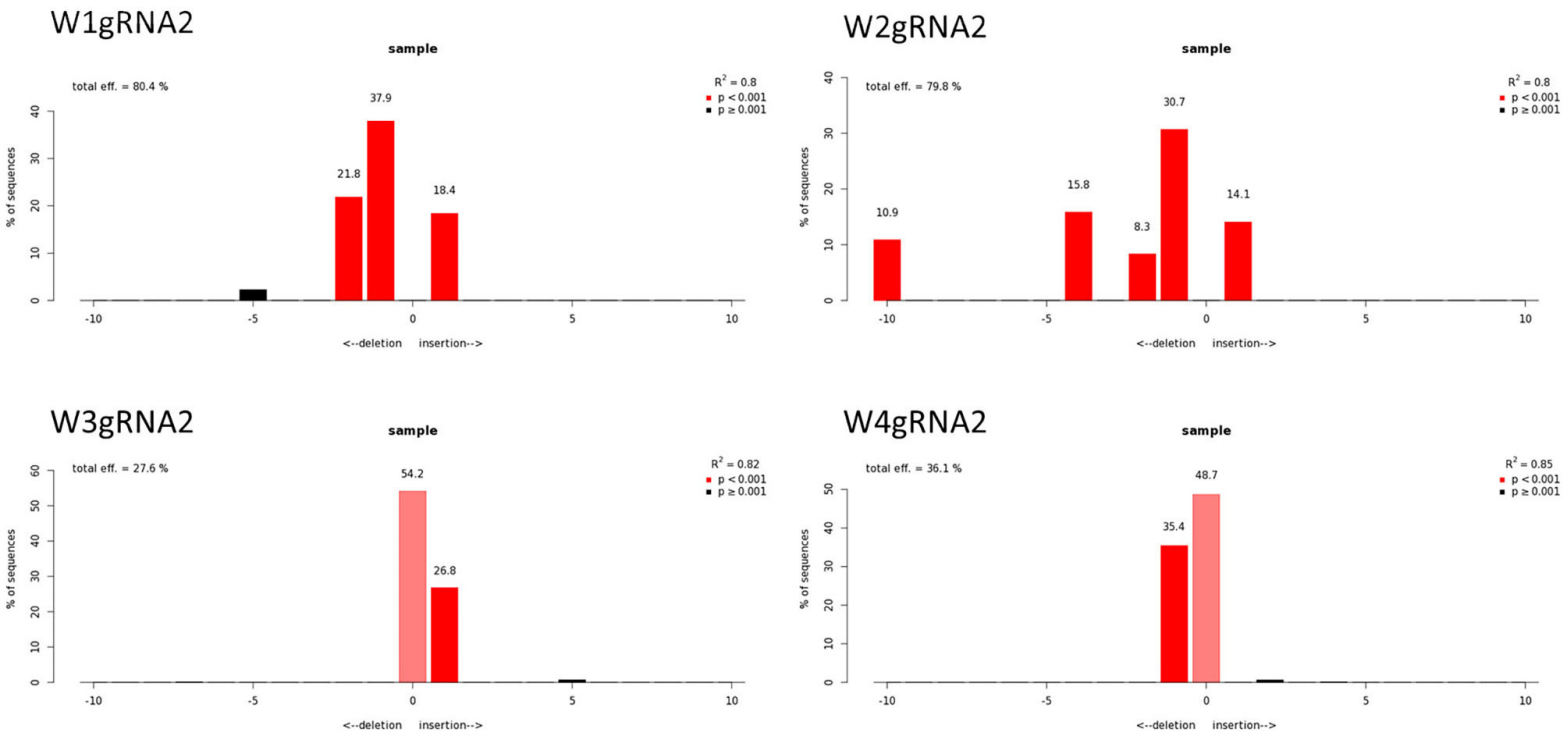
Genotyping of targeted gene mutations induced by CRISPR/Cas9 on gRNA2 in the transformed lines (W1–W4). Editing efficiency and mutagenesis frequencies are reported.

**Figure 8:**
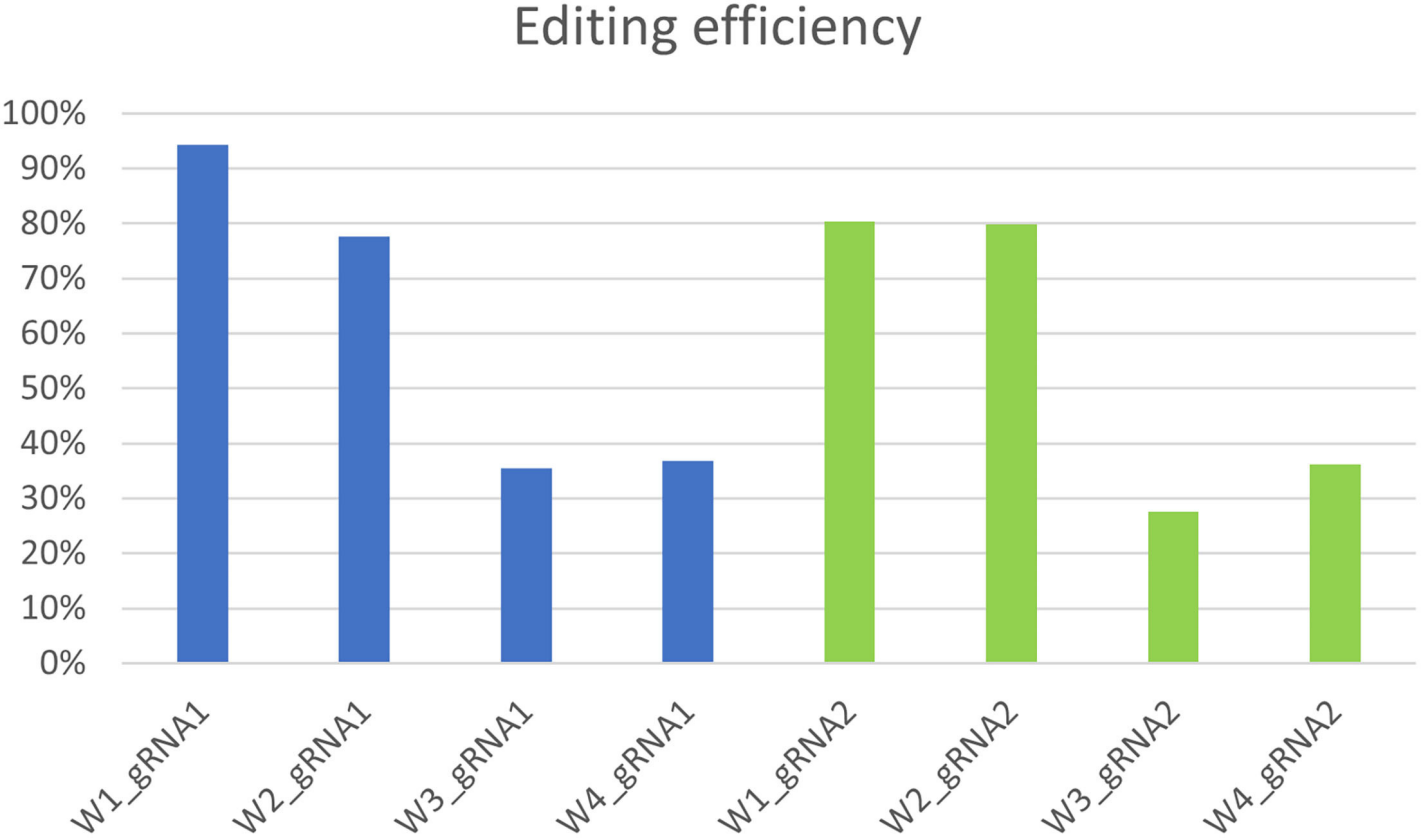
Genotyping of targeted gene mutations induced by CRISPR/Cas9 on gRNA1 and gRNA2 in the transformed lines (W1–W4).

Molecular data demonstrated a higher editing efficiency in W1 and W2 lines than in W3 and W4 lines for both gRNAs tested. W1 showed the greatest editing efficiency, i.e., 94% for gRNA1 and 80% for gRNA2 (Figures 5–8). In the case of gRNA1, three lines were heterozygous and one chimeric; in the case of gRNA2, two lines were chimeric and two heterozygous mutants. Our results are in contrast with what was observed in other woody plants (e.g., grape, pear, apple, or poplar) showing a high level of homologous and biallelic mutants in their T_0_ generation (Dai et al., 2021).

The obtaining of chimeric genotypes by transgenic protocols or genome editing is a very challenging problem (Ding et al., 2020). Self-pollination is usually used in the most model and crop plants to obtain homozygous individuals from heterozygous transgenic or gene edited plants. However, it is difficult to obtain homozygous mutants by this method in woody trees that have long vegetative period and are often self-incompatible. A recent study has reported that the second round of shoot regeneration and a further selection with kanamycin can produce homozygous mutant shoots at a high frequency in poplar (Ding et al., 2020).

The most common mutations in our T_0_ chestnut plantlets were represented by a single nucleotide insertion followed by the deletions of 1, 2, 4, and 10 nucleotides. Previous observations showed that small indels are the predominant mutations introduced in plants by gene editing, with 1 bp insertions, especially +T and +A, predominant in most cases (Bortesi et al., 2016). However, considerable variations in type and size of mutations are reported in the literature (Jacobs et al., 2015; Xu et al., 2015), highlighting a possible influence of target site sequences and/or of their genomic contexts.

## Conclusion

We reported for the first time the application of CRISPR/Cas9 technology in *Castanea* genus. Our system, based on the use of somatic embryos and two guide RNAs directed simultaneously at *pds* locus, demonstrated to be specific for the target gene. Non-pigmented “albino” shoots obtained from *in vitro* cultures were associated with the successful editing of this gene. Since the antibiotics reduced the percentage and efficiency of regeneration, it will be interesting to optimize the transformation protocol trying to minimize the effect of these substances on the regeneration process.

The presence of an effective gene editing method will facilitate the chestnut breeding improvement, acting on genes responsible for pathogen resistance/susceptibility, such as the two candidate S genes (i.e., *pmr4* and *dmr6*) potentially involved in *C. sativa* susceptibility to *C. parasitica* and *P. cinnamomi* (Pavese et al., 2021).

However, a further step of the research may consider the development of genome editing tools that do not require the integration of the CRISPR/Cas9 cassette. An efficient DNA-free genome editing system was developed using *in vitro* assembled Cas9/sgRNA ribonucleoproteins that are delivered in plant protoplasts using polyethylene glycol-mediated transfection (Woo et al., 2015; Malnoy et al., 2016).

## Data Availability Statement

The original contributions presented in the study are included in the article/Supplementary Materials, further inquiries can be directed to the corresponding author.

## Author Contributions

AM, DT, and RB: conceptualization. VP, AM, and EC: data curation and writing—original draft preparation. VP, AM, EC, and TM: investigation. AM, DT, RB, EC, and TM: supervision and writing—review and editing preparation. All authors contributed to the article and approved the submitted version.

## Conflict of Interest

The authors declare that the research was conducted in the absence of any commercial or financial relationships that could be construed as a potential conflict of interest.

## Publisher's Note

All claims expressed in this article are solely those of the authors and do not necessarily represent those of their affiliated organizations, or those of the publisher, the editors and the reviewers. Any product that may be evaluated in this article, or claim that may be made by its manufacturer, is not guaranteed or endorsed by the publisher.

## Supplementary Material

Supplementary File 1 Selected gRNA sequences predicted from *C. sativa pds* (Correspond to Data sheet 1).

Supplementary File 2 CRISPR/Cas9 vector nptII_Cas9_*pds*_gRNA1_gRNA2 assembled using GoldenBraid (GB) cloning system (Correspond to image 1).

Supplementary File 3 Primer sequences (Correspond to Data sheet 2).

Supplementary File 4 *Pds* coding sequences used for tree construction (Correspond to Data sheet 3).

Supplementary File 5 Focus on nptII_Cas9_*pds*_gRNA1_gRNA2 vector at gRNA level (Correspond to image 2).

Supplementary File 6 PCR analysis of Cas9 integration in the different embryogenetic lines (Correspond to image 3).
