# Supplementary material for: First Report of CRISPR/Cas9 Gene Editing in Castanea sativa Mill
Source: Front Plant Sci. 2021 Aug 25;12:728516. doi: 10.3389/fpls.2021.728516 (PMC8424114; doi:10.3389/fpls.2021.728516)
Supplement: Supplementary File 1 — Selected gRNA sequences predicted from C. sativa pds (Correspond to Data sheet 1). [file Data_Sheet_1.zip › Supplemetary File 1.PDF]

gRNA sequence	Score	Sequence	strand	position (bp)	%GC
**gRNA 1**	0.7126	GAGTCAAGAGATGTGCTAGG**AGG**	+	372	50%
**gRNA 2**	0.6328	GCTTATGTTGAAGCACAAGA**TGG**	+	708	40%
