# Supplementary material for: First Report of CRISPR/Cas9 Gene Editing in Castanea sativa Mill
Source: Front Plant Sci. 2021 Aug 25;12:728516. doi: 10.3389/fpls.2021.728516 (PMC8424114; doi:10.3389/fpls.2021.728516)
Supplement: Supplementary File 1 — Selected gRNA sequences predicted from C. sativa pds (Correspond to Data sheet 1). [file Data_Sheet_1.zip › Supplemetary File 3.PDF]

| | |
|---|---|
| gRNA1_F | ATTGAGTCAAGAGATGTGCTAGG |
| gRNA1_R | AAACCCTAGCACATCTCTTGACT |
| gRNA2_F | ATTGCTTATGTTGAAGCACAAGA |
| gRNA2_R | AAACTCTTGTGCTTCAACATAAG |
| RT_Cas9_F | CTATCCTCAGGCGGCAAGAG |
| RT_Cas9_R | AGTCATCCACGCGAATCTGG |
| Seq_*pds*_gRNA1_F | TGGAAACTTTGGGTATGCATCC |
| Seq_*pds*_gRNA1_R | TTCTGTGATTGGTAGGCTTTCA |
| Seq_*pds*_gRNA2_F | AGGTTTGGTCTAAGGAAGCTGA |
| Seq_*pds*_gRNA2_R | TTGTGAGATGACCCCAAATAGT |
| Actin 7_F | CCAAGGCCAACAGGGAAAA |
| Actin7_R | CGGCCTGGATAGCAACATACA |
