# Supplementary material for: First Report of CRISPR/Cas9 Gene Editing in Castanea sativa Mill
Source: Front Plant Sci. 2021 Aug 25;12:728516. doi: 10.3389/fpls.2021.728516 (PMC8424114; doi:10.3389/fpls.2021.728516)
Supplement: Supplementary File 1 — Selected gRNA sequences predicted from C. sativa pds (Correspond to Data sheet 1). [file Data_Sheet_1.zip › Supplemetary File 4.PDF]

#*pds* coding sequences used for tree construction

>*Quercus suber*

ATGACCATTGGTGGGTTTGTTTCGGCTGCAAACTTGAGCTGCCAAAGTACTTTGACAGGAAATCAAACTC

TGGGATGTGGGTTTCTTAATAATTCAGTGAAAACCAATGCATTAGCATTTGGAGGTTGTGAATCCATGGG

TCATAGTTTGAGAATTCCACATACAAAGGCTATTAGATTGAGGCCGAGGAAGGGTGTCTCTCCTTTGCAG

GTAGTATGTATGGACTTCCCAAGACCAGAGCTTGAGAATACTGTTAATTTCTTAGAGGCTGCTTATTTGT

CTTCTTCCTTTCGTGCATCTGCTCGTCCATCTAAACCCCTAACAGTTGTAATTGCTGGTGCAGGTTTGGC

TGGTTTGTCTACTGCAAAGTATTTGGCAGATGCTGGTCACAAACCTATACTATTGGAGTCAAGAGATGTA

CTAGGAGGAAAGGTGGCTGCATGGAAAGATGACGATGGAGACTGGTATGAGACTGGATTACATATATTCT

TTGGGGCTTACCCAAATGTGCAGAATCTGTTCGGAGAACTTGGTATTGATGATCGGTTGCAATGGAAGGA

ACATTCTATGATTTTTGCAATGCCAAATAAGCCGGGAGAGTTCAGCCGATTTGATTTTCCTGAAGTTCTT

CCTGCACCATTAAATGGAATATGGGCTATCTTGAAGAACAATGAGATGCTGACTTGGCCAGATAAAGTCA

AGTTTGCGATTGGACTCTTGCCGGCAATGCTTGGTGGACAGGCTTATGTTGAAGCTCAAGATGGTTTAAC

TGTTAAAGAGTGGATGAGAAAGCAGGGAGTACCTGATCGTGTAACTGATGAGGTATTTGTAGCCATGTCA

AAGGCGCTAAACTTCATTAACCCCGATGAACTTTCAATGCAATGCATATTGATTGCTTTGAATAGGTTTC

TTCAGGAGAAGAATGGTTCCAAGATGGCTTTCTTGGATGGTAATCCCCCAGAGAGACTCTGTATGCCAAT

TGTTGATCATATTCAATCACTAGGTGGTGAAGTAAGACTAAATTCGAGAATACAAAAAATCGAGCTAAAT

AATGATGGAACAGTGAAAAGCTTTTTACTGAATAATGGGAACATGATTGAAGGAGATGCTTATGTATTTG

CTACTCCAGTTGATATCCTGAAGCTTCTTTTGCCGGAAAACTGGAAAGAGATTCCATATTTCCAGAGATT

AAAGAAATTAGTTGGAGTTCCAGTTATTAATGTCCACATATGGTTTGACAGAAAACTGAAGAACACCTAT

GATCACCTACTGTTTAGCAGAAGTCCACTTCTCAGTGTGTATGCTGACATGTCAGTAACATGTAAGGAAT

ATTACAACCCAAACCAATCTATGCTGGAGTTGGTTTTTGCGCCTGCAGAAGAATGGATTTCACGCAGTGA

CTCAGACATTATTGACGCTACAATGAATGAACTTGCAAGACTCTTTCCTGATGAAATTTCCACGGATCAA

AGCAAAGCAAAGATTGTGAAGTACCATGTTGTTAAAACACCAAGGTCTGTTTACAAAACTGTCCCAGACT

GTGAACCTTGCCGTCCCTTACAAAGATCTCCTATTGAGGGGTTTTACTTAGCTGGTGACTACACAAAACA

AAAATATTTGGCTTCAATGGAAGGTGCTGTTCTGTCAGGAAAGCTTTGTGCTCAGGCTATTGTACAGGAT

TATGAGTTGCTTATAGCTCGGGGGCAAACAAGGTTGGCTCAAGCAAGTGTTTATTGA

>*Quercus lobata*

ATGACCATTGGTGGGTTTGTTTCGGCTGCAAACTTGAGCTGCCAAAGTACTTTGACAGGAAATCAAACTC

TGAGATGTGGGTTTCTTAATAATTCAGTGAAAACCAATGCATTAGCATTTGGAGGTTGTGAATCCATGGG

CCATAGTTTGAGAATTCCACATACAAAGGCTATTAGATTGAGGCCGAGGAAGGGTGTCTCTCCTTTGCAG

GTAGTATGTATGGACTTTCCAAGACCAGAGCTTGAGAATACTGTTAATTTCTTAGAGGCTGCTTATTTGT

CTTCTTCCTTTCGTGCATCTGCTCGTCCATCTAAACCCCTAACAGTTGTAATTGCTGGTGCAGGTTTGGC

TGGTTTGTCTACTGCAAAGTATTTGGCAGATGCTGGTCACAAACCTATACTATTGGAGTCAAGAGATGTA

CTAGGAGGAAAGGTGGCTGCATGGAAAGATAACGATGGAGACTGGTATGAGACTGGATTACATATATTCT

TTGGGGCTTACCCAAATGTGCAGAATCTGTTCGGAGAACTTGGTATTGATGATCGGTTGCAATGGAAGGA

GCATTCTATGATTTTTGCAATGCCAAATAAGCCGGGAGAGTTCAGCCGATTTGATTTTCCTGAAGTTCTT

CCTGCACCATTAAATGGAATATGGGCTATCTTGAAGAACAATGAGATGCTGACTTGGCCAGATAAAGTCA

AGTTTGCGATTGGACTCTTGCCAGCAATGCTTGGTGGACAGGCTTATGTTGAAGCTCAAGATGGTTTAAC

TGTTAAAGAGTGGATGAGAAAGCAGGGAGTACCTGATCGTGTAACTGATGAGGTATTTGTAGCCATGTCA

AAGGCGCTAAACTTCATTAACCCCGATGAACTTTCAATGCAATGCATATTGATTGCTTTGAATAGGTTTC

TTCAGGAGAAGAATGGTTCCAAGATGGCTTTCTTGGATGGTAATCCCCCAGAGAGACTCTGTATGCCAAT

TGTTGATCATATTCAATCACTAGGTGGTGAAGTAAGACTAAATTCAAGAATACAAAAAATCGAGCTAAAT

AATGATGGAACAGTGAAAAGCTTTTTACTGAATAATGGGAACATGATTGAAGGAGATGCTTATGTATTTG

CTACTCCGGTTGATATCCTGAAGCTTCTTTTGCCGGAAAACTGGAAAGAGATTCCATATTTCCAGAGATT

AAAGAAATTAGTTGGAGTTCCAGTTATTAATGTCCACATATGGTTTGACAGAAAACTGAAGAACACCTAT

GATCACCTACTGTTTAGCAGAAGTCCACTTCTCAGTGTGTATGCTGACATGTCAGTAACATGTAAGGAAT

ATTACAACCCAAACCAATCTATGCTGGAGTTGGTTTTTGCGCCTGCAGAAGAATGGATTTCACGCAGTGA

CTCAGACATTATTGACGCTACAATGAATGAACTTGCAAGACTCTTTCCCGATGAAATTTCCACGGATCAA

AGCAAAGCAAAGATTGTGAAGTACCATGTTGTTAAAACACCAAGGTCTGTTTACAAAACTGTCCCAGACT

GTGAACCTTGCCGTCCCTTACAAAGATCTCCTATTGAGGGGTTTTACTTAGCTGGTGACTACACAAAACA

AAAATATTTGGCTTCAATGGAAGGTGCTGTTCTGTCAGGAAAGCTTTGTGCTCAGGCTATTGTACAGGAT

TATGAGTTGCTTATAGCTCGGGGGCAAACAAGGTTGGCTCAAGCAAGTGTTTATTGA

>Juglans regia

ATGGCCCTTTGCGGGTGTGTTTCGGCCGCGAACTTGAGCTGGCAGAATACTGTTATAGAAAATCACAGTT

TGAAAACTGCCCCAAGATGTGGGTTTCTTAAAGTTTCAGAGAAAACCAATGCATTAGCATTTGGAGGTAG

TGAATCCATGGGTCATAGTTTGAAAATTAATTATCCACATGCTGTGGGATTAAGGCCAAGGAAGGGTGTC

TACCCTTTGCAGGTAGTATGCATGGACTTCCCAAGACCAGAACTTGATAATACTGTTAATTTCTTAGAAG

CCGCATACCTCTCTTCCTCCTTTCGTACTTCTCCCCGCCCAACTAAACCTTTAACAGTGGTAATTGCTGG

TGCAGGTTTGGCTGGTTTGTCAACCGCAAAGTATTTGGCAGATGCTGGTCATAAACCTGTATTATTGGAA

GCAAGAGATGTTCTTGGAGGAAAGGTGGCTGCATGGAAAGATGACGATGGAGACTGGTATGAGACAGGAT

TACATATATTCTTTGGGGCTTACCCAAATGTGCAGAACATATTTGGAGAACTTGGTATTAATGATCGGTT

GCAGTGGAAGGAGCACTCTATGATATTTGCAATGCCAAAAAAGCCTGGAGAATTCAGCCGATTTGATTTT

CCTGAGGTTCTTCCTGCACCATTAAATGGAATATTTGCTATTTTGAGGAACAATGAAATGTTGAGTTGGC

CAGAGAAAGTCACGTTTGCCATTGGACTTTTACCTGCGATGCTTGGTGGACAGTCCTATGTTGAAGCTCA

AGATGGTTTAACTGTTGAACAGTGGATGAGAAAGCAGGGAATTCCTGATCGTGTAACTGATGAGGTGTTT

ATAGCAATGTCAAAGGCACTAAACTTCATTAACCCTAAGGAACTTTCAATGCAATGTATATTGATTGCTT

TGAACCGGTTTCTTCAGGAAAAGCATGGTTCCAAGATGGCTTTCTTGGATGGTAACCCCCCAGAGAGACT

ATGTCAGCCAATTGTTGATCATATTCAGTCACTGGGTGGTGAAGTAAGACTAAACTCGCGAATTCAGAAT

ATTGAGCTAAATAGTGATGGAACAGTGAAAAGGTTTTTACTGAATAATGGGAACGTGATTGAAGGGGATG

CATATGTATTTGCTACTCCAGTTGACATCCTGAAGCTTCTTTTACCTGAAAACTGGAAAGAGATTCCATA

TTTCCAGAGATTGGAGAAATTAGTTGGAGTTCCAGTTATTAATGTGCACATATGGTTTGACAGGAAACTG

AAGAACACCTATGATCACCTACTATTTAGCAGAAGCCAACTTCTCAGTGTGTATGCTGACATGTCAGTAA

CATGTAAGGAATATTACAACCCAAACGAATCCATGCTGGAATTAGTTTTTGCGCCTGCAGAAGAATGGAT

TTCACGCAGTGATGCTGATATTATTGATGCTACAATGAAGGAACTCGCAAAACTCTTTCCAGATGAAATT

TCCACAGATCAGAGCAAAGCAAAGATTGTAAAGTACCATGTTGTTAAAACACCAAGGTCCGTTTACAAAA

ATGTCCCAAATTGTGAACCCTGCCGTCCCTTACAAAGATCTCCTGTTGAGGGCTTCTACTTAGCTGGTGA

CTACACAAAGCAAAAGTATTTGGCTTCAATGGAAGGTGCTGTTCTGTCAGGAAAGCTTTGTGCTCAAGCT

ATTGTACAGGATTATGAGTTGCTCGTTGCTCGGGGGCAAAGAAGGTTGGCTCAAGCAAGTCTTTTTTGA

>Vitis riparia

ATGACTCAATTCAGATATGTTTCTGCGGTGAACTTGAGCTGCCAAAGTAATATAATAAACTTTCAGAACT

CCCAATGTACCTGGAGACATCTTTATATTGATTCAGATCAGACCAATACACTTCTATTTAGAGGTGGTGA

CTCTATGGGTCTCAAGTTGAGAATTCCAAATAAGCATTCTATTGGAACAAGGCGGAGGAAGGATTTCTGC

CCCTTGCAGGTTGTTTGCATGGATTATCCAAGACCAGAACTTGAGAATACTGTGAATTTCTTAGAAGCTG

CATACTTATCCTCATCCTTTCATACTTCTCCTCGTCCCAGTAAACCATTAGAGGTTGTAATTGCTGGTGC

AGGTTTGGCTGGTTTGTCTACTGCAAAATATTTGGCAGATGCAGGTCACAAGCCTATATTGTTGGAAGCA

AGAGATGTTTTAGGTGGAAAGGTGGCTGCATGGAAAGATGAGGATGGAGACTGGTATGAGACAGGCCTAC

ATATATTCTTTGGGGCTTACCCAAATGTGCAGAACCTGTTTGGAGAACTTGGTATTAATGATCGGTTGCA

GTGGAAGGAACATTCTATGATATTTGCAATGCCAAGCAAGCCAGGGGAATTCAGCCGATTTGATTTCCCT

GAAGTCCTTCCTGCACCCTTAAATGGGATATGGGCCATCTTGAGGAATAATGAAATGCTGACTTGGCCGG

AGAAAATCAAGTTTGCTATTGGACTTGTGCCAGCAATGCTCGGAGGACAGGCTTATGTTGAAGCACAGGA

TGGTTTAACTGTTAAAGACTGGATGAGAAAACAAGGTATTCCTGATCGAGTAACAGATGAGGTTTTCATT

GCCATGTCCAAGGCACTGAACTTCATAAATCCGGATGAACTTTCGATGCAGTGTATATTGATTGCTTTGA

ACCGATTTCTTCAGGAGAAGAATGGCTCCAAGATGGCTTTCTTAGATGGTAATCCTCCAGAGAGACTCTG

CATGCCCATTGTTGACCATATTCAGTCACTAGGTGGTCAAGTCCAACTTAATTCACGAATACAAAAGATT

GAGCTGAACAAAGATGGAACTGTGAAGAGTTTTGTGCTAAATAATGGGAATGTAATTAAAGGAGATGCTT

ATGTAATTGCAACTCCAGTTGATATCCTGAAGCTTCTTTTGCCGGGAGACTGGAAAGAGATTCCATACTT

CAGGAGATTGGATAAATTAGTTGGAGTTCCAGTGATCAATGTTCATATATGGTTTGACAGGAAACTGAAG

AACACATACGATCATCTTCTTTTCAGCAGAAGTCCCCTTCTGAGTGTGTATGCTGACATGTCCGTAACAT

GTAAGGAATATTACAACCCAAATCAATCTATGCTGGAGTTGGTTTTTGCACCTGCTGAAGAATGGGTCTC

ACGCAGTGACTCAGAAATCATTGAAGCTACAATGAAGGAACTTGCCAAACTCTTTCCTGATGAAATTTCA

GAAGATCAGAGCAAAGCGAAAGTTTTGAAATACCATGTTGTTAAAACACCAAGATCTGTTTACAAAACTG

TCCCAAATTGTGAACCTTGCCGTCCCTTACAAAGATCTCCTATAGAGGGCTTTTATTTAGCTGGGGACTA

CACAAAACAAAAATACTTAGCTTCAATGGAAGGTGCTGTTCTGTCAGGGAAGCTTTGTGCACAGGCTATT

GTAAAGGACTATGAATTGCTTGTAGCTCAGGGAGAACAAAAGTTGGCCGAGGTCAGCATTCTCAGTTAA

>Vitis vinifera

ATGACTCAATTCAGATATGTTTCTGTGGTGAACTTGAGCTGCCAAAGTAATATAATAAACTTTCAGAACT

CCCAATGTACCTGGAGACATCTTTATATTGATTCAGATCAGACCAATACACTTCTATTTGGAGGTGGTGA

CTCTATGGGTCTCAAGTTGAGAATTCCAAATAAGCATTCTATTGGAACAAGGCGGAGGAAGGATTTCTGC

CCCTTGCAGGTTGTTTGCATGGATTATCCAAGACCAGAACTTGAGAATACTGTGAATTTCTTAGAAGCTG

CATACTTATCCTCATCCTTTCATACTTCTCCTCGTCCCAGTAAACCATTAGAGGTTGTAATTGCTGGTGC

AGGTTTGGCTGGTTTGTCTACTGCAAAATATTTGGCAGATGCAGGTCACAAGCCTATATTGTTGGAAGCA

AGAGATGTTTTAGGTGGAAAGGTGGCTGCATGGAAAGATGAGGATGGAGACTGGTATGAGACAGGCCTAC

ATATATTCTTTGGGGCTTACCCAAATGTGCAGAACCTGTTTGGAGAACTTGGTATTAATGATCGGTTGCA

GTGGAAGGAACATTCTATGATATTTGCAATGCCAAGCAAGCCAGGGGAATTCAGCCGATTTGATTTCCCT

GAAGTCCTTCCTGCACCCTTAAATGGGATATGGGCCATCTTGAGGAATAATGAAATGCTGACTTGGCCGG

AGAAAATCAAGTTTGCTATTGGACTTGTGCCAGCAATGCTCGGAGGACAGGCTTATGTTGAAGCACAGGA

TGGTTTAACTGTTAAAGACTGGATGAGAAAACAAGGTATTCCTGATCGAGTAACAGATGAGGTTTTCATT

GCCATGTCCAAGGCACTGAACTTCATAAATCCGGATGAACTTTCGATGCAGTGTATATTGATTGCTTTGA

ACCGATTTCTTCAGGAGAAGAATGGCTCCAAGATGGCTTTCTTAGATGGTAATCCTCCAGAGAGACTCTG

CATGCCCATTGTTGACCATATTCAGTCACTAGGTGGTCAAGTCCAACTTAATTCACGAATACAAAAGATT

GAGCTGAACAAAGACGGAACTGTGAAGAGTTTTGTGCTAAATAATGGGAATGTAATTAAAGGAGATGCTT

ATGTAATTGCAACTCCAGTTGATATCCTGAAGCTTCTTTTGCCGGGAGACTGGAAAGAGATTCCATACTT

CAGGAGATTGGATAAATTAGTTGGAGTTCCAGTGATCAATGTTCATATATGGTTTGACAGGAAACTGAAG

AACACATACGATCATCTTCTTTTCAGCAGAAGTCCCCTTCTGAGTGTGTATGCTGACATGTCCGTAACAT

GTAAGGAATATTACAACCCAAATCAATCTATGCTGGAGTTGGTTTTTGCACCTGCTGAAGAATGGGTCTC

ACGCAGTGACTCAGAAATCATTGAAGCTACAATGAAGGAACTTGCCAAACTCTTTCCTGATGAAATTTCA

GAAGATCAGAGCAAAGCGAAAGTTTTGAAATACCATGTTGTTAAAACACCAAGATCTGTTTACAAAACTG

TCCCAAATTGTGAACCTTGCCGTTCCTTACAAAGATCTCCTATAGAAGGGCTTTTATTAGCTGGGGACTA

CACAAAACAAAAATACTTAGCTTCAATGGAAGGTGCTGTTCTGTCAGGGAAGCTTTGTGCACAGGCTATT

GTAAAGGACTATGAATTGCTTGTTGCTCAGGGAGAACAAAAGTTGGCCGAGGTCAGCATTCTCAGTTAA

>Populus alba

ATGAGTGCATTGAACTTGAGCTGGCATAGTAAATCATTAGACTCTCAAGTTGCCTTGAGATGTGGCGCTT

ATCCTACTTGTTCTCACCAAACGAATGCACTAGCTTTTAGAGGCAGTGAATCAATGGGCCATTCTTTGAA

ATTCCCATTTGGAAATTCTTCTGCTAAAACAAGACTAAGGAATCATATCCGCCCTCCTTTGCGGGTTGTC

TGTATGGACTATCCAAGACCGGACCTTGATAACACGGTGAATTTCTTAGAGGCTGCCTTGTTATCTTCAT

CCTTTCGTTCTTCTCCGCGTCCAGCTAAACCATTAAATGTTGTCATTGCTGGTGCAGGTTTGGCGGGTTT

ATCGACTGCAAAATACTTGGCAGATGCGGGCCATAAGCCTATATTGCTTGAAGCAAGAGATGTTTTAGGT

GGAAAGGTGGCTGCATGGAAAGATGATGATGGAGACTGGTACGAGACAGGCTTGCATATATTCTTTGGGG

CATATCCAAATGTGCAGAATCTTTTTGGTGAACTTGGTATCAATGATAGGTTGCAATGGAAGGAGCATTC

TATGATATTTGCAATGCCAAATAAGCCAGGAGAATTCAGTCGATTTGATTTTCCTGAAGTTCTCCCTGCA

CCATTAAATGGGATATTGGCCATTTTAAAGAACAATGAAATGCTGACTTGGCCAGAGAAAGTGAAGTTTG

CAATTGGGCTACTTCCAGCAATTGTTGGTGGACAGGCTTATGTTGAGGCTCAAGATGGTTTAAGTGTTCA

AGAGTGGATGAGAAAGCAGGGTGTACCTGATAGAGTGACTACTGAGGTGTTTATTGCCATGTCAAAGGCT

CTAAACTTTATTAACCCAGATGAGCTTTCAATGCAATGCATTTTGATAGCTTTGAACAGATTTCTTCAGG

AGAAACATGGTTCAAAGATGGCTTTCTTGGATGGTAATCCCCCAGAGAGGCTCTGCATGCCAATTGTTGA

TCATATTCAGTCGCGTGGTGGTGAAGTCAAGCTTAATTCTCGGATAAAGAAGATTGAGCTAAATGATGAT

GGAACAGTGAAGAGTTTTTTACTAAATACTGGGGATGTGATTGAAGGGGATGTTTATGTGTTTGCCACTC

CAGTTGATATCCTGAAGCTTCTTTTGCCTGATAACTGGAAAGAGATTCCTTACTTCAAGAAACTGGAGAA

ATTAGTTGGAGTTCCTGTTATTAATGTTCACATATGGTTTGACAGGAAACTGAAGAATACATACGATCAC

CTACTTTTCAGCAGGAGTCCTCTTCTCAGTGTGTATGCTGACATGTCTCTGACATGTAAGGAATATTATG

ACCCAAATAAATCCATGCTGGAGTTAGTTTTTGCGCCTGCTGAAGAATGGATTTCACGCAGTGACTCAGA

GATTATTGATGCTACAATGGGGGAACTCGCAAAACTTTTTCCTGATGAAATATCCGCAGATCAAAGCAAA

GCAAAAATCGTGAAGTATCATGTTGTTAAAACTCCAAGGTCGGTTTACAAGACTGTCCCAGATTGTGAAC

CTTGCCGTCCCTTGCAAAGATCTCCGATAGAGGGTTTCTATTTAGCTGGTGACTACACAAAACAAAAGTA

CTTGGCTTCAATGGAAGGTGCTGTTCTATCAGGGAAGCTTTGTGCACAGGCAATTATACAGGATTACGAG

TTCCTGGTTGCTCGGGGGCAAGGAAGCTTGACTGAGGCAACCATTAGTTAA

>Populus trichocarpa

ATGAGTGCATTGAACTTGAGCTGGCATAGTAAATCATTAGACTCTCAAGTTGCCTTGAGATGTGGCGCTT

ATCCTACTTGTTCTCACCAAACTAATGCACTAGCTTTTAGAGGCAGTGAATCAATGGGCCATTCTTTGAA

ATTCCCATTTGGAAATTCTTCTGCTAAAACAAGACTAAGGAATCATATCCGCCCTCCTTTGCGGGTTGTC

TGTGTGGACTATCCAAGACCGGACCTTGATAACACGGTGAATTTCTTAGAAGCTGCCTTGTTATCTTCAT

CCTTTCGTTCTTCTCCGCGTCCAGCTAAACCATTAAATGTTGTCATTGCTGGTGCAGGTTTGGCGGGTTT

ATCGACTGCAAAATATTTGGCAGATGCAGGCCATAAGCCTATATTGCTTGAAGCAAGAGATGTTTTAGGT

GGAAAGGTGGCTGCATGGAAAGATGACGATGGAGACTGGTACGAGACAGGCTTGCATATATTCTTTGGGG

CATATCCAAATGTGCAGAATCTTTTTGGTGAACTTGGTATCAATGATAGGTTGCAATGGAAGGAGCATTC

TATGATATTTGCAATGCCAAATAAGCCAGGAGAATTCAGTCGATTTGATTTTCCTGAAGTTCTCCCTGCA

CCATTAAATGGGATATTAGCCATTTTAAAGAACAATGAAATGCTGACTTGGCCAGAGAAAGTGAAGTTTG

CAATTGGGCTACTGCCAGCAATTGTTGGTGGACAAGCTTATGTTGAGGCTCAAGATGGTTTAAGTGTTCA

AGAGTGGATGAGAAAGCAGGGTGTACCTGATAGAGTGACTACTGAGGTGTTTATTGCCATGTCAAAGGCT

CTAAACTTTATTAACCCAGATGAGCTTTCAATGCAATGCATTTTGATAGCTTTGAACAGATTTCTTCAGG

AGAAACATGGTTCAAAGATGGCTTTCTTGGATGGTAATCCCCCAGAGAGGCTCTGCATGCCAATTGTTGA

TCATATTCAGTCGCGTGGTGGTGAAGTCAAGCTTAATTCTCGGATAAAGAAAATTGAGCTAAATGATGAC

GGAACAGTGAAGAGTTTTTTACTAAATACCGGGGATGTGATTGAAGGGGATGTTTATGTGTTTGCCACTC

CAGTTGATACCCTGAAGCTTCTTTTGCCTGATAACTGGAAAGAGATTCCTTACTTCAAGAAACTGGAGAA

ATTAGTTGGAGTTCCTGTTATTAATGTTCACATATGGTTTGACAGGAAACTGAAGAATACATACGATCAC

CTACTTTTCAGCAGGAGTCCTCTTCTCAGTGTGTATGCTGACATGTCTCTGACATGTAAGGAGTATTATG

ACCCAAATAAATCTATGCTGGAGTTAGTTTTTGCGCCTGCTGAAGAATGGATTTCACGCAGTGACTCAGA

GATTATTGATGCTACAATGGGGGAACTTGCAAAACTTTTTCCTGATGAAATATCCGCAGATCAAAGCAAA

GCAAAAATCGTGAAGTATCATGTTGTTAAAACTCCAAGGTCGGTTTACAAGACTGTCCCAGATTGTGAAC

CTTGCCGTCCCTTGCAAAGATCTCCGATAGAGGGTTTCTATTTAGCTGGTGACTACACAAAACAAAAGTA

CTTGGCTTCAATGGAAGGTGCTGTTCTATCAGGGAAGCTTTGTGCACAGGCAATTGTACAGGATTATGAG

TTCCTGGTTGCTCGGGGGCAAGGAAGGTTGACTGAGGCAACCATTACTTAA

> Populus euphratica

ATGAGTGCATTGAACTTGAGCTGGTATAGTAAATCATTAGACTCTCAAGTTGCCTTGAGATGTGGCTCTT

ATCCTTCTTGTTCTCACCAAACTAATGCACTAGCTTTTAGAGGCAGTGAATCAATGGGCCATTCTTTGAA

ATTCGCATTTGGAAATTCTTCTGCTAAAACAAGACTAAGGAATCATATCCGCCCTCCTTTGCGGGTTGTC

TGTGTGGACTATCCAAGACCGGACCTTGATAACACGATGAATTTCTTAGAAGCTGCCTTGTTATCTTCAT

CCTTTCATTCTTCTCCGCGTCCAGCTAAACCATTAAAGGTTGTCATTGCTGGTGCAGGTTTGGCGGGTTT

ATCGACTGCAAAATATTTGGCAGATGCAGGCCATAAGCCTATATTGCTTGAAGCAAGAGATGTTTTAGGT

GGAAAGGTGGCTGCATGGAAGGATGATGATGGAGACTGGTACGAGACAGGCTTGCATATATTCTTTGGGG

CGTATCCAAATGTGCAAAATCTTTTTGGTGAACTTGGTATCAATGATAGGTTGCAATGGAAGGAGCATTC

TATGATATTTGCAATGCCAAATAAGCCAGGAGAATTCAGTCGATTTGATTTTCCTGAATTTCTCCCTGCA

CCATTAAATGGGATATTGGCCATTTTAAAGAACAATGAAATGCTGACTTGGCCAGAAAAAGTGAAGTTTG

CAATTGGGCTACTGCCAGCAATAGTTGGTGGACAGGCTTATGTTGAGGCTCAAGATGGTTTAAGTGTTCA

AGAGTGGATGAGAAAACAGGGTGTACCTGATAGAGTGACTACTGAGGTGTTTATTGCCATGTCAAAGGCT

CTAAACTTTATTAACCCAGATGAGCTTTCAATGCAATGCATTTTGATAGCCTTGAACAGATTTCTTCAGG

AGAAACATGGTTCAAAGATGGCTTTCTTGGATGGTAATCCCCCAGAGAGGCTCTGCATGCCAATTGTTGA

TCATATTCAGTCACGTGGTGGTGAAGTCAAGCTTAATTCTCGGATAAAGAAAATTGAACTAAATGATGAT

GGAACAGTGAAGAGCTTTTTACTAAATACTGGGGATGTGATTGAAGGGGATGTTTATGTGTTTGCCACTC

CAGTTGATACCTTGAAGCTTCTTTTGCCTGAGAACTGGAAAGAGATTCCTTACTTCAAGAAACTGGAGAA

ATTAGTTGGAGTTCCTGTTATTAATGTTCACATATGGTTTGACAGGAAACTGAAGAATACAAACGATCAC

CTACTTTTCAGCAGGAGTCCACTTCTCAGTGTGTATGCTGACATGTCTCTGACATGTAAGGAATATTATG

ACCCAAATAAATCTATGCTGGAGTTGGTTTTTGCACCTGCTGAAGAATGGATTTCACGCAGTGACTCAGA

GATTATTGACGCTACAATGGGGGAACTTGCAAAACTTTTTCCTAATGAAATATCGGCAGATCAAAGCAAA

GCAAAAATCGTGAAGTATCATGTTGTTAAAACTCCAAGGTCGGTTTACAAAACTGTCCCAGATTGTGAAC

CTTGCCGTCCCTTGCAAAGATCTCCAATAGAGGGTTTCTATTTAGCTGGTGACTACACAAAACAGAAGTA

CTTGGCTTCAATGGAAGGTGCTGTTCTATCAGGGAAGCTTTGTGCACAGGCAATTGTACAGGATTATGAG

TTCCTGGTTGCTCGGGGGCAAGGAAGGTTGACCGAGGCAACCATTACTTAA

> Paeonia ostia

ATGGCCCTTTATGGTTGTGTTTCCGCGGTGACGCCAACGCCAAGTAATAAAATCTCGCAATCCACCTTGA

CTCGTGGTTTTCGCATGAAAATCAATCCCGCGATGGCATTTGGAGATAGTGCTGCTATGGGTCTCAGCTT

GAGAATTCCAAATACACACGCCATAACTACGAGGCCTAGAAAAGATGTCTTCCCTTTGCAGGTTGTTTGC

GTGGACTATCCAAGACCAGAGCTTGACAATACTGTTAATTTCTTAGAAGCCGCTTACTTATCATCATTCT

TCCGCTCTTCTTCCCGTCCAAATAAACCGTTGGATGTTGTGATTGCCGGTGCAGGTTTGGCTGGTTTATC

AACTGCAAAATATTTAGCAGATGCAGGTCACAGACCTTTATTGTTGGAAGCAAGAGATGTTCTAGGTGGA

AAGGTGGCTGCATGGAAAGATGACGATGGAGACTGGTATGAGACAGGGCTACATATATTCTTTGGGGCTT

ACCCAAATGTGCAGAACCTGTTTGGAGAACTTGGTATTAATGATCGGTTGCAGTGGAAGGAGCATTCTAT

GATATTTGCCATGCCTAACAAGCCAGGAGAATTCAGCCGATTTGATTTCCTTGAAGTACTGCCTGCACCT

TTAAATGGCCTTTGGGCGATCCTGAAGAACAATGAAATGTTGACTTGGCCAGAAAAAGTGAAATTTGCGA

TTGGACTCTTGCCAGCAATTGTTGGCGGTCAGGCTTATGTTGAGGCTCAAGATGGTTTTACTGTTAAAGA

CTGGATGAGAAAACAAGGGATACCTGATCGAGTAACTAATGAGGTGTTTATTGCCATGTCAAAGGCACTA

AACTTCATAAACCCAGATGAACTTTCAATGCAATGTATTTTGATTGCTTTGAACAGATTTCTTCAGGAGA

AGCATGGTTCCAAGATGGCTTTCTTAGATGGCAATCCTCCAGAGAGACTCTGCATGCCAATTGTTGATCA

TATTGAGTCATTGGGGGGTCAGGTCCGTCTTAATTCAAGAATACAAAAGATTGAGTTGAATAAAGATGGA

ACCGTGAAGGGCTTTTTGCTTAATGATGGGAATTTAATTAAAGGAGATGCTTATGTATTCGCCACTCCAG

TTGACATTCTGAAGCTTCTTCTGCCGAAAGAGTGGAAAGAGATTCCAGACTTTAAAAGACTGGAGAAGTT

AGTTGGAGTTCCAGTTATAAATGTTCACATATGGTTTGACAGGAAGTTGAAGAACACATATGACCATTTA

CTTTTCAGCAGAAGTCCCCTTCTGAGTGTGTATGCTGACATGTCTGTAACTTGTAAGGAATATTACAACC

CAAATGAATCTATGCTGGAGTTGGTTTTTGCTCCTGCAGAAGAATGGATCTCACGTAGTGACTCAGAAAT

TATTGATGCTACGATGAAAGAACTTGCAAAACTGTTTCCTGATGAAATTTCTGCGGATCAGAGCAAGGCT

AAGATTTTGAAGTATCATGTTGTTAAAACACCGAGGTCCGTATATAAGACTGTCCCAGATTGTGAACCAT

GTCGTCCCTTACAAAGATCTCCAATAGAAGGATTCTATTTAGCAGGTGACTATACAAAACAGAAGTATTT

GGCTTCTATGGAAGGTGCTGTTCTATCGGGAAAGTTTTGTGCACAGGCTATTGTGCAGGATTATGAATTG

CTTGTTGCTCGGGAGCCGAAAAAATTGGCTGAGGTTCGCACCCTCTAA

> Camellia sinensis

ATGTCTCAATTTGGACAAGTTTCCACCGTCAGTGTGAGTGGGCAAAACAATGGAATAAGTGTTTGGAACC

CAAAATCTACTTGGGGGTGTGGTTGTTCCTTTGGTTCAGGGCCAGCCAAAGCACTATCATTTCGAGGGAG

TGATTCCATGGGTCATAGGTTCAAAATTCCTAATGCATATGCTGTTGGAACCAGACCAAGGAAGGACGTG

TGCCCTTTGAAGGTGGTTTGCATTGACTATCCAAGACCAGACCTTGAGAGTACTGTCAATTTTTTGGAAG

CTGCCTACTTATCTTCAGCCTTTCGTACTTCCCGCCGTCCAGATAAACCATTGAAGGTTGTTATTGCYGG

TGCAGGTTTGGCTGGTTTGTCTACTGCAAAATATTTGGCAGATGCAGGTCACAAACCTGTATTATTGGAA

GCAAGGGATGTTTTAGGTGGAAAGGTGGCTGCGTGGAAAGATGATGATGGAGACTGGTATGAGACTGGCT

TACATATATTTTTTGGGGCTTACCCAAATGTGCAGAACCTGTTTGGAGAACTTGGTATAAATGATCGATT

GCAGTGGAAAGAGCATTCTATGATATTTGCAATGCCAAACAAGCCAGGGGAGTTCAGCCGATTTGACTTT

CCTGAAGTTCTACCTGCACCATTAAATGGGATATGGGCCATATTAAAGAACAATGAAATGCTTACTTGGC

CTGAGAAAATCAAGTTTGCAATTGGACTCATTCCAGCAATTCTAGGTGGACAGGCCTATGTTGAAGCTCA

AGATGGTTTAAGTGTTAAAGACTGGATGAGGAAGCAAGGTATACCAGATCGAGTAACTACTGAGGTGTTT

ATTGCCATGTCAAAAGCGTTAAACTTCATAAACCCTGATGAACTTTCAATGCAGTGTATTTTGATTGCAC

TGAACCGGTTTCTTCAGGAGAAGCATGGTTCGAAGATGGCATTCTTGGATGGTAACCCCCCAGAGAGACT

TTGCCAGCCAATTGTTGATCATATTCAGTCACTGGGTGGTGAAGTCCAACTTAATTCTCGAATTAAAAAG

ATTGAGCTGAATAAAGATGGAACTGTAAAGAGCTTTTTACTAAATAATGGTAATGCTATTGAAGGAGATG

CCTATGTTTTTGCTACTCCAGTTGATATCTTGAAGCTTCTTTTGCCTGAAGACTGGAAAGAGATTCCGTA

CTTCAGAAAATTGGAGATATTAGTTGGAGTTCCTGTTATAAATGTTCACATATGGTTTGACAGGAAGCTA

AGGAATACTTATGATCATCTACTTTTTAGCAGAAGTCCTCTTCTCAGTGTGTATGCTGACATGTCAGTGG

CATGTAAGGAATATTACGACCCAAATCGCTCTATGCTGGAATTGGTTTTTGCACCTGCAGAGGAATGGAT

CTCATGTAGTGATGAGGAAATTATTGATGCTACGATGAAGGAACTGGCAAAACTCTTTCCTGATGAAATT

TCTGCAGATCAGAGCAAAGCAAAAATATTGAAGTACCATGTTGTTAAAACACCAAGGTCTGTTTATAAAA

CTGTCCCGAACTGTGAACCTTGTCGTCCATTGCAAAGATCCCCTGTAGAAGGGTTCTATTTGTCTGGTGA

CTACACAAAGCAAAAATATTTGGCTTCAATGGAAGGTGCTGTTCTTTCAGGAAAGCTTTGTGCACAAGCT

ATTGTACAGGATTATGAGAAGCTTGTTTCCCGGGAGCAGGGAAAGCTGGCCGAGGCAAGTGTCGTGTAA

>Prunus avium

ATGTCTCAGTGGGCTTGTGTCTCTGCTGCTAACTTGAGCTGCCAAGCTAGCATCATCAACACTCAAAAGC

TACGAAACACTCCCAGATGCGATGCCTTTTCATTTAAAGGTAGTGAATTTATGGCTCAAAGCTGTAGATT

TTTAAGCCCACAAGCCATTCATGGAAGGCCGAGGAATGGTGCTTGCCCTTTGAAGGTGGTTTGCGTTGAT

TATCCAAGACCAGACCTTGACAATACTGCTAATTTCTTAGAAGCTGCATATTTCTCTTCCACTTTCCGAG

CCTCTCCTCGTCCAGCTAAGCCGTTGAAGGTCGTGATTGCTGGTGCAGGTTTGGCTGGTCTGGCAACTGC

AAAATATTTGGCTGATGCAGGTCATAAACCTATCTTACTGGAAGCAAGAGATGTTCTAGGCGGAAAGGTG

GCAGCATGGAAAGATAAGGATGGAGACTGGTACGAAACAGGCCTACATATCTTCTTTGGGGCTTATCCGA

ATATTCAGAACCTGTTTGGTGAGCTTGGTATTGATGATAGATTGCAGTGGAAGGAGCATTCTATGATATT

TGCAATGCCAAACAAACCAGGAGAGTTCAGCCGGTTTGATTTCCCTGAAGTTTTACCAGCACCCTTAAAT

GGAATATGGGCCATATTGAAGAACAATGAGATGCTGACTTGGCCAGAGAAAATCAAGTTTGCAATTGGAC

TACTGCCAGCAATTCTTGGTGGGCAGGCTTATGTTGAAGCCCAAGATGGCTTGAGTGTAAAAGATTGGAT

GAGGAAACAGGGCATACCAGATCGAGTGACCACTGAGGTGTTTATTGCCATGTCAAAGGCCCTGAACTTT

ATTAACCCTGATGAACTTTCAATGCAATGCATATTGATTGCTTTGAACCGATTCCTTCAGGAGAAACACG

GTTCCAAGATGGCTTTCTTGGATGGTAGTCCCCCTGAGAGACTCTGTGCACCAATTGTTGATCATATCCA

GTCATTGGGCGGTGAAGTCCGAATTAATTCCAGAATACAGAAAATTGAGCTAAATAACGATGGGACCGTG

AAGAGTTTTGTACTAAATAATGGGAGCATGATTGAAGCAGATGCCTATGTATTCGCCACTCCAGTTGATA

TCCTAAAGCTTCTATTGCCTGATAACTGGAAAGAGATCCCATATTTCAAGAAATTGAAGAAACTAGTTGG

CGTTCCAGTTATCAATGTTCACATATGGTTTGACAGAAAGCTGAAGAACACATATGATCATCTACTTTTT

AGCAGAAGTCCTCTTTTAAGTGTCTATGCTGACATGTCCGTAACATGTAAGGAATATTACAATCCAAACC

AGTCTATGCTGGAGTTGGTTTTTGCACCAGCAGAAGAATGGATTTCATGCAGTGATTCAGAAATTATTGA

TGCTACACTCAAAGAACTTGCAAAACTCTTTCCTGATGAGATAGCTGCAGATCAAAGCAAAGCAAAGATT

TTGAAGTACCATGTTGTGAAAACACCAAGGTCGGTTTACAAAACTGTACCAGATTGCGAACCTTGCCGTC

CCTTGCAAAGATCTCCCCTAGAGGGTTTCTATTTAGCTGGTGATTATACAAAACAAAAGTATTTAGCCTC

AATGGAAGGTGCTGTTCTGTCAGGGAAACTTTGTGCACAAGCAATTGTACAGGATTACGAATTGCTTGTT

GCTCGGGGACAAACAAGGTTGGCTGAGGCAAGCGTTCGGTGA

>Ziziphus jujuba

ATGTCCCAGTGGGGATGTGTTTCCGCGGCTAACTTGAGCTGGCAAACTAGTAGTATCGTAAGTATTAGGA

AGGTTGGAAGCACACCCAGATGTTGTTTCCAAATGGGTTTGCCAAATTTGGAAGCTTTGACTTTTGGAGG

TAGTGAATTTATGTCTCAGAGTTTGAGAATTCCGTGCTCAAGTGCTACTGGTAGAGGGCAGAGGAAAAGG

GGTTTCCCTTTGAAGGTAGTTTGTGTGGACTATCCAAGACCGGAGCTTGAGAATACTGTTAATTTCTTAG

AAGCTGCTTCCTTGTCTGCTTCCTTTCGCTCTTCTCCTCGTCCTGCTAAACCGTTAAAAGTTGTAATTGC

TGGTGCAGGACTGGCTGGTTTATCAACTGCAAAGTATTTGGCAGATGCAGGTCATAAACCTTTATTACTG

GAAGCAAGAGATGTTTTAGGTGGAAAGCTGGCAGCATGGAAAGATGAGGATGGAGACTGGTATGAGACAG

GCCTACATATATTCTTTGGAGCGTATCCAAATGTACAAAACTTGTTTGGAGAGCTTGGTATTGATGACAG

GTTACAGTGGAAAGAGCATTCTATGATATTTGCAATGCCCAACAAGCCAGGAGAGTTCAGTCGATTTGAC

TTCCTTGAAGCTTTGCCATCACCCATAAATGGAATATGGGCCATTTTGAAGAATAATGAAATGCTGTCTT

GGCCAGAGAAAGTAAAGTTTGCTATTGGTCTTCTACCGGCAATGCTTGGTGGACAGGCTTATGTTGAAGC

TCAAGATAACATCTCTGTTAAAGATTGGATGAGAAAACAGGGCATACCTGATCGAGTAACTGAGGAGGTG

TTTATTGCCATGTCAAAGGCATTAAACTTTATTAACCCTGATGAACTTTCAATGCAATGTATATTGATTG

CTTTGAACAGATTTCTTCAGGAGAAGCATGGTTCCAAGATGGCCTTTTTAGATGGTAATCCTCCAGAGAG

ACTCTGTATGCCAATTGTTGATCATATCGAGAAATTGGGTGGTGAACTCCAACTTAATTCACGGATACAA

AAAATTGAGCTAAATAATGACGGAACAGTGAAGAGATTTTTATTAACCAATGGAAATGTGATTGAAGGGG

ATGTTTATGTGTTTGCCACTCCAGTTGATATCCTGAAGCTTCTCTTGCCTGACAGCTGGAAAGAAATTCC

ATATTTTAAAAAGTTGGAGAAATTAGTAGGAGTCCCAGTTATTAATGTTCACATATGGTTTGACAGAAAA

CTGAAGAACACATATGACCACCTACTTTTTAGCAGAAGTCCTCTTCTAAGTGTCTATGCTGACATGTCTG

TAACATGCAAGGAATATTACAATCCAAACCAGTCTATGTTGGAGTTGGTTTTTGCACCAGCAGAAGAATG

GATATCCCGTAGTGACACAGAAATTATTGATGCTACGATGAAGGAACTTGCTAAACTCTTTCCTGATGAA

ATATCCGCCGATCAGAGCAAAGCAAAGATTTTAAAGTATCATGTTGTCAAAACACCGAGGTCTGTCTACA

AAACCGTCCCTGATTGTGAACCTTGCCGGCCCTTGCAAAAATCTCCCGTAGAGGGTTTTTATTTGGCTGG

TGACTACACAAAACAGAAATATTTAGCTTCGATGGAAGGTGCAGTTCTATCGGGAAAGCTTTGTGCACAG

GCAATTGTACAGGATTACGATTCACTTATTGCTCGTGAGCAAAGGAACTTGGCCGAGGCAGTCAGTCGTT

GA

>Malus domestica

ATGGCGCAGTGGGCTTGTGTCTCCGCTGCTAACTTGAGCTGCCAAGCTACCATCGTAAACACTCAAAAGC

AACGAAACAGTCCCGGATGCGATGCCCTTTCTTTCAAAGGCAGTGAATTTATGGCTCAGAGCTGTAGATT

TTCAAGCCCACAAGCTGTTTATAGAAGGCCCAGGAATGGTGTTTGCCCCTTGAAGGTGGTTTGCGTTGAT

TATCCAAGACCAGACCTTGACAGTACTGCTAATTTCTTAGAAGCTGCGTACTTCTCTTCCACTTTCCGAG

CCTCTCCTCGTCCAACCAAGCCGTTAAAAGTTGTGATTGCTGGTGCAGGTTTGGCTGGTCTGGCAACTGC

AAAATATTTGGCGGATGCGGGTCATCAACCTATACTACTAGAAGCGAGAGATGTTTTAGGCGGAAAGGTG

GCAGCATGGAAAGATAGTGATGGGGACTGGTATGAAACAGGCCTGCATATATTCTTTGGGGCATATCCAA

ATATTCAGAATCTGTTTGGAGAGCTTGGTATTAATGATCGGTTGCAGTGGAAGGAACATTCTATGATATT

TGCAATGCCAAACAAGCCAGGGGAGTTCAGTCGGTTTGATTTCCTGGAAGTTCTGCCAGCACCCATAAAT

GGAATATGGGCCATATTGAAGAACAATGAGATGCTGACTTGTCCAGAGAAAATCAAGTTTGCAATTGGAC

TACTGCCAGCAATCCTTGGTGGGCAGGCTTATGTTGAAGCCCAAGATGGCTTGAGCGTAAAAGACTGGAT

GAGGAAACAGGGCATACCTGATCGAGTAACTACAGAGGTGTTTATAGCCATGTCAAAGGCCCTTAACTTT

ATTAACCCTGATGAACTTTCAATGCAGTGCATATTGATTGCTTTGAACCGGTTCCTCCAGGAGAAACACG

GTTCCAAGATGGCTTTCTTGGATGGTAGTCCCCCCGAGAGACTCTGTGCTCCAATTGTTGATCATATCCA

GTCATTGGGCGGTGAAGTCCGAACTAATTCCCGAATACAGAAAATTGATCTAAATAACGATGGAACTGTG

AAGAGTTTTGTACTAAATAATGGGAGCGTGATTGAAGCAGATGCGTATGTGTTCGCCACTCCAGTTGATA

TCCTAAAGCTTCTATTGCCTGAAAACTGGAAAGAGATGCCATATTTCAAGAAATTGGAGAAATTAGTTGG

AGTTCCAGTTATCAATGTTCACATATGGTTCGACAGAAAGCTGAAGAACACATATGATCACCTACTTTTT

AGCAGAAGTCCTCTTTTAAGTGTGTATGCTGACATGTCCGTAACATGTAAGGAATATTACAATCCAAACC

AATCTATGCTGGAGTTGGTTTTTGCACCGGCAGAAGAATGGATTTCATGTAGTGATTCTGAAATTATTGA

TGCTACACTCAAAGAACTTGCAAAACTCTTTCCTGACGAAATAGCTGCAGATCAGAGCAAAGCAAAGATT

TTGAAGTACCATGTTGTGAAAACACCAAGGTCTGTTTACAAGACTGTACCAGGTTGTGAACCTTGCCGTC

CCTTGCAAAGATCTCCCCTAGAGGGTTTCTATTTAGCTGGTGATTACACAAAACAAAAGTATTTAGCCTC

AATGGAAGGAGCAGTTCTATCAGGGAAACTTTGTGCTCAGGCGATTGTACAGGATTATGAATTGCTTGCT

GCCCGGGGAATAAAAACAACGTTGGCTGAGGCAGCCGCTCGATGA

>Durio zibethinus

ATGATTTTATGTCGTGTTTCCCTCTATTTCGGCCAAATCCACTCTAAAAAAAAATACGTACACGTTAACT

TATCACCTCCACAACTTCCTCTGCCTGTGTCCCGAACCACTTTCCATATCCCACTTTCTATTATCAAGGC

AAATCAAGTTTTTTTAACAATTTTTTTTGAAATAATTCAATCTACCCCTTCTTTGGCGTTGATTTGTTGG

TGTTCGAAAATGAGTCTCTGTGGGAGTGTTTCTGCTGTGCATTTGAACTTCCAAAGCAACACAATAAGCA

TGGGAAGCGTCTTAGCTTTTAGAAGTGGTGAATCCATGGGACATACCTTGAGAATTCCTTTTAAAAAGGG

GTCAAGTAAGGGTGCTTGCCCTTTGCAGGTGCTTTGTATAGATTATCCAAGACCTGAGCTTGAGAATACT

GTTAACTTTTTGGAGGCTGCGTCTTTATCTGCTTCTTTTCGTTCTGCTCCCCGTCCAACTAAGCCATTGA

AAGTCATAATTGCTGGTGCAGGTTTGGCTGGTTTGTCAACTGCAAAATATTTAGCAGATGCAGGTCACAA

ACCTCTGTTGCTAGAAGCAAGAGATGTTCTAGGTGGAAAGGTGGCTGCATGGAGAGATGAGGATGGAGAT

TGGTATGAGACAGGCCTACATATATTCTTTGGGGCTTACCCAAATGTGCAAAACCTGTTTGGAGAACTTG

GCATCAATGATCGGTTGCAATGGAAGGAGCATTCTATGATATTTGCAATGCCAAATAAACCTGGAGAGTT

CAGCCGATTTGATTTTCCAGAAGTTCTACCTGCACCTTTAAATGGGATATGGGCCATTTTGAAGAACAAT

GAAATGCTGACTTGGCCAGAGAAAGTGAAGTTTGCAATAGGACTCCTACCAGCAATGCTTGGTGGACAAC

CTTATGTTGAGGCCCAAGATGGTTTAAGTGTTAAAGAGTGGATGAAAAAGCAGGGTGTACCTGATCGTGT

GACTAACCAGGTGTTTATTGCCATGTCAAAGGCACTGAACTTCATTAACCCAGATGAACTTTCAATGCAA

TGTATACTGATTGCTTTAAACAGATTTCTTCAGGAGAAAAATGGATCCAAGATGGCATTCTTGGATGGCA

ACCCTCCAGAGAGGCTTTGCATGCCTATTGTTAATCATATTGAATCACTGGGTGGCGAGGTCCGGCTGAA

CTCACGAATAAAGAAAATAGAGCTTAATGATGATGGGACTGTGAAGAGTTTTCTTCTAACTGATGACAAT

ACAATTGAAGGAGATGCTTATGTAATTGCAACTCCAGTTGATATCCTAAAGCTACTTTTGCCTGAAGACT

GGAGAGAGATTTCATACTTCAAGAAATTAGAGAAATTAGTTGGCGTTCCAGTTATCAATGTTCACATATG

GTTTGATAGGAAATTGAAGAACACCTATGATCATCTACTCTTTAGCAGAAGTCCCCTTTTAAGTGTATAT

GCCGACATGTCCGTAACATGTAAGGAATATTACAATCCAAACCAATCCATGTTGGAGTTAGTTTTTGCTC

CTGCAGAAGAATGGATTGCACGAAGTGACTCAGAAATTATTGACGCTACAATGAAGGAACTTGCAAAGCT

CTTTCCTGATGAAATTTCTGCAGATCAGAGCAAAGCAAAAGTCGTAAAGTACCATGTTGTTAAAACACCA

AGATCTGTATATAAAACTGTACCAAATTGTGAACCCTGCCGTCCCTTGCAAAGATCTCCAATAGAGGGAT

TCTATCTAGCGGGTGATTACACAAAGCAAAAGTATTTAGCTTCGATGGAAGGTGCTGTTCTTTCAGGGAA

GCTTTGTGCAGAGTCTATTGTACAGGATTATGAATTGCTTTGTACTTTGGGGCAAAGAAAGTTGACAGGA

GCAAGGGTTCACTGA

>Pyrus x bretschneideri

ATGGCGCAGTGGGCTTGTGTCTCCGCTGCTAACTTGAGCTGCCAAGCTACCATCGTAAACACTCAGAAGC

AACGAAACAGTCCCCGATGCGATGCCTTTTCTTTCAAAGGCAGTGAATTTATGGCTCAGAGCTGGAGATT

TTCAAGCCCACAAGCTGTTTATAGAAGGCCCAGGAATGGTGTTTGCCCTTTGAAGGTCGTTTGCGTTGAT

TATCCAAGACCAGACCTTGACAGTACTGCTAATTTCTTAGAAGCTGCGTACTTCTCTTCCACTTTCCGAG

CCTCTCCTCGTCCAACCAAGCCGTTAAAAGTTGTGATTGCTGGTGCAGGTTTGGCTGGTCTGGCAACTGC

AAAATATTTGGCGGATGCGGGTCATCAACCTATACTACTAGAAGCCAGAGATGTTTTAGGCGGAAAGGTG

GCAGCATGGAAAGATAGTGATGGGGACTGGTACGAAACAGGCCTCCATATATTCTTTGGAGCATATCCAA

ATATTCAGAACCTGTTTGGAGAGCTTGGTATTAATGATCGATTGCAGTGGAAGGAACATTCTATGATATT

TGCAATGCCAAACAAGCCAGGGGAGTTCAGTCGGTTTGATTTCCTAGAAGTTCTGCCAGCACCCATAAAT

GGAATATGGGCCATATTGAAGAACAATGAGATGCTGACTTGGCCAGAGAAAATCAAGTTTGCAATTGGAC

TACTGCCAGCAATCCTTGGTGGGCAGGCTTATGTTGAAGCCCAAGATGGCTTGAGCGTAAAAGACTGGAT

GAGGAAACAGGGCATACCTGATCGAGTAACTACTGAGGTGTTTATAGCTATGTCAAAGGCCCTTAACTTT

ATTAACCCTGATGAACTTTCAATGCAATGCATATTGATTGCTTTGAACCGATTTCTTCAGGAGAAACACG

GTTCCAAGATGGCTTTCTTGGATGGTAGTCCCCCTGAGAGACTCTGTGCTCCAATTGTTGATCATATCCA

GTCATTGGGCGGTGAAGTCCGAACTAATTCCCGAATACAGAAAATTGATCTAAATAATGATGGAACTGTA

AAGAGTTTTGTACTAAATAATGGGAGTGTGATTGAAGCAGATGCGTATGTGTTTGCCACTCCAGTTGATA

TCTTAAAGCTTCTATTGCCTGAAAACTGGAAAGAGATGCCATATTTCAAGAAATTGGAGAAACTAGTTGG

AGTTCCAGTTATCAATGTTCACATATGGTTTGATAGAAAGCTGAAGAACACATATGATCACCTACTTTTT

AGCAGAAGTCCTCTTTTAAGTGTGTATGCTGACATGTCCGTAACATGTAAGGAATATTACAATCCAAACC

AATCTATGCTGGAGTTGGTTTTTGCACCGGCAGAAGAATGGATTTCATGTAGTGATTCAGAAATTATTGA

TGCTACCCTCAACGAACTTGCAAAACTCTTTCCTGACGAAATAGCTGCAGATCAAAGCAAAGCAAAGATT

TTGAAGTACCATGTTGTGAAAACACCAAGGTCTGTTTACAAGACTGTACCAGGTTGTGAACCTTGCCGTC

CCTTGCAGAGATCTCCCCTAGAGGGTTTCTATTTAGCTGGTGATTACACAAAACAAAAGTATTTAGCCTC

AATGGAAGGAGCGGTTCTATCAGGGAAACTTTGTGCTCAGGCGATTGTACAGGATTATGAATTGCTTGCT

GCCCGGGGAAAAAAAACAAGGTTGCCTGAGGCAGCTGCTCGATGA

>Rosa chinensis

ATGTCGCAGTGGGCTTGTGTCTCTGCCACCAACTTGAGCTACCAAGCCAACCTCATCAACACCCAAAACC

CACAAACCACTCCCAGATATGATGCGCTTTCCTTTCACGGCAGTGAAATTGTTGCTCGGAATTTTGGGTT

TCTGAGCTCACAAGCTACTACTAGTATTGGTAAAAGGCTGAGGAAGGGTGCTCTCCCTTTGAAGGTGGTT

TGTGTGGATTATCCAAGACCCGAGCTTGACAATACTGTAAATTTCTTAGAAGCTGCGCTCTTGTCTTCCT

CTTTCAGAGCCTCTTCTCGCCCAGCTAAGCCCCTCAAGGTTGTGATTGCTGGTGCAGGTTTGGCTGGGTT

GTCAACTGCAAAGTATTTGGCAGATGCAGGTCATAAACCCATACTACTGGAAGCAAGAGACGTTTTAGGT

GGAAAGATTGCAGCATGGAAAGATAAAGATGGAGACTGGTATGAGACAGGCCTACATATATTTTTTGGGG

CTTATCCAAATATTCAGAACTTGTTTGGAGAGCTTGGTATCGATGATCGGTTGCAGTGGAAGGAACACTC

TATGATATTTGCAATGCCAAACAAGCCAGGAGAGTTCAGCCGGTTTGATTTCCCTGAAGTTCTGCCAGCA

CCCTTAAATGGAATATGGGCCATATTAAAGAACAATGAGATGCTGACATGGCCAGAAAAAGTGAAGTTTG

CTATCGGACTTGTGCCAGCAATTCTTGGTGGACAGGCTTATGTTGAAGCTCAGGATGGCTTGACTGTAAA

GGAGTGGATGAGAAAACAGGGGATACCTGATCGAGTAACTACTGAGGTGTTTATTGCCATGTCAAAGGCC

CTTAACTTTATTAATCCTGATGAGCTCTCAATGCAATGCATATTGATTGCTTTGAATCGATTTCTTCAGG

AGAAACACGGTTCCAAGATGGCTTTCCTTGATGGAAGTCCTCCCGAGAGACTCTGTCAACCAATCGTTGA

TCATATCCAGTCATTGGGCGGTGAAGTCCGGCTTAATTCCCGATTACAAAAGATTGAGCTAAATAATGAT

GGAACAGTGAAGAGCTTTGTACTACAAAATAACAGTGTGATTGAAGCGGATGCTTATGTATCTGCCTCTC

CAGTTGATATCTTCAAGCTTCTAGTGCCTGAAAACTGGAAAGAGATTCCATATTTCAAGAAATTGGACAA

ACTAGTTGGAGTTCCAGTCATCAATGTACACATATGGTTTGACAGAAAACTAAAGAACACATATGATCAC

CTACTTTTTAGCAGAAGTCCTCTTCTAAGTGTGTATGCTGATATGTCGGTAACATGCAAGGAGTATTACA

ATCCAAATCAGTCTATGCTGGAGTTGGTTTTTGCACCAGCAGAAGAATGGATTTCACGCAGTGATTCGGA

AATTATTGATGCTACGCTCAAAGAACTTGCAAAGCTCTTTCCCGATGAGATAGCTGCAGATCAAAGCAAA

GCGAAGATTTTGAAGTACCATGTTGTGAAAACACCAAGGTCTGTGTACAAAACTATACCAGATTGTGAAC

CTTGCCGTCCGTTGCAAAGATCTCCCTTGGAGGGTTTCTATTTAACTGGTGACTATACAAAACAAAAATA

CTTAGCCTCTATGGAAGGTGCTGTTCTATCAGGGAAACTTTGTGCACAGGCGATTGTACAGGACTATGAA

TTGCTCGTTGCTCGGGGCCAGAAAAGGTTGGCTGAGGCAGGTGCTAGATGA

>Prunus mume

ATGTCTCAGTGGGCTTGTGTCTCTGCTGCTAACTTGAGCTGCCAAGCTAGCATCATCAACACTCAAAAGC
TACGAAACACTCCTAGATGCGATGACTTTTCATTTAAAGGTAGTGAATTTATGGCTCAAAGCTGTAGATT
TTTAAGCCCACAAGCTATTTATGGAAGGCCGAGGAATGGTGCTTGCCCTTTGAAGGTGGTTTGCGTTGAT
TATCCAAGACCAGACCTTGACAATACTGCTAATTTCTTAGAAGCTGCATATTTCTCTTCCACTTTCCGAG
CCTCTCCTCGTCCAGCTAAGCCGTTGAAGGTCGTGATTGTTGGTGCAGGTTTGGCTGGTCTGGCAACTGC
AAAATATCTGGCTGATGCAGGTCATAAACCTATCTTACTGGAAGCAAGAGATGTTCTAGGCGGAAAGGTG
GCAGCATGGAAAGATAAGGATGGAGACTGGTACGAAACAGGCCTGCATATCTTCTTTGGGGCTTATCCGA
ATATTCAGAACCTGTTTGGTGAGCTTGGTATTGATGATCGATTGCAGTGGAAGGAGCATTCTATGATATT
TGCAATGCCAAGCAAACCAGGAGAGTTCAGCCGGTTTGATTTCCCTGAAGTTTTACCAGCACCCTTAAAT
GGAATATGGGCCATATTGAAGAACAATGAGATGCTGACTTGGCCAGAGAAAATCAAGTTTGCAATTGGAC
TACTGCCAGCAATTCTTGGTGGGCAGGCTTATGTTGAAGCCCAAGATGGCTTGAGTGTAAAAGATTGGAT
GAGGAAACAGGGCATACCGGATCGAGTGACTACTGAGGTGTTTATTGCCATGTCAAAGGCCCTGAACTTT
ATTAACCCTGATGAACTTTCAATGCAATGCATATTGATTGCTTTGAACCGATTCCTTCAGGAGAAACACG
GTTCCAAGATGGCTTTTTTGGATGGTAGTCCCCCTGAGAGACTCTGTGCACCAATTGTTGATCATATCCA
GTCATTAGGCGGTGAAGTCCGAATTAATTCCCGAATACAGAAAATTGAGCTAAATAAAGATGGGACCGTG
AAGAGTTTTGTACTAAATAATGGGAGCATGATTGAAGCAGATGCCTATGTATTCGCCACTCCAGTTGATA
TCCTAAAGCTTCTATTGCCTGATAACTGGAAAGAGATCCCATATTTCAAGAAATTGGAGAAACTAGTTGG
CGTTCCAGTTATCAATGTTCACATATGGTTTGACAGAAAGCTGAAGAACACATATGATCATCTACTTTTT
AGCAGAAGTGCTCTTTTAAGTGTCTATGCTGACATGTCTGTAACATGTAAGGAATATTACAATCCAAACC
AGTCTATGCTGGAGTTGGTTTTTGCACCAGCAGAAGAATGGATTTCACGCAGTGATTCAGAAATTATTGA
TGCTACACTCAAAGAACTTGCAAAACTCTTTCCTGATGAGATAGCTGCAGATCAAAGCAAAGCAAAGATT
TTGAAGTACCATGTTGTGAAAACACCAAGGTCGGTTTACAAAACTGTACCAGGTTGTGAACCTTGCCGTC
CCTTGCAAAGATCTCCCCTAGAGGGTTTCTATTTAGCTGGTGATTACACAAAACAAAAGTATTTAGCCTC
AATGGAAGGTGCTGTTCTGTCAGGGAAACTTTGTGCCCAAGCAATTGTACAGGATTACGAATTGCTTGTT
GCTCGGGGACAAACAAGGGTGGCTGAGGCAAGCGTTCGGTGA

>Prunus armeniaca

ATGTCTCAGTGGGCTTGTGTCTCTGCTGCTAACTTGAGCTGCCAAGCTAGCATCATCAACACTCAAAAGC
TACGAAACACTCCCAGATGCGATGCCTTTTCATTTAAAGGTAGTGAATTTATGGCTCAAAGCTGTAGATT
TTTAAGCCCACAAGCTATTTATGGAAGGCCGAGGAATGGTGCTTGCCCTTTGAAGGTGGTTTGCGTTGAT
TATCCAAGACCAGACCTTGACAATACTGCTAATTTCTTAGAAGCTGCATATTTCTCTTCCACTTTCCGAG
CCTCTCCTCGTCCAGCTAAGCCGTTGAAGGTCGTGATTGCTGGTGCAGGTTTGGCTGGTCTTGCAACTGC
AAAATATTTGGCTGATGCAGGTCATAAACCTATCTTACTGGAAGCAAGAGATGTTCTAGGCGGAAAGGTG
GCAGCATGGAAAGATAAGGATGGAGACTGGTACGAAACAGGCCTCCATATCCTCTTTGGGGCTTATCCGA

ATATTCAGAACCTGTTTGGTGAGCTTGGTATTGATGATCGATTGCAGTGGAAGGAGCATTCTATGATATT

TGCAATGCCAAACAAACCAGGAGAATTCAGCCGGTTTGATTTCCCTGAAGTTTTACCAGCACCCTTAAAT

GGAATATGGGCCATATTGAAGAACAATGAGATGCTGACTTGGCCAGAGAAAATAAAGTTTGCAATTGGAC

TACTGCCAGCAATTCTTGGTGGGCAGGCTTATGTTGAAGCCCAAGATGGCTTGAGTGTAAAAGATTGGAT

GAGGAAACAGGGCATACCGGATCGAGTGACTACTGAGGTGTTTATTGCCATGTCAAAGGCCCTGAACTTT

ATTAACCCTGATGAACTTTCAATGCAATGCATATTGATTGCTTTGAACCGATTCCTTCAGGAGAAACACG

GTTCCAAGATGGCTTTCTTGGATGGTAGTCCCCCTGAGAGACTCTGTGCACCAATTGTTGATCATATCCA

GTCATTGGGCGGTGAAGTCCGAATTAATTCCCGAATACAGAAAATTGAGCTAAATAAAGATGGGACCGTG

AAGAGTTTTGTACTAAATAATGGGAGCATGATTGAAGCAGATGCCTATGTATTTGCCACTCCAGTTGACA

TCCTAAAGCTTCTATTGCCTGATAACTGGAAAGAGATCCCATATTTCAAGAAATTGGAGAAACTAATTGG

CGTTCCAGTTATCAATGTTCACATATGGTTTGACAGAAAGCTGAAGAACACATATGATCATCTACTTTTT

AGCAGGAGTCCTCTTTTAAGTGTCTATGCTGACATGTCCGTAACATGTAAGGAATATTATAATCCAAACC

AGTCTATGCTGGAGTTGGTTTTTGCACCAGCAGAAGAATGGATTTCATGCAGTGATTCAGAAATTATTGA

TGCTACACTCAAAGAACTTGCAAAACTCTTTCCTGATGAGATAGCTGCAGATCAGAGCAAAGCAAAGATT

TTGAAGTACCATGTTGTGAAAACACCAAGGTCGGTTTACAAAACTGTACCAGATTGTGAACCTTGCCGTC

CCTTGCAAAGATCTCCCCTAGAGGGTTTCTATTTAGCTGGTGATTACACAAAACAAAAGTATTTAGCCTC

AATGGAAGGTGCTGTTCTGTCAGGGAAACTTTGTGCACAAGCAATTGTACAGGATTACGAATTGCTTGTT

GCTCGGGGACAAACAAGGTTGGCTGAGGCAAGCGTTCGGTGA

>Prunus dulcis

ATGTCTCAGTGGGCTTGTGTCTCTGCTGCTAACTTGAGCTGCCAAGCTAGCATCATCAACACTCAAAAGC

TACGAAACACTCCCAGATGCGATGCCTTTTCATTTAAAGGTAGTGAGTTTATGGCTCAAAGCTGTAGATT

TTTAAGCCCACAAGCTATTTATGGAAGGCCGAGGAATGGTGCTTGCCCTTTGAAGGTGGTTTGCGTTGAT

TATCCAAGACCAGACCTTGACAATACTGCTAATTTCTTAGAAGCTGCATATTTCTCTTCCACTTTCCGAG

CCTCTCCTCGTCCAGCTAAGCCGTTGAAGGTCGTGATTGCTGGTGCAGGTTTGGCTGGTCTGGCAACTGC

AAAATATTTGGCTGATGCAGGTCATAAACCTATCTTACTGGAAGCAAGAGATGTTCTAGGCGGAAAGGTG

GCAGCATGGAAAGATAAGGATGGAGACTGGTACGAAACAGGCCTACATATCTTCTTTGGGGCTTATCCGA

ATATTCAGAACCTGTTTGGTGAGCTTGGTATTGATGATCGATTGCAGTGGAAGGAGCATTCTATGATATT

TGCAATGCCAAGCAAACCAGGAGAGTTCAGCCGGTTTGATTTCCCTGAAGTTTTACCAGCACCCTTAAAT

GGAATATGGGCCATATTGAAGAACAATGAGATGCTGACTTGGCCAGAGAAAATCAAGTTTGCAATTGGAC

TACTGCCAGCAATTCTTGGTGGGCAGGCTTATGTTGAAGCCCAAGATGGCTTGAGTGTAAAAGATTGGAT

GAGGAAACAGGGCATACCGGATCGAGTGACTACTGAGGTGTTTATTGCCATGTCAAAGGCCCTGAACTTT

ATTAACCCTGATGAACTTTCAATGCAGTGCATATTGATTGCTTTGAACCGATTCCTTCAGGAGAAACACG

GTTCCAAGATGGCTTTTTTGGATGGTAGTCCCCCTGAGAGACTCTGTGCACCAATTGTTGATCATATCCA

GTCATTAGGCGGTGAAGTCCGAATTAATTCCCGCATACAGAGAATTGAGCTAAATAAAGATGGGACCGTG

AAGAGTTTTGTACTAAATAATGGGAGCATGATTGAAGCAGATGCCTATGTATTCGCCACTCCAGTTGATA

TCCTAAAGCTTCTATTGCCTGATAACTGGAAAGAGATCCCATATTTCAAGAAATTGGAGAAACTGGTTGG

CGTTCCAGTTATCAATGTTCACATATGGTTTGACAGAAAGCTGAAGAACACATATGATCATCTACTTTTT

AGCAGAAGTCCTCTTTTAAGTGTCTATGCCGACATGTCCGTAACATGTAAGGAATATTACAATCCAAACC

AGTCTATGCTGGAGTTGGTTTTTGCACCAGCAGAAGAATGGATATCATGCAGTGATTCAGAAATTATTGA

TGCTACACTCAAAGAACTTGCAAAACTCTTTCCTGATGAGATAGCTGCAGATCAAAGCAAAGCAAAGATT

TTGAAGTACCATGTTGTGAAAACACCAAGGTCGGTTTACAAAACTGTACCAGGTTGTGAACCTTGCCGTC

CCTTGCAAAGATCTCCCCTAGAGGGTTTCTATTTAGCTGGTGATTACACAAAACAAAAGTATTTAGCCTC

AATGGAAGGTGCTGTTCTGTCAGGGAAACTTTGTGCACAAGCAATTGTACAGGATTACGAATTGCTTGTT

GCTCGGGGACAAACAAGGGTGGCTGAGGCAAGCGTTCGGTGA

>Viola philippica

ATGAGTGTACACGGGAGTGTTTCTGCGTTGAGCTTGACCGGCCATGGTAGCACCTTAAACGTTAGAAACT

CACAACCTGGGTTGAGATACCGCCATCCTGCTTGTTTTAGGCAAAGCAGTACACTTGCTTTCACCAGGAG

TGAATCAATGGGTCATGCTTTCAAATTCTCAGCTGGAAATGCTCCTTGTAGCAGAGCAAGAAGTAATGTT

GGCCGTCTGCGGGTAGTATGTGTGGACTATCCAAGGCCTGAGATTGATAACACCACAAACTTCTTGGAAG

CTGCCTTCTTGTCATCAACATTTCGCACTTCTCCACGTCCAGCTAAACCCTTGAAAGTTGTAATTGCTGG

TGCAGGTCTGGCTGGTTTATCAACTGCAAAATATTTGGCAGATGCAGGCCACAAGCCTCTATTACTGGAA

GCAAGAGATGTTCTAGGTGGAAAGGTGGCTGCTTGGAAAGATGACGATGGAGACTGGTACGAGACTGGAT

TGCATATATTCTTTGGAGCGTACCCAAATATTCAGAACTTGTTTGGAGAGCTTGGCATTAATGATAGGTT

GCAGTGGAAGGAGCATTCCATGATATTTGCAATGCCAAACAAACCAGGAGAGTACAGCCGATTCGATTTC

CCCGATGCTCTTCCCGCACCAATCAATGGGATATTGGCCATTTTGAAGAACAATGAAATGCTGACCTGGC

CAGAGAAAGTGAAGTTTGCAATTGGACTCCTTCCAGCAATGCTTGGTGGACAGGCTTATGTTGAGGCTCA

AGATGGTCTAAGTGTTCAAGAGTGGATGAGAAAGCAGGGGGCACCTGATCGAGTTACTACCGAGGTGTTT

ATTGCTATGTCAAAGGCATTAAACTTCATTAACCCAGATGAACTGTCAATGCAGTGTATATTGATAGCTT

TGAACCGGTTTCTTCAGGAGAAACATGGTTCAAAGATGGCTTTTTTAGATGGTAATCCACCAGAGAGACT

CTGCATGCCAATTGTTGATCATATTCAGTCACTTGGTGGTGAAGTCCGGCTGAATTCCCGCATAAAGAAA

ATTGAGCTAAATGATGATGGTACAGTGAAGAACTTTTTATTAAATAGTGGGGACGTGATTGAAGGAGATG

TTTATGTATTTGCTACTCCAGTTGATATCCTGAAGCTTCTTTTGCCTGATAACTGGAAGGAGATTCCTTA

CTTCAAGAAATTGGAGAAATTAGTTGGAGTTCCCGTTATTAATGTTCACATATGGTTTGACAGGAAACTG

AAGAATACATATGATAGCCTACTTTTTAGCAGAAGCCCCCTTCTTAGTGTATATGCTGACATGTCGGTAA

CATGTAAGGAATATTACGACCCAAATAAATCTATGCTGGAATTAGTTTTTGCACCTGCAGAAGAATGGAT

CTCACGGACTGATTCCGAGATTATTGATGCTACAATGAAAGAACTTGCAAAACTCTTTCCTGATGAAATA

GCCGCTGATCAAAGCAAAGCAAAAATTGTCAAGTACCATGTTGTGAAAACTCCAAGGTCTGTTTACAAGA

CTGTCCCGAATTGCGAACCTTGTCGGCCTTTACAAAGATCACCTATGGAGGGCTTCTATTTATCTGGTGA

CTACACGAAACAAAAATATTTGGCATCAATGGAAGGCGCCGTTCTATCGGGGAAGCTGTGTGCACAAGCA

ATTATGCAGGATTACGAGTTACTTGCTGGTCTTGGGCAGAGAACGCTGGCGGAGGCAACCATTAGTTAG

>Prunus persica

ATGTCTCAGTGGGCTTGTGTCTCTGCTGCTAACTTGAGCTGTCAAGCTAGCATCATCAACACTCAAAAGC

TACGAAACACTCCCAGATGCGATGCCTTTTCATTTAAAGGTAGTGAATTTATGGCTCAAAGCTGTAGATT

TTTAAGCCCACAAACTATTTATGGAAGGCCGAGGAATGGTGCTTGCCCTTTGAAGGTGGTTTGCGTTGAT

TATCCAAGACCAGACCTTGACAATACTGCTAATTTCTTAGAAGCTGCATATTTCTCTTCCACTTTCCGAG

CCTCTCCTCGTCCAGCTAAGCCGTTGAAGGTCGTGATTGCTGGTGCAGGTTTGGCTGGTCTGGCAACTGC

AAAATATTTGGCTGATGCAGGTCATAAACCTATCTTACTGGAAGCAAGAGATGTTCTGGGCGGAAAGGTG

GCAGCATGGAAAGATAAGGATGGAGACTGGTACGAAACAGGCCTACATATCTTCTTTGGGGCTTATCCGA

ATATTCAGAACCTGTTTGGTGAGCTTGGTATTGATGATCGATTGCAGTGGAAGGAGCATTCTATGATATT

TGCAATGCCAAGCAAACCAGGAGAGTTCAGCCGGTTTGATTTCCCTGAAGTTTTACCAGCACCCTTAAAT

GGAATATGGGCCATATTGAAGAACAATGAGATGCTGACTTGGCCAGAGAAAATCAAGTTTGCAATTGGAC

TACTGCCAGCAATTCTTGGTGGGCAGGCTTATGTTGAAGCCCAAGATGGCTTGAGTGTAAAAGATTGGAT

GAGGAAACAGGGCATACCGGATCGAGTGACTACTGAGGTGTTTATTGCCATGTCAAAGGCCCTGAACTTT

ATTAACCCTGATGAACTTTCAATGCAATGCATATTGATTGCTTTGAACCGATTCCTTCAGGAGAAACACG

GTTCCAAGATGGCTTTTTTGGATGGTAGTCCCCCTGAGAGACTCTGTGCACCAATTGTTGATCATATCCA

GTCATTAGGCGGTGAAGTCCGAATTAATTCCCGAATACAGAGAATTGAGCTAAATAAAGATGGGACCGTG

AAGAGTTTTGTACTAAATAATGGGAGCATGATTGAAGCAGATGCCTATGTATTCGCCACTCCAGTTGATA

TCCTAAAGCTTCTATTGCCTGATAACTGGAAAGAGATCCCATATTTCAAGAAATTGGAGAAACTGGTTGG

CGTTCCAGTTATCAATGTTCACATATGGTTTGACAGAAAGCTGAAGAACACATATGATCATCTACTTTTT

AGCAGAAGTCCTCTTTTAAGTGTCTATGCCGACATGTCCGTAACATGTAAGGAATATTACAATCCAAACC

AGTCAATGCTGGAGTTGGTTTTTGCACCAGCAGAAGAATGGATATCATGCAGTGATTCAGAAATTATTGA

TGCTACACTCAAAGAACTTGCAAAACTCTTTCCAGATGAGATAGCTGTAGATCAAAGCAAAGCAAAGATT

TTGAAGTACCATGTGGTGAAAACACCAAGGTCGGTTTACAAAACTGTACCAGGTTGTGAACCTTGCCGTC

CCTTGCAAAGATCTCCCCTAGAGGGTTTCTATTTAGCTGGTGATTACACAAAACAAAAGTATTTAGCCTC

AATGGAAGGTGCTGTTCTGTCAGGGAAACTTTGTGCACAAGCAATTGTACAGGATTACGAATTGCTTGTT

GCTCGGGGACAAACAAGGGTGGCTGAGGCAAGCGTTCGGTG

>Acer palmatum

ATGAGCCTCTGCGGAAGCGTTTCTGCTTTGAGCTTGAGATGTGGGTTTCGTCATGGTAATTCGGATATGA

TGAATGCTATGTCGTTTCGAGGGAGTGAATCCATGGGTCATCCTCTCACAATTCCAACTAAAACCAGACC

CAGAAAGGCTTCTCACCCTTTGCAGGTAGTTTGCCTGGACTATCCAAGACCAGAGCTTGAGACTACCGTT

AATTTCTTGGAAGATTCTTACTTGTCTTCGTCTTTTCGTGTTTCTCCTCGACCAACTAAGCCATTGAAGA

TTATAATTGCCGGTGCAGGTTTGGCTGGTTTATCAACTGCTAAATATTTGGCAGATGCAGGCCACAAACC

TTTGTTATTGGAAGCAAGAGACGTTCTAGGTGGAAAGGTGGCTGCCTGGAAAGATGAGGATGGAGACTGG

TATGAGACGGGCCTCCATATATTTTTTGGGGCTTATCCTAATGTACAGAACCTGTTTGGAGAACTTGGTA

TCAATGACCGGTTGCAGTGGAAAGAGCACTCTATGATATTTGCCATGCCTAACAAGCCAGGAGAGTTCAG

CCGATTTGATTTTCCTGAAGTTCTTCCTGCACCTATAAATGGTATATGGGCCATTTTAAAGAATAATGAA

ATGCTGACTTGGGCAGAGAAAGTTAAGTTTGCAATTGGTCTGCTTCCAGCAATTCTTGGTGGGCAGGCTT

ATGTTGAAGCTCAAGATGGTATAACTGTTAAGGAGTGGATGAGAAAGCAGGGCATACCCGACCGAGTGAC

TACTGAGGTGTTTATTGCCATGTCAAAGGCACTAAACTTCATTAACCCTGACGAACTATCGATGCAATGT

ATATTGATTGCTTTAAATCGATTTCTTCAGGAGAAGCATGGTTCCAAGATGGCATTCTTAGATGGTAATC

CTCCAGAGAGACTCTGCATGCCTATTGTTGATCACATTCGGTCACTGGGTGGTGAAGTCGAACTTAATTC

ACGAATACAGAAAATTGAACTAAATAGTGATGCAACTGTGAAGAATTTCTTACTAACTAACGGGGAGGTA

ATTGAAGGAGATGTTTATGTATTTGCCACTCCAGTTGATATCCTTAAGCTTCTTTTACCTGAAAGCTGGA

AAGAGATTCCTTACTTCAAGAAATTGGAGAAATTGGTTGGAGTTCCAGTTATTAATGTTCACATATGGTT

TGACAGGAAATTGAAAAACACGTCTGATCATCTACTATTTAGCAGAAGTCCCCTCCTAAGTGTGTATGCA

GACATGTCGGTAACATGCAAGGAATATTACAACCCTAACCAGTCCATGCTGGAGTTAGTTTTCGCCCCTG

CTGAAGAATGGATTTCTTGCAGTGACTCAGAAATCATTGATGCTACAATGAAGGAGCTTGCAAAATTGTT

TCCTGATGAAATTTCTGCTGATCAGAGCAAAGCAAAAATACTGAAGTACCATATTGTTAAAACACCAAGG

TCTGTATACAAAACTGTTCCAAATTGTGAACCTTGTCGTCCCTTGCAAAGATCTCCTGTAGAGGGATTCT

ATTTAGCTGGAGATTACACAAAACAGAAGTACCTAGCATCGATGGAAGGGGCTGTTTTGTCGGGGAAGTT

TTGTGCACAAGCTATTGTACAGGACTATGAACTGCTTGGTGCACGGGGCGAAGGGAGATTGGCTGAAGCA

AGTATTCGGTAA

>Herrania umbratical

ATGAGTCTCTGTGGGAGTGTTTCGGCTGTGTACTTGAACTCCCAAAGCAACACAATAAGCATGGGAAGTG

TCTTAGCTTTTAGAAGTGGTGAATCCATGGGACATGCCTTGAGAATTCCCGTTAAAAAGAGGTCAAGTAA

GGGTGCTTGTCCTTTGCAGGTAGTTTGCATAGATTATCCAAGGCCAGAGCTTGAGAATACTGTTAATTTT

TTGGAGGCTGCTTCTCTATCTGCTTCTTTTCGTTCTGCTCCCCGTCCAACTAAGCCATTGAAAGTTATAA

TTGCTGGTGCAGGTTTGGCTGGTTTGTCAACTGCAAAATATTTAGCAGATGCAGGTCACAAACCTCTGTT

GCTTGAAGCAAGAGATGTCCTAGGTGGAAAGGTGGGCGCATGGAAAGATGATGATGGAGATTGGTATGAG

ACAGGCTTACATATATTCTTCGGGGCTTATCCAAATGTGCAAAGCCTGTTTGGAGAACTTGGCATTAATG

ACCGGCTGCAATGGAAGGAGCACTCTATGATATTTGCAATGCCAAACAAACCTGGAGAGTTCAGCCGATT

TGATTTTCCAGAAGTTCTACCTGCACCCTTAAATGGGATATGGGCCATTTTGAAGAACAATGAAATGCTG

ACTTGGCCAGAGAAAGTGAAGTTTGCAATAGGACTCCTACCAGCAATGCTTGGTGGACAACCTTACGTTG

AGGCCCAAGATGGTCTAACTGTTAAAGAGTGGATGAGAAAGCAGGGCATACCGGATCGTGTGACTAACGA

GGTGTTTATTGCCATGTCAAAGGCACTGAACTTCATTAACCCAGATGAACTTTCAATGCAGTGTATATTG

ATTGCTTTGAATCGATTTCTGCAGGAGAAAAATGGATCAAAGATGGCATTCTTGGATGGCAACCCCCCTG

AGAGGCTTTGCATGCCTATTGTTAATCATATTGAGTCACTGGGTGGTGAGGTTCGGCTTAACTCACGAAT

AAAGAAAATAGAGCTCAATGATGATGGAACTGTGAAGAGTTTTCTTCTAACTAATGGCAATGCAATTGAA

GGAGATGCTTATGTAATGGCAGCTCCAGTTGATATCCTGAAGCTACTTTTGCCTGAAGACTGGAGAGAGA

TTTCATACTTCAAGAAATTAGAGAAATTAGTAGGAGTCCCAGTTATCAACGTTCACATATGGTTTGATAG

GAAACTGAAGAACACCTACGATCATCTACTCTTTAGCAGAAGTCCCCTTCTAAGTGTTTATGCTGACATG

TCTGTAACGTGTAAGGAATATTACAATCCAAACCAGTCCATGTTGGAGTTAGTTTTTGCCCCTGCAGAAG

AATGGATTGCACGTAGTGACTCGGAAATTATTGATGCTACAATGAAGGAGCTTGCAAAACTCTTTCCTGA

TGAAATTTCTGCAGATCAGAGCAAAGCAAAAGTTATAAAGTACCATATTGTTAAAACACCAAGATCAGTA

TATAAAACTGTTCCAGATTGTGAACCCTGCCGCCCATTGCAAAGATCTCCGATAGAGGGGTTCTATCTAG

CTGGTGATTACACAAAACAAAAGTATCTGGCTTCAATGGAAGGCGCTGTTCTCTCAGGGAAGCTTTGTGC

GCAGTCTATTGTACAGGATTATGAGTTGCTTCTTGCTCTGGGACAAAGAAAGTTGGCAGGAGCAAGCATT

CACTAG

>Morus notabilis

ATGTCTCAGTGGGGTTGTGTTTCCGCGGCCAACTTGGGCTGGCAAAAAACAAGCGCCGCCGTGGATGGTC

GGAACGGAGGGAACATGCCCAGATGCTGTTTCTATCTGGGTTCGCAGAAGATGGACTCTTTGGCTTTTGG

GTATGGAGAATTTTTGGCTCGCGGGTCGAGAATTTCGTCTTCTCGGGCTGTTGGTAGAAGACAGAAGAAG

GGTGTTTCGTTTTCGCCATTGAAGGTAGTTTGTGTGGATTATCCAAGGCCCGAGCTGGACAACACTGTTA

ACTTCTTAGAAGCTGCTTCCTTGTCTGCCTCTTTTCGTAGCTCTCCTCGTCCGGCTAAACCTTTGAAAGT

CGTGATTGCTGGTGCAGGTTTGGCCGGTTTATGTACTGCAAAATACTTGGCAGATGCAGGTCATAAACCT

CTATTACTGGAAGCCAGAGATGTTTTAGGTGGAAAGGTGGCAGCATGGAAAGACGATGATGGAGATTGGT

ATGAGACAGGGTTACATATATTCTTTGGAGCTTACCCAAATTTGCAGAACTTGTTTGGAGAGCTTGGAAT

TGATGATCGGTTACAATGGAAAGAGCATTCTATGATATTTGCAATGCCTAACAAACCTGGAGAGTTCAGC

CGATTTGATTTCCCTGAAGTGCTGCCAGCACCCTTAAATGGAATATGGGCCATCTTAAGGAACAATGAGA

TGCTGACATGGCCAGAGAAAGTCAAGTTTGCAATCGGACTGCTGCCAGCAATACTTGGTGGCCAGCCTTA

TGTTGAAGCACAAGATGGTTTAACCGTTAAAGAATGGATGATAAAACAGGGCATACCTGATCGCGTAACT

GATGAGGTGTTTATTGCCATGTCAAAGGCCCTAAACTTTATCAACCCTGATGAACTTTCAATGCAGTGTA

TATTGATTGCGTTAAACCGTTTTCTTCAGGAGAAGCATGGTTCCAAGATGGCCTTCTTGGATGGGAATCC

ACCAGAGAGACTCTGTATGCCGATAGTTGAGCATATCCAGTCATTGGGTGGTGAAGTCGAGCTTAATTCA

CGGATACAAAAGATTGACCTAAATGATGATGGAACAGTAAAGAGATTCTTACTAACTAATGGAAGTGCGA

TAGAAGGGGATGTATACGTTTTTGCGACTCCAGTTGATATCCTAAAGCTTTTATTGCCCGACAACTGGAA

AGAGATTCCATATTTCAAGAAATTGGAGAAATTAGTTGGAGTTCCCGTTATCAATGTTCACATATGGTTT

GACAGAAAATTGAAGAACACATATGATCACCTGCTTTTCAGCAGAAGTCCTCTCCTAAGTGTCTATGCCG

ATATGTCAGTAACGTGTAAGGAATATTACAGTCCAAACCAGTCTATGCTGGAGTTAGTTTTTGCACCAGC

TGAAGAATGGATTTCATGTAGTGACTCAGAAATTATTGATGCTACAATGAAGGAACTTGCTAAGCTCTTT

CCTGATGAAATAGCGGCAGATCAGAGTAAAGCAAAAATTTTGAAATACCATGTTGTAAAAACACCAAGGT

CTGTTTACAAAACTGTTCCAGATTGTGAACCTTGTCGTCCGTTGCAAAGATCTCCTATAGAAGGCTTCTA

TTTAGCAGGCGACTACACAAAACAGAAGTATTTGGCTTCAATGGAGGGTGCTGTTCTCTCAGGGAAGTTT

TGTGCACAGTCAATTGTACAGGATTATGAGTTGCTTGCTGCTCGTGGTCAAAGAAGTTTGGCGAAGGCTG

GAAGTTGGTAA

>Gossypiumhirsutum

ATGAGTCTCTGTGGGAGTGTTTCTGCCCTGTACTTAAACTTACAAAGCAGCAAGATAAGCATGGGAAATG

TCTTAGCTTTTAGAAGTGGTGAATCCATGGGAAATACCTTGAGAATTCCCTTTAAAAAGAGGTCACGTAA

GGGTGCTGGTTGTCCTTTGCAGGTAGTTTGCATAGATTATCCAAGGCCAGAGCTAGAGAATACTGTTAAT

TTTTTGGAGGCTGCTTCTCTATCTGCTTCTTTTCGTTCAGCTTCCCGTCCAACTAAACCATTGAAAGTCA

TAATTGCTGGTGCAGGTTTGGCTGGTTTGTCAACTGCAAAGTATCTTGCGGATGCAGGTCATACACCAAT

ATTATTAGAAGCGAGAGATGTTCTAGGTGGAAAGGTGGCTGCATGGAAGGATGATGATGGAGATTGGTAT

GAGACAGGATTACATATATTCTTTGGGGCTTACCCAAATGTGCAAAACTTGTTTGGAGAACTTGGCATTA

ATGATCGGCTGCAATGGAAGGAGCATTCTATGATATTTGCGATGCCAAATAAACCTGGAGAGTTCAGTCG

ATTTGATTTTCCAGAAGTTCTACCTGCACCATTAAATGGAATATGGGCCATTTTGAAGAATAATGAAATG

CTGACTTGGCCAGAGAAAGTGAAATTTGCAATAGGACTCCTACCAGCAATGCTGGGTGGACAACCTTATG

TTGAGGCCCAAGATGGTTTATCTGTTAAAGACTGGATGAGAAAGCAGGGCGTACCTGATCGTGTGACTGA

GGAGGTGTTTATTGCCATGTCAAAGGCACTGAACTTCATTAACCCTGATGAACTTTCAATGCAATGTATA

TTGATTGCATTGAATCGATTTCTTCAGGAGAAACATGGATCAAAGATGGCATTCTTGGATGGAAACCCTC

CCGAGAGGCTTTGCATGCCTATCGTCAATCATATTGAATCACTGGGGGGTGAGGTCCGGCTTAATTCACG

TATAAAAAAAATAGAGCTCAATGAAGATGGAACTGTGAAGACTTTTCTTCTAAATAATGGCAATACAATC

GAAGGAGATGCTTATGTAGTTGCAACGCCCGTTGATATCTTCAAGTTACTTTTGCCTGAAGACTGGAGAG

AGATTTCATACTTCAAGAAATTAGAGAAATTAGTTGGAGTTCCAGTTATCAACGTTCACATCTGGTTTGA

TAGGAAATTGAAGAACACCTATGATCATCTACTCTTTAGCAGAAGCCCGCTTTTAAGTGTTTATGCTGAC

ATGTCTGTAACATGTAAGGAATATTACAATCCAAACCAATCCATGTTGGAGTTAGTTTTTGCCCCAGCAG

AAGAATGGATTGCATGTAGTGACTCAGAAATTATTGATGCTACAATGAAGGAACTTGCAAAGCTCTTTCC

TGATGAAATATCTGCAGATCAGAGTAAAGCAAAAGTCGTAAAATACCATATTGTTAAAACACCAAGATCT

GTATATAAAACTGTTCCAAATTGTGAACCCTGCCGCCCCTTGCAAAGATCTCCAATACAGGGGTTCTATC

TAGCAGGTGATTACACAAAGCAAAAGTATTTAGCTTCGATGGAAGGTGCTGTGCTCTCAGGGAAGCTTTG

TGCACAGTCTATTGTACAGGATTATGAGTTGCTTTGTACTTTGGGACAAAGAAAGTTGACAGGAGCAAGC

ATTCACTGA

>Carica papaya

ATGACTTTATGCGGGAGTGTTTCTGCGGCGAGCTTCGGCTGCCAAAGCAACAGAATAGCTATTGGAAACC

TTCATTCTGCCGCCTCGAAATGTGGTTATCGAGACACTCTGGATCAAAACAACATACTAGCATTTAGGGT

TAGTGAATCCATTGGAGACGGCCTGAGAATTCCCGAAGCACGAGCTGTTAAGATTAGGTCCAGGAACGGT

GCCCGCCCTTTGCAGGTAGTTTGTGTAGATTACCCGAGACCAGAGCTTGATAATACTTTAAATTTCTTGG

AAGCAGCGTACTTGTCTTCATCCTTTCGGACTTCTCCCCGTCCATCGAGACCATTGAAGATCGTAATTGC

TGGTGCAGGTTTGGCTGGTTTATCGACTGCAAAATATTTGGCAGATGCAGGTCACAAGCCTTTGTTGCTG

GAAGCAAGAGATGTTCTAGGTGGAAAGGTGGCTGCATGGAAAGATGATGATGGAGACTGGTATGAGACAG

GCTTACATATATTCTTTGGGGCCTATCCAAATATGCAGAACTTGTTTGGAGAACTTGGTATCAATGATCG

GTTGCAGTGGAAGGAGCACTCAATGATATTTGCAATGCCAAACAAGCCTGGGGAATTCAGCCGATTTGAC

TTCCTTGAAGAATTGCCGGCACCTTTAAACGGAATTTGGGCAATTTTAAAAAATAATGAAATGCTGACTT

GGCCTGAGAAAGTGAAGTTTGCGATTGGGCTTCTGCCTGCAATGGTCGGTGGACAGGAGTATGTTGAGGC

TCAAGATGGTTTAAGTGTTCAAGAGTGGATGAGAAAGCAGGGCATACCTGACCGGGTGACTAATGAGGTG

TTTATCGCTATGTCAAAGGCACTAAACTTCATTAACCCAGATGAACTGTCAATGCAATGTATACTGATTG

CTTTGAACCGATTTCTTCAGGAGAAGCATGGTTCTAAGATGGCATTCTTAGATGGTAACCCTCCAGAAAG

ACTCTGTATGCCAATTGTTGATCATATCCTGTCACTAGGTGGTGAAGTGAAACTTAATTCTCGGATACAG

ACAATTGAGCTCAACAATGATGGAACTGTGAAGAGCTTTATACTAAATAGTGGGGATGTGATTGAAGGAG

ATGCTTATGTATTTGCCACTCCAGTTGATATCTTGAAGCTTCTTCTGCCTGAAAGCTGGAAAGAGATCCT

ATACTTCAAGAGATTGGAGAAATTAGTTGGCGTCCCTGTTATTAATGTTCACATATGGTTTGACAGGAAA

CTAAAGAACACATATGACCACCTACTCTTCAGCAGAAGTCCCCTCCTGAGTGTATATGCTGATATGTCTG

TAACATGTAAGGAATATTACAACCCAAATCAATCCATGCTGGAGTTAGTTTTTGCCCCTGCTGAAGAATG

GATTTCACGCAGTGATTCAGAAATTATTGATGCTACAATGAAGGAACTTGCAAAACTTTTTCCTGATGAA

ATTGCAGCGGACCAAGGCAAAGCAAAAATATTGAAATACCATGTTGTTAAAACACCAAGGTCTGTCTACA

AAACTGTCCCAGGTTGTGAACCTTGCCGCCCGGTACAGAGAACTCCTATAGAGGGTTTTTACTTAGCTGG

TGATTATACTAAACAAAAGTATTTGGCTTCAATGGAAGGAGCTGTTCTCTCAGGGAAGCTTTGTGCACAG

GCTATTGTACAGGATTATGAGTTTCTTTTTGCTTCAGCGCAAAGAAGGTTGGCACAGGCAAGCATTCATT

GA

>Theobroma cacao

ATGAGTCTCTGTGGGAGTGTTTCTGCTGTGCGCTTGAACTCCCAAAGCAACACAATAAGCATGGGAAGTG

TCTTAGCTTTTAGAGGTGGTGAATCCATGGGACATGCCTTGAGAATTCCCTTTAAAAAGAGGTCAAGTAA

GGGTGCTTGTCCTTTGCAGGTAGTTTGCATAGATTATCCAAGGCCAGAGCTTGAGAATACTGTTAATTTT

TTGGAGGCTGCTTCTCTATCTGCTTCTTTTCGTTCTGCTCCCCGTCCAACTAAGCCATTGAAAGTTATAA

TTGCTGGTGCAGGTTTGGCTGGTTTGTCAACTGCAAAATATTTAGCAGATGCAGGTCACAAACCTCTGTT

GCTTGAAGCAAGAGATGTCCTAGGTGGAAAGGTGGCCGCATGGAAAGATGATGATGGAGATTGGTATGAG

ACAGGCCTACATATATTCTTCGGGGCTTATCCAAATGTGCAAAACCTGTTTGGAGAACTTGGCATTAATG

ACCGGCTGCAATGGAAGGAGCACTCTATGATATTTGCAATGCCAAACAAACCTGGAGAGTTCAGCCGATT

TGATTTTCCAGAAGTTCTACCTGCACCCTTAAATGGGATATGGGCCATTTTGAAGAACAATGAAATGCTG

ACTTGGCCAGAGAAAGTGAAGTTTGCAATAGGACTCCTACCAGCAATGCTTGGCGGACAACCTTATGTTG

AGGCCCAAGATGGTCTAACTGTTAAAGAGTGGATGAGAAAGCAGGGCATACCTGATCGTGTGACTGACAA

TGTGTTTATTGCCATGTCAAAGGCACTGAACTTCATTAACCCAGATGAACTTTCAATGCAGTGTATATTG

ATTGCTTTGAATCGATTTCTGCAGGAGAAAAATGGATCAAAGATGGCATTCTTGGATGGCAACCCCCCTG

AGAGGCTTTGCATGCCTATTGTTAATCATATTGAGTCACTGGGTGGTGAGGTCTGGCTTAACTCACGAAT

AAAGAAAATAGAGCTCAATGATGATGGAACTGTGAAGAGTTTTCTTCTAACTAATGGCAATACAATTGAA

GGAGATGCTTATGTAATGGCAGCTCCAGTTGATATTCTGAAGCTACTTTTGCCTGAAGACTGGAGAGAGA

TTTCATACTTCAAGAAATTAGAGAAATTAGTTGGAGTCCCAGTAATCAACGTTCACATATGGTTTGATAG

GAAATTGAAGAACACCTACAATCATCTACTCTTTAGCAGAAGTCCCCTTCTAAGTGTCTATGCTGACATG

TCTGTAACGTGTAAGGAATATTACAATCCAAACCAGTCCATGTTGGAGTTAGTTTTTGCCCCTGCAGAAG

AATGGATTGCCCGTAGTGACTTGGAAATTATTGATGCTACAATGAAGGAGCTTGCAAAACTCTTTCCTGA

TGAAATTTCTGCAGATCAGAGCAAAGCAAAAGTTATAAAGTACCATATTGTTAAAACACCAAGATCTGTA

TATAAAACTGTTCCAGATTGTGAACCCTGCCGCCCATTGCAAAGATCTCCAATAGAGGGGTTCTATCTAG

CAGGTGATTACACAAAACAAAAGTATCTGGCTTCGATGGAAGGCGCTGTTCTCTCAGGGAAGCTTTGTGC

ACAGTCTATTGTACAGGATTATGAGTTGCTTCTTGCTCTGGGACAAAGAAAGTTGGCAGGAGCAAGCATT

CACTAA

>Abrus precatorius

ATGGCTGCGTGTGGGTGTATATCTGCGGCGAACTTGAATTGGCAGATTGGTGCTAGAAGCATATCCAAAT

TCGGTTCTTCAGATGCCACAATTTCGTTATCATTTGGTGGGAGTGAGTCCATGGGTGTTAGTGTGCGACC

TCGTTCTGCTAAGAGCACCAGGTTGAGGAACCATGCATCACCCTTGAGTGTCGTTTGTGTCGATTATCCA

CGCCCTGAGCTTGAAAACACTGTTAATTTCATCGAAGCTGCTTACTTGTCTTCCACCTTTCGTGCTTCTC

CGCGTCCAGAAAAACCCTTGAATGTCGTTATTGCTGGTGCAGGATTGGCTGGCTTATCAACTGCAAAATA

TTTAGCAGATGCTGGTCATAAACCTATATTGCTGGAGGCAAGAGATGTTCTAGGTGGAAAGGTTGCTGCA

TGGAAAGATGAAGATGGAGACTGGTACGAGACAGGCCTACACATCTTCTTTGGGGCTTACCCTAATGTGC

AGAACTTATTTGGAGAACTTGGTATTAATGATCGGTTACAATGGAAGGAGCATTCTATGATTTTTGCAAT

GCCAAATAAGCCTGGAGAGTTTAGTCGCTTTGATTTTCCTGAAGTTCTTCCCGCCCCATTAAATGGAATA

TGGGCAATATTGAGGAACAATGAGATGCTGACATGGCCAGAGAAAGTCAAATTTGCAATTGGGCTTCTGC

CAGCTATGCTTGGTGGACAGGCGTATGTTGAGGCTCAAGATGGTGTTTCTGTTAAGGAGTGGATGAGAAA

GCAGGGCATACCCGATCGGGTAACTGATGAGGTGTTCATAGCAATGTCAAAGGCACTAAACTTCATCAAT

CCTGATGAACTTTCAATGCAATGTATATTGATTGCTTTAAACCGATTTCTTCAGGAGAAACATGGTTCTA

AGATGGCCTTTTTGGATGGCAATCCCCCTGAAAGACTTTGTATACCAATTGTTGATCATATTCAGTCCTT

GGGGGGTGAAGTTCATCTGAATTCACGCATTCAAAAAATTGAGCTAAATGATGATGGCACAGTGAAGAGC

TTCTTACTAAATAATGGGAGGGTGATGGAAGGGGATGCTTACGTGTTTGCAACTCCAGTGGATATTCTGA

AGCTTCTTCTGCCTGACAACTGGAAAGGAATTCCTTATTTTCAGAGATTGGATAAATTAGTTGGAGTCCC

GGTCATAAATGTTCACATATGGTTTGACAGAAAACTGAAAAACACATATGATCACCTTCTCTTTAGCAGA

AGTCCCCTTCTGAGTGTATATGCTGACATGTCAGTTACTTGCAAGGAATATTATAACCCAAACCAGTCTA

TGTTGGAGTTAGTTTTTGCACCAGCTGAAGAATGGGTGTCACGTAGTGATGAAGATATTATACGTGCCAC

AATGTCTGAACTTGCCAAACTCTTTCCTAATGAAATTTCTGCTGATCAAAGCAAAGCGAAGATTGTCAAG

TACCATGTTGTTAAAACACCAAGGTCGGTTTACAAAACTGTTCCAAATTGTGAACCTTGTCGTCCCGTAC

AAAGATCTCCTGTAGAAGGTTTCTATTTAGCTGGAGATTACACAAAACAAAAATATTTAGCTTCAATGGA

AGGTGCTGTTCTTTCTGGGAAGCTTTGTGCACAGGCAATTGTACAGGATTCTGAGCTACTTGCTGCGCGT

GGTCACAAAAGAATGGCTCAAGCAAGTGTTATTTAA

> *Ricinus communis*

ATGGCTCTATATGGGGGTGTTTCTGCTTTGAATTTAAGCTGGCATAGTGATGTCTTAGACACTAGAAATC

TGCAATCAGCCCTTAGATGTGGTTACGCTACCTGTTCTAATCAAACCAATGTACTAGCTTTTAGAGGCAG

TGAATCTATGGGCCATGCTTTGAGAAATTCTTCTAAAACAAGATTTAGGAATACTGGTAGCTGCCCTTTG

AAGGTAGTTTGTGTGGACTATCCTAGACCAGACCTTGATAACACAGTGAATTTCTTGGAAGCTGCCTACT

TATCATCATCTTTTCGATCTTCTTCCCCTCCAGATAAACCATTGAAGGTTGTAATTGCTGGTGCAGGATT

GGCTGGTTTATCAACTGCAAAATATTTGGCAGATGCAGGACACAAGCCTTTATTGCTGGAAGCAAGAGAT

GTTCTAGGTGGAAAGGTGGCTGCATGGAAAGATGATGATGGGGACTGGTACGAGACAGGCTTGCATATAT

TCTTTGGAGCATACCCAAATGTGCAGAACCTGTTTGGAGAACTTGGTATAAATGATAGATTGCAGTGGAA

GGAGCATTCTATGATATTTGCGATGCCAAACAAGCCTGGAGAATTCAGCCGATTTGATTTCCCAGATGTT

CTTCCTGCACCATTAAATGGGATATGGGCAATTCTGAGAAACAATGAGATGCTGACATGGCCAGAGAAAG

TGAAATTTGCAATTGGACTCCTGCCAGCGATGGTTGGTGGACAGGCCTATGTTGAGGCTCAAGATGGTTT

AAGTGTTCAAGAGTGGATGAGAAAGCAGGGCGTACCTGATAGAGTGACTAAGGAGGTTTTTATTGCTATG

TCAAAGGCGCTAAACTTTATTAACCCTGATGAGCTTTCAATGCAATGTATATTGATAGCATTGAACAGAT

TTCTTCAGGAGAAACATGGTTCAAAGATGGCTTTCTTAGATGGAAATCCCCCAGAGAGACTCTGCATGCC

AATTGTTGACCATGTGCAGTCACTTGGTGGTGAAGTCCGGCTAAATTCACGAATAAAGAAAATTGAATTA

AATAATGATGGAGCAGTGAAGAACTTTTTACTAAATAATGGGGAAGTGATTGAAGGAGATGTTTATGTGG

TTGCTACTCCAGTTGATATCCTGAAGCTTCTTTTGCCTGATAACTGGAAAGAGATTCCATACTTCAAGAA

GCTGGATAAATTAGTTGGAGTTCCTGTTATTAATGTTCACATATGGTTTGACAGGAAGCTGAAGAATACA

TATGATCACCTACTTTTCAGCAGAAGTCCCCTTCTTAGTGTTTATGCGGACATGTCTGTAACATGTAAGG

AATATTATAATCCAAATCAGTCTATGCTGGAGTTAGTTTTTGCACCTGCAGAAGAATGGGTATCACGCAG

CGATGAAGAAATTATTGAGGCTACAATGATGGAACTAGCAAAACTCTTTCCTGATGAAATATCTGCAGAT

CAGAGCAAAGCAAAAATTGTTAAATACCATGTTGTCAAAACTCCCAGGTCTGTTTACAAGACTGTCCCAA

ATTGTGAACCTTGCCGACCCTTGCAAAGATCTCCTATAGAGGGCTTCTATTTGGCTGGTGACTACACAAA

ACAAAAATATTTGGCTTCGATGGAGGGTGCTGTTCTATCCGGGAAGTATTGTGCACAAGCCATTGTACAG

GATTATGGGTTGCTTATCGCTCGCAAGCAAAAAAAGTTGGCTGAGGTAACCGTAATTTAA

>Citrus x paradisi

ATGAGCCTTTGCTTCAGCGTTTCTGAAAGTGCTTTCAACTTGCGATATGGTTTCCGAGATAGTGAACCGA

TGGGTCAGAGCCTGAAAATTCGAGTTAAAACGAGGACAAGGAAGGGTTTCTGTCCTTCGAAGGTGGTTTG

TGTGGACTACCCAAGACCAGATATTGATAATACATCTAATTTCTTGGAAGCTGCTTACTTATCTTCGTCA

TTTCGTACTTCTCCTCGTCCTTCTAAGCCGTTGAAAGTTGTAATTGCTGGTGCAGGTTTGGCTGGTTTAT

CAACTGCAAAATATTTGGCAGATGCAGGCCACAAGCCTTTGTTACTGGAAGCAAGAGATGTTCTAGGTGG

AAAGATAGCTGCCTGGAAAGATGGGGACGGGGACTGGTATGAGACAGGCCTTCATATTTTCTTCGGGGCT

TACCCAAATATACAGAACCTGTTTGGAGAACTTGGTATTAATGACCGGTTGCAGTGGAAGGAGCACTCTA

TGATTTTTGCAATGCCAAACAAGCCCGGAGAATTCAGCCGATTTGATTTTCCTGAAGTTCTTCCAGCTCC

GCTAAATGGGATATTGGCCATTTTAAGGAACAATGAAATGCTGACTTGGCCGGAGAAAGTGAAGTTTGCR

ATTGGACTGCTTCCAGCAATAATTGGTGGACAGGCATATGTTGAAGCTCAAGATGGTTTAACTGTTCAGG

AGTGGATGAGAAAGCAGGGTGTACCTGATCGAGTGACGACGGAGGTGTTTATTGCCATGTCAAAGGCACT

AAACTTCATAAACCCTGATGAACTGTCAATGCAATGTATATTGATTGCCTTAAACCGATTTCTTCAGGAG

AAGCATGGTTCRAAGATGGCATTCTTAGATGGCAACCCCCCAGAGAGACTTTGCTTGCCTATTGTTGAAC

ACATTCAGTCACTGGGTGGTGAAGTCCGGCTTAATTCCCGAGTTCAGAAAATTGAGCTCAATGATGATGG

AACTGTGAAGAATTTTTTACTAACTAATGGCAATGTGATTGACGGAGATGCTTATGTATTTGCCACACCT

GTTGATATCCTCAAGCTTCAGTTACCTGAAAACTGGAAAGAGATGGCATACTTCAAGAGATTAGAGAAAT

TGGTGGGAGTTCCAGTCATCAACATCCACATATGGTTTGACAGGAAATTGAAAAACACTTATGATCACCT

ACTCTTTAGCAGAAGTCCCCTTCTAAGTGTGTATGCCGACATGTCTTTAACTTGTAAGGAGTATTACAAC

CCCAATCAATCCATGCTGGAGTTAGTTTTTGCCCCGGCTGAAGAGTGGATCTCATGCAGTGACTCAGAAA

TCATTGATGCTACAATGAAGGAGCTTGCAAAACTATTTCCTGATGAAATTTCTGCTGATCAGAGCAAAGC

AAAGATTGTGAAGTACCATGTCGTCAAAACGCCAAGGTCTGTATATAAAACCATCCCAAATTGTGAACCT

TGCCGTCCCTTACAAAGGTCTCCTGTAGAAGGGTTTTATTTAGCCGGGGATTACACAAAACAGAAGTATT

TRGCTTCAATGGAAGGTGCTGTTTTGTCAGGGAAGCTTTGTGCACAAGCAATTGTACAGGACTATGTGCT

GCTTGCTGCACGGGGGAAAGGGAGATTGGCTGAGGCAAGCATGTGTCCATAA

>Citrus clementina

ATGAGCCTTTGCTTCAGCGTTTCTGAAAGTGCTTTCAACTTGCGATATGGTTTCCGAGATAGTGAACCGA

TGGGTCAGAGCCTGAAAATTCGAGTTAAAACGGGGACAAGGAAGGGTTTCTGTCCTTCGAAGGTGGTTTG

TGTGGACTACCCAAGACCAGATATTGATAATACATCTAATTTCTTGGAAGCTGCTTACTTGTCTTCGTCA

TTTCGTACTTCTCCTCGTCCTTCTAAGCCGTTGAAAGTTGTAATTGCTGGTGCAGGTTTGGCTGGTTTAT

CAACTGCAAAATATTTGGCAGATGCAGGCCACAAGCCTTTGTTACTGGAAGCAAGAGATGTTCTAGGTGG

AAAGGTAGCTGCCTGGAAAGATGGGGACGGGAACTGGTATGAGACAGGCCTTCATATTTTCTTCGGGGCT

TACCCAAATATACAGAACCTGTTTGGAGAACTTGGTATTAATGATCGGTTGCAGTGGAAGGAGCACTCTA

TGATTTTTGCAATGCCAAACAAGCCCGGAGAATTCAGCCGATTTGATTTTCCTGAAGTTCTTCCGGCTCC

GCTAAATGGGATATTGGCCATTTTAAGGAATAATGAAATGCTGACTTGGCCGGAGAAAGTGAAGTTTGCA

ATTGGACTGCTTCCAGCAATAATTGGCGGACAGGCATATGTTGAAGCTCAAGATGGTTTAACTGTTCAGG

AGTGGATGAGAAAGCAGGGTGTACCTGATCGAGTGACGACAGAGGTGTTTATTGCCATGTCAAAGGCACT

AAACTTCATAAACCCTGATGAACTGTCAATGCAATGTATATTGATTGCCTTAAACCGATTTCTTCAGGAG

AAGCATGGTTCGAAGATGGCATTCTTAGATGGCAACCCCCCAGAGAGACTTTGCTTGCCTATTGTTGAAC

ACATTCAGTCACTGGGTGGTGAAGTCCGGCTTAATTCCCGAGTTCAGAAAATTGAGCTCAATGATGATGG

AACTGTGAAGAATTTTTTACTAACTAATGGCAATGTGATTGACGGAGATGCTTATGTATTTGCCACACCT

GTTGATATCCTCAAGCTTCAGTTACCTGAAAACTGGAAAGAGATGGCATACTTCAAGAGATTAGAGAAAT

TGGTGGGAGTTCCAGTCATCAACATCCACATATGGTTTGACAGGAAATTGAAAAACACTTATGATCACCT

ACTCTTTAGCAGAAGTTCCCTTCTAAGTGTGTATGCCGACATGTCTTTAACTTGTAAGGAGTATTACAAC

CCCAATCAATCCATGCTGGAGTTAGTTTTTGCCCCGGCTGAAGAGTGGATCTCATGCAGTGACTCAGAAA

TCATTGATGCTACAATGAAGGAGCTTGCAAAACTATTTCCTGATGAAATTTCTGCTGATCAGAGCAAAGC

AAAGATTGTGAAGTACCATGTCGTCAAAACGCCAAGGTCTGTATATAAAACCATCCCAAATTGTGAACCT

TGCCGTCCCTTACAAAGGTCTCCTGTAGAAGGGTTTTATTTAGCCGGGGATTACACAAAACAGAAGTATT

TGGCTTCAATGGAAGGTGCTGTTTTGTCAGGGAAGCTTTGTGCACAAGCAATTGTACAGGACTATGTGCT

GCTTGCTGCACGGGGGAAAGGGAGATTGGCTGAGGCAAGCATGTGTCCATAA

>Citrus sinensis

ATGAGCCTTTGCTTCAGCGTTTCTGAAAGTGCTTTCAACTTGCGATATGGTTTCCGAGATAGTGAACCGA

TGGGTCAGAGCCTGAAAATTCGAGTTAAAACGGGGACAAGGAAGGGTTTCTGTCCTTCGAAGGTGGTTTG

TGTGGACTACCCAAGACCAGATATTGATAATACATCTAATTTCTTGGAAGCTGCTTACTTGTCTTCGTCA

TTTCGTACTTCTCCTCGTCCTTCTAAGCCGTTGAAAGTTGTAATTGCTGGTGCAGGTTTGGCTGGTTTAT

CAACTGCAAAATATTTGGCAGATGCAGGCCACAAGCCTTTGTTACTGGAAGCAAGAGATGTTCTAGGTGG

AAAGGTAGCTGCCTGGAAAGATGGGGACGGGAACTGGTATGAGACAGGCCTTCATATTTTCTTCGGGGCT

TACCCAAATATACAGAACCTGTTTGGAGAACTTGGTATTAATGATCGGTTGCAGTGGAAGGAGCACTCTA

TGATTTTTGCAATGCCAAACAAGCCCGGAGAATTCAGCCGATTTGATTTTCCTGAAGTTCTTCCGGCTCC

GCTAAATGGGATATTGGCCATTTTAAGGAATAATGAAATGCTGACTTGGCCGGAGAAAGTGAAGTTTGCA

ATTGGACTGCTTCCAGCAATAATTGGCGGACAGGCATATGTTGAAGCTCAAGATGGTTTAACTGTTCAGG

AGTGGATGAGAAAGCAGGGTGTACCTGATCGAGTGACGACAGAGGTGTTTATTGCCATGTCAAAGGCACT

AAACTTCATAAACCCTGATGAACTGTCAATGCAATGTATATTGATTGCCTTAAACCGATTTCTTCAGGAG

AAGCATGGTTCGAAGATGGCATTCTTAGATGGCAACCCCCCAGAGAGACTTTGCTTGCCTATTGTTGAAC

ACATTCAGTCACTGGGTGGTGAAGTCCGGCTTAATTCCCGAGTTCAGAAAATTGAGCTCAATGATGATGG

AACTGTGAAGAATTTTTTACTAACTAATGGCAATGTGATTGACGGAGATGCTTATGTATTTGCCACACCT

GTTGATATCCTCAAGCTTCAGTTACCTGAAAACTGGAAAGAGATGGCATACTTCAAGAGATTAGAGAAAT

TGGTGGGAGTTCCAGTCATCAACATCCACATATGGTTTGACAGGAAATTGAAAAACACTTATGATCACCT

ACTCTTTAGCAGAAGTTCCCTTCTAAGTGTGTATGCCGACATGTCTTTAACTTGTAAGGAGTATTACAAC

CCCAATCAATCCATGCTGGAGTTAGTTTTTGCCCCGGCTGAAGAGTGGATCTCATGCAGTGACTCAGAAA

TCATTGATGCTACAATGAAGGAGCTTGCAAAACTATTTCCTGATGAAATTTCTGCTGATCAGAGCAAAGC

AAAGATTGTGAAGTACCATGTCGTCAAAACGCCAAGGTCTGTATATAAAACCATCCCAAATTGTGAACCT

TGCCGTCCCTTACAAAGGTCTCCTGTAGAAGGGTTTTATTTAGCCGGGGATTACACAAAACAGAAGTATT

TGGCTTCAATGGAAGGTGCTGTTTTGTCAGGGAAGCTTTGTGCACAAGCAATTGTACAGGACTATGTGCT

GCTTGCTGCACGGGGGAAAGGGAGATTGGCTGAGGCAAGCATGTGTCCATAA

>Citrus maxima

ATGAGCCTTTGCTTCAGCGTTTCTGAAAGTGCTTTCAACTTGCGATATGGTTTCCGAGATAGTGAACCGA

TGGGTCAGAGCCTGAAAATTCGAGTTAAAACGAGGACAAGGAAGGGTTTCTGTCCTTCGAAGGTGGTTTG

TGTGGACTACCCAAGACCAGATATTGATAATACATCTAATTTCTTGGAAGCTGCTTACTTATCTTCGTCA

TTTCGTACTTCTCCTCGTCCTTCTAAGCCGTTGAAAGTTGTAATTGCTGGTGCAGGTTTGGCTGGTTTAT

CAACTGCAAAATATTTGGCAGATGCAGGCCACAAGCCTTTGTTACTGGAAGCAAGAGATGTTCTAGGTGG

AAAGATAGCTGCCTGGAAAGATGGGGACGGGGACTGGTATGAGACAGGCCTTCATATTTTCTTCGGGGCT

TACCCAAATATACAGAACCTGTTTGGAGAACTTGGTATTAATGACCGGTTGCAGTGGAAGGAGCACTCTA

TGATTTTTGTAATGCCAAACAAGCCCGGAGAATTCAGCCGATTTGATTTTCCTGAAGTTCTTCCAGCTCC

GCTAAATGGGATATTGGCCATTTTAAGGAACAATGAAATGCTGACTTGGCCGGAGAAAGTGAAGTTTGCA

ATTGGACTGCTTCCAGCAATAATTGGTGGACAGGCATATGTTGAAGCTCAAGATGGTTTAACTGTTCAGG

AGTGGATGAGAAAGCAGGGTGTACCTGATCGAGTGACGACGGAGGTGTTTATTGCCATGTCAAAGGCACT

AAACTTCATAAACCCTGATGAACTGTCAATGCAATGTATATTGATTGCCTTAAACCGATTTCTTCAGGAG

AAGCATGGTTCGAAGATGGCATTCTTAGATGGCAACCCCCCAGAGAGACTTTGCTTGCCTATTGTTGAAC

ACATTCAGTCACTGGGTGGTGAAGTCCGGCTTAATTCCCGAGTTCAGAAAATTGAGCTCAATGATGATGG

AACTGTGAAGAATTTTTTACTAACTAATGGCAATGTGATTGACGGAGATGCTTATGTATTTGCCACACCT

GTTGATATCCTCAAGCTTCAGTTACCTGAAAACTGGAAAGAGATGGTATACTTCAAGAGATTAGAGAAAT

TGGTGGGAGTTCCAGTCATCAACATCCACATATGGTTTGACAGGAAATTGAAAAACACTTATGATCACCT

ACTCTTTAGCAGAAGTCCCCTTCTAAGTGTGTATGCCGACATGTCTTTAACTTGTAAGGAGTATTACAAC

CCCAATCAATCCATGCTGGAGTTAGTTTTTGCCCCGGCTGAAGAGTGGATCTCATGCAGTGACTCAGAAA

TCATTGATGCTACAATGAAGGAGCTTGCAAAACTATTTCCTGATGAAATTTCTGCTGATCAGAGCAAAGC

AAAGATTGTGAAGTACCATGTCGTCAAAACGCCAAGGTCTGTATATAAAACCATCCCAAATTGTGAACCT

TGCCGTCCCTTACAAAGGTCTCCTGTAGAAGGGTTTTATTTAGCCGGGGATTACACAAAACAGAAGTATT

TGGCTTCAATGGAAGGTGCTGTTTTGTCAGGGAAGCTTTGTGCACAAGCAATTGTACAGGACTATGTGCT

GCTTGCTGCACGGGGGAAAGGGAGATTGGCTGAGGCAAGCATGTGTTAA

>Diospyros kaki

ATGTCTCAATTCGGACATGTTTCTGCCCTCAACCTGAGTGGGCAAAGCAATCTAATAAACTTTTGGAACC

CACAATCCACTTGGATATGTGGTTCAAGGCAAACCAATGTACTATCATTTGGAGGGACTGATTCCGTGGG

TTATGGGTTGAGAATTCCTAATGCAAATGCTATTAGAACAAGACCGAAGAAGGGCGTGTGCCCCTTGCAG

GTCGTCTGCATTGACTATCCAAGACCAGATCTTGACAGCACTTCCAATTTTTTGGAAGCAGCTTATTTGT

CTTCATTCTTCCGTACGGCTCCCCGACCAGATAAGCCGCTGAAGGTTGTAATTGCGGGTGCAGGTTTGGC

TGGATTATCAACTGCAAAATATTTGGCAGATGCAGGTCATAAACCTTTATTATTGGAAGCGAGGAATGTT

TTAGGTGGAAAGGTGGCTGCTTGGAAAGATGAGGATGGAGACTGGTATGAGACTGGATTACATATATTTT

TTGGGGCTTACCCAAATGTACAGAACCTGTTTGGAGAGCTTGGTATAAATGATAGGTTGCAGTGGAAAGA

ACATTCTATGATATTTGCAATGCCAAATAAGCCAGGGGAGTTCAGCCGATTTGACTTCGCTGAAGTTCTA

CCAGCACCATTAAATGGGATTTGGGCCATCTTAAAGAATAATGAAATGCTTACTTGGCCTGAGAAAGTCA

AGTTTGCAATTGGACTGTTGCCAGCAATGATAGGTGGGCAGCCCTATGTCGAAGCTCAAGATGGTTTAAC

TGTTAAAGACTGGATGAGGAAACAAGGTGTACCAGATCGAGTGACCACTGAGGTGTTCATTGCCATGTCT

AAAGCATTAAACTTCATAAACCCTGATGAACTTTCAATGCAGTGTATTTTGATTGCTTTGAACCGGTTTC

TTCAGGAGAAACATGGTTCAAAGATGGCATTCTTGGATGGTAATCCCCCTGAGAGACTTTGCCAGCCAAT

TGTGGATCACATTCAATCACTGGGAGGTGAAGTCCAACTTAATGCTCGAATTCAAAAAATTGAGTTGAAT

GAAGATGGAACTGTGAAGAGCTTTTTACTAAATAATGGTAATGTCATCAGTGGAGATGCTTATGTGTTTG

CAACTCCAGTTGATATCTTGAAGCTTCTTTTGCCGGATGACTGGAAAGGGGTTCCCTACTTCAAAAAATT

AGATAAACTAGTTGGAGTTCCTGTTATAAACGTTCACATATGGTTTGACAGGAAGCTGAGGAACACATAT

GATCATTTACTTTTTAGCAGAAGTCCCCTTCTCAGTGTATATGCTGACATGTCGGTAACATGTAAGGAAT

ATTACAATCCAAATCAGTCTATGCTAGAATTGGTTTTTGCACCAGCAGAGGAATGGATTTCGCGGAGTGA

CACAGAAATTATTGATGCTACTATGAAGGAACTTGCAAAACTCTTCCCTGATGAAATTTGTCCAGATCAG

AGCAAAGCAAAAATTTTGAAGTATCATGTTGTTAAAACACCGAGATCTGTGTATAAAACTGTCCCAAACT

GTGAACCATGCCGTCCCTTGCAAAGGTCCCCTATAGAAGGATTCTATTTAGCCGGTGACTACACAAAACA

AAAATATTTGGCTTCAATGGAAGGTGCTGTCCTATCAGGAAAGCTTTGTGCCCAAGCTGTTGTACAGGAT

TACGAGTTTCTTGCAGCCCAGGGGCAGAGAAAGCTGGTGGAAGCAAGTATGGTGTAA

>Rhododendron kiusianum

ATGTCTCAATTTGGACATGCTTCTGCTGTATATTGGACTGGGCAACACAACGCAACTAATTTGTGGAACC

CAAGGTATACTTGGAGATGCGGTTGTCCCATTAGTTCAAGGCATAACAATGCGCTATCATTTAAAGGGAG

TGATTCGTTGGGTCATAGGGTATCAAATGCCTATACTATTAGAACCAGACCAATGAAGAATGTGCAGCCT

TTGCAGGTGGTTTGCATGGACTATCCCAGACCAGAGCTTGAGAGTACTGTCAATTATTTGGAAGCTGCTT

ACTTATCTTCATCCTTTCGTACTTCTCCTCGTCCAGATAAACCATTAAAGGTCGTAATTGCTGGTGCAGG

TTTGGCTGGTTTGTCAACTGCAAAATATTTGGCAGATGCAGGCCATAAACCCATATTGTTGGAAGCAAGA

GATGTTTTAGGTGGAAAGGTGGCTGCGTGGAAAGATGATGATGGAGACTGGTATGAGACTGGCTTACATA

TATTCTTTGGCGCCTACCCAAATGTCCAGAACCTGTTTGGAGAACTTGGTCTAAATGATCGGTTGCAGTG

GAAAGAACATTCTATGATATTTGCAATGCCAAACAAGCCAGGGGAGTTCAGTCGATTTGACTTCCTTGAC

ATTCTACCAGCACCACTGAACGGGATATGGGCTATCTTAAAGAACAATGAAATGCTTACTTGGCCAGAGA

AAATAAAGTTTGCAATTGGACTACTGCCGGCAATGGTCGGTGGACAGGCTTATGTTGAAGCTCAAGATGG

TTTAACTGTGAAAGACTGGATGAAGAAACAAGGTGTACCAGATCGAGTAACTACTGAGGTGTTTATTGCC

ATGTCAAAGGCATTAAACTTCATAAACCCTGATGAACTTTCCATGCAGTGTATTTTGATTGCCTTAAACC

GGTTTCTTCAGGAAAAGCATGGTTCGAAAATGGCATTTTTGGATGGTAATCCCCCAGAGAGACTTTGCCT

GCCAATTGTCGATCACATTCGGTCACTAGGCGGTGAAGTCCGACTTAATTCTCGAATTCAAAAGATTGAG

CTGAATAAAGACGGAACTGTGAAGAACTTTTTGCTAAAGAACGGTAATGTTATTGAAGGAGATGTTTACG

TTTTTGCCACTCCAGTCGATATCTTGAAGCGTCTTTTGCCCGAAGACTGGAAAGAGGTTCCTTACTTCAG

GAAATTGGAGAAATTAGTTGGAGTTCCCGTCATAAATGTTCACATATGGTTCGACAGGAAACTGAGGAAC

ACATACGATCATCTACTTTTTAGCAGAAGTCACCTTCTCAGTGTGTATGCTGACATGTCTGTTACATGCA

AGGAATATTACGACCCGCATCGCTCTATGCTGGAATTGGTTTTTGCCCCTGCAGAGGAATGGATCTCAAA

AAGTGATCAAGAAATTATTGACGCTACTATGAAGGAGCTCGCAAAACTCTTTCCTGATGAAATTTCTGCA

GATCAGAGTAAAGCAAAAATATTGAAGTACCATGTCGCTAAAACACCAAGGTCTGTTTATAAAACTGTCC

CAGACTGTGAACCTTGCCGTCCATTACAGAGATCCCCAGTGGAAGGTTTCTATTTGGCAGGTGACTACAC

AAAACAAAAATATTTGGCTTCAATGGAAGGTGCTGTTCTTTCAGGAAAGTTTTGTGCACAAGCTATTGTA

CAGGATTACGAATTGCTTGCTTCCCGGAGCCAGAAAAAACTAGCTGAGGCAAGTCTGGTGTAA

>Soybean

ATGGCCGCTTGTGGCTATATATCTGCTGCCAACTTCAATTATCTCGTTGGCGCCAGAAACATATCCAAAT

TCGCTTCTTCAGACGCCACAATTTCGTTTTCATTTGGCGGGAGCGACTCAATGGGTCTTACTTTGCGACC

CGCTCCGATTCGTGCTCCTAAGAGGAACCATTTCTCTCCCTTGCGTGTCGTTTGCGTCGATTATCCACGC

CCAGAGCTCGAAAACACCGTTAATTTCGTTGAAGCTGCTTACTTGTCTTCCACCTTTCGTGCTTCTCCGC

GTCCTCTAAAACCCTTGAACATCGTTATTGCCGGTGCAGGATTGGCTGGTTTATCAACTGCAAAATATTT

GGCTGATGCTGGGCATAAACCTATATTGCTGGAAGCAAGAGACGTTCTAGGTGGAAAGGTTGCTGCATGG

AAAGACAAGGATGGAGACTGGTACGAGACAGGCCTACACATCTTTTTTGGGGCTTACCCTTATGTGCAGA

ACCTTTTTGGAGAACTTGGCATTAATGATCGGTTACAATGGAAAGAGCATTCTATGATTTTTGCTATGCC

AAATAAGCCTGGAGAGTTTAGTCGATTTGATTTTCCTGAAGTTCTTCCCTCCCCATTGAATGGAATATGG

GCAATATTGAGGAACAATGAGATGCTTACATGGCCAGAGAAAGTAAAATTTGCAATTGGGCTTCTCCCAG

CTATGCTTGGCGGACAGCCATATGTTGAGGCTCAAGATGGTCTTTCTGTTCAAGAATGGATGAAAAAGCA

GGGCGTACCTGAACGGGTAGCTGATGAGGTGTTCATAGCAATGTCAAAGGCACTAAACTTCATCAATCCT

GATGAACTTTCAATGCAATGTATATTGATTGCTTTAAACCGATTTCTTCAGGAGAAACATGGTTCTAAGA

TGGCCTTTTTGGATGGCAATCCACCCGAAAGACTTTGTATGCCAATAGTTGATTATATTCAGTCCTTGGG

TGGTGAAGTTCATCTAAATTCGCGCATTCAAAAAATTGAGCTAAATGATGATGGAACGGTGAAGAGCTTC

TTACTAAATAATGGGAAAGTGATGGAAGGGGATGCTTATGTGTTTGCAACTCCAGTGGATATTCTGAAGC

TTCTTCTACCAGATAACTGGAAAGGGATTCCATATTTCCAGAGATTGGATAAATTAGTTGGCGTCCCAGT

CATAAATGTTCACATATGGTTTGACAGAAAACTGAAGAACACATATGATCACCTTCTCTTTAGCAGAAGT

CCCCTTCTGAGTGTATATGCTGACATGTCAGTAACTTGCAAGGAATATTATAGCCCAAACCAGTCAATGT

TAGAGTTGGTTTTTGCACCAGCCGAAGAATGGATTTCACGTAGTGATGATGATATTATTCAAGCCACGAT

GACTGAGCTTGCCAAACTCTTTCCTGATGAAATTTCTGCAGACCAAAGCAAAGCAAAGATTCTCAAGTAC

CATGTTGTTAAAACACCAAGGTCGGTTTACAAAACTGTTCCAAATTGTGAACCTTGTCGACCCATTCAAA

GATCTCCTATAGAAGGTTTCTATTTAGCTGGAGATTACACAAAACAAAAATATTTAGCTTCAATGGAAGG

CGCTGTTCTTTCTGGGAAGCTTTGTGCACAGGCTATTGTACAGGATTCTGAGCTACTAGCTACTCGGGGC

CAGAAAAGAATGGCTAAAGCAAGTGTTGTGTAA

>Glycine soja

ATGGCCGCTTGTGGCTATATATCTGCTGCCAACTTCAATTATCTCGTTGGCGCCAGAAACATATCCAAAT

TCGCTTCTTCAGACGCCACAATTTCGTTTTCATTTGGCGGGAGCGACTCAATGGGTCTTACTTTGCGACC

CGCTCCGATTCGTGCTCCTAAGAGGAACCATTTCTCTCCCTTGCGTGTCGTTTGCGTCGATTATCCACGC

CCGGAGCTCGAAAACACCGTTAATTTCGTTGAAGCTGCTTACTTGTCTTCCACCTTTCGTGCTTCTCCGC

GTCCTCTAAAACCCTTGAACATCGTTATTGCCGGTGCAGGATTGGCTGGTTTATCAACTGCAAAATATTT

GGCTGATGCTGGGCATAAACCTATATTGCTGGAAGCAAGAGACGTTCTAGGTGGAAAGGTTGCTGCATGG

AAAGACAAGGATGGAGACTGGTACGAGACAGGCCTACACATCTTTTTTGGGGCTTACCCTAATGTGCAGA

ACCTTTTTGGAGAACTTGGCATTAATGATCGGTTACAATGGAAAGAGCATTCTATGATTTTTGCTATGCC

AAATAAGCCTGGAGAGTTTAGTCGATTTGATTTTCCTGAAGTTCTTCCCTCCCCATTGAATGGAATATGG

GCAATATTGAGGAACAATGAGATGCTTACATGGCCAGAGAAAGTAAAATTTGCAATTGGGCTTCTCCCAG

CTATGCTTGGCGGACAGCCATATGTTGAGGCTCAAGATGGTCTTTCTGTTCAAGAATGGATGAAAAAGCA

GGGCGTACCTGAACGGGTAACTGATGAGGTGTTCATAGCAATGTCTAAGGCACTAAACTTCATCAATCCT

GATGAACTTTCAATGCAATGTATATTGATTGCTTTAAACCGATTTCTTCAGGAGAAACATGGTTCTAAGA

TGGCCTTTTTGGATGGCAATCCACCCGAAAGACTTTGTATGCCGATAGTTGATCATATTCAGTCCTTGGG

TGGTGAAGTTCATCTAAATTCGCGCATTCAAAAAATTGAGCTAAATGATGATGGAACGGTGAAGAGCTTC

TTACTAAATAATGGGAAAGTGATGGAAGGGGATGCTTATGTGTTTGCAACTCCAGTGGATATTCTGAAGC

TTCTTCTACCAGATAACTGGAAAGGGATTCCATATTTCCAGAGATTGGATAAATTAGTTGGCGTCCCAGT

CATAAATGTTCACATATGGTTTGACAGAAAACTGAAGAACACATATGATCACCTTCTCTTTAGCAGAAGT

CCCCTTCTGAGTGTATATGCTGACATGTCAGTAACTTGCAAGGAATATTATAGCCCAAACCAGTCAATGT

TAGAGTTGGTTTTTGCACCAGCCGAAGAATGGATTTCACGTAGTGACGATGATATTATTCAAGCCACGAT

GACTGAGCTTGCCAAACTCTTTCCTGATGAAATTTCTGCAGACCAAAGCAAAGCTAAGATTCTCAAGTAC

CATGTTGTTAAAACACCAAGGTCGGTTTACAAAACTGTTCCAAATTGTGAACCTTGTCGACCCATTCAAA

GATCTCCTATAGAAGGTTTCTATTTAGCTGGAGATTACACAAAACAAAAATATTTAGCTTCAATGGAAGG

CGCTGTTCTTTCTGGGAAGCTTTGTGCACAGGCTATTGTACAGGATTCTGAGCTACTAGCTACTCGGGGC

CAGAAAAGAATGGCTAAAGCAAGTGTTGTGTAA

>Glycine max

ATGGCCGCTTGTGGCTATATATCTGCTGCCAACTTCAATTATCTCGTTGGCGCCAGAAACATATCCAAAT

TCGCTTCTTCAGACGCCACAATTTCGTTTTCATTTGGCGGGAGCGACTCAATGGGTCTTACTTTGCGACC

CGCTCCGATTCGTGCTCCTAAGAGGAACCATTTCTCTCCCTTGCGTGTCGTTTGCGTCGATTATCCACGC

CCGGAGCTCGAAAACACCGTTAATTTCGTTGAAGCTGCTTACTTGTCTTCCACCTTTCGTGCTTCTCCGC

GTCCTCTAAAACCCTTGAACATCGTTATTGCCGGTGCAGGATTGGCTGGTTTATCAACTGCAAAATATTT

GGCTGATGCTGGGCATAAACCTATATTGCTGGAAGCAAGAGACGTTCTAGGTGGAAAGGTTGCTGCATGG

AAAGACAAGGATGGAGACTGGTACGAGACAGGCCTACACATCTTTTTTGGGGCTTACCCTAATGTGCAGA

ACCTTTTTGGAGAACTTGGCATTAATGATCGGTTACAATGGAAAGAGCATTCTATGATTTTTGCTATGCC

AAATAAGCCTGGAGAGTTTAGTCGATTTGATTTTCCTGAAGTTCTTCCCTCCCCATTGAATGGAATATGG

GCAATATTGAGGAACAATGAGATGCTTACATGGCCAGAGAAAGTAAAATTTGCAATTGGGCTTCTCCCAG

CTATGCTTGGCGGACAGCCATATGTTGAGGCTCAAGATGGTCTTTCTGTTCAAGAATGGATGAAAAAGCA

GGGCGTACCTGAACGGGTAACTGATGAGGTGTTCATAGCAATGTCTAAGGCACTAAACTTCATCAATCCT

GATGAACTTTCAATGCAATGTATATTGATTGCTTTAAACCGATTTCTTCAGGAGAAACATGGTTCTAAGA

TGGCCTTTTTGGATGGCAATCCACCCGAAAGACTTTGTATGCCGATAGTTGATCATATTCAGTCCTTGGG

TGGTGAAGTTCATCTAAATTCGCGCATTCAAAAAATTGAGCTAAATGATGATGGAACGGTGAAGAGCTTC

TTACTAAATAATGGGAAAGTGATGGAAGGGGATGCTTATGTGTTTGCAACTCCAGTGGATATTCTGAAGC

TTCTTCTACCAGATAACTGGAAAGGGATTCCATATTTCCAGAGATTGGATAAATTAGTTGGCGTCCCAGT

CATAAATGTTCACATATGGTTTGACAGAAAACTGAAGAACACATATGATCACCTTCTCTTTAGCAGAAGT

CCCCTTCTGAGTGTATATGCTGACATGTCAGTAACTTGCAAGGAATATTATAGCCCAAACCAGTCAATGT

TAGAGTTGGTTTTTGCACCAGCCGAAGAATGGATTTCACGTAGTGACGATGATATTATTCAAGCCACGAT

GACTGAGCTTGCCAAACTCTTTCCTGATGAAATTTCTGCAGACCAAAGCAAAGCTAAGATTCTCAAGTAC

CATGTTGTTAAAACACCAAGGTCGGTTTACAAAACTGTTCCAAATTGTGAACCTTGTCGACCCATTCAAA

GATCTCCTATAGAAGGTTTCTATTTAGCTGGAGATTACACAAAACAAAAATATTTAGCTTCAATGGAAGG

CGCTGTTCTTTCTGGGAAGCTTTGTGCACAGGCTATTGTACAGGATTCTGAGCTACTAGCTACTCGGGGC

CAGAAAAGAATGGCTAAAGCAAGTGTTGTGTAA

>Hibiscus syriacus

ATGAGTCTCTGTGGGAGTGTTTCTGCCTTAAACTTAAACTTCAAAAGCAACAAGATAAGCATGGGAAGAG

TCTTAGCTTTTAGAAGTGGTGAATCCATGGGAAACACCTTGAGAATTCCATTAAAAAAGAGGTCATGTAA

GGGTGCTTGTCCTTTGCAGGTAGTTTGCATAGATTATCCAAGGCCAGAGCTTGAGAATACTGTTAATTTT

CTGGAGGCTGCCTCTCTATCCGCTTATTTACGTTCTGCTCCTCGTCCAACTAAGCCATTGAAAGTCATAG

TTGCGGGTGCAGGTTTGGCTGGTTTGTCAACGGCAAAGTATCTAGCGGATGCAGGTCATAAACCAATATT

ATTGGAAGCAAGAGATGTTCTTGGCGGAAAGGTGGCTGCATGGAAAGATGATGATGGAGATTGGTATGAG

ACAGGCCTACATATATTCTTCGGGGCGTACCCGAATGTGCAAAACTTGTTTGGAGAACTTGGCATCAATG

ATCGGCTGCAATGGAAGGAGCATTCTATGATATTTGCAATGCCAAATAAACCTGGAGAGTTCAGTCGATT

TGATTTTCCAGAAGTTCTACCTGCACCCTTAAATGGGATATGGGCCATTTTGAAGAACAATGAAATGCTG

ACTTGGCCAGAGAAAGTGAAATTTGCAATAGGACTTCTACCTGCAATGCTTGGTGGACAACCTTATGTTG

AGGCCCAAGATGGTTTATCTGTTAAAGAGTGGATGAGAAAGCAGGGTGTACCTGATCGTGTCACTGAAGA

GGTGTTTATTGCCATGTCAAAGGCTCTGAACTTCATTAACCCTGACGAACTTTCAATGCAGTGTATTCTG

ATTGCTTTGAATCGATTTCTTCAGGAAAAGCATGGATCAAAGATGGCTTTCTTGGATGGCAACCCTCCGG

AGAGGCTTTGCATGCCAATCGTCAATCATATTGAATCACTGGGTGGTGAAGTTCGGCTTAACTCACGGTT

AAAGAAAATAGAGCTCAATGCTGATGGAACTGTGAAAAGTTTTCTTCTAAATAATGGCAATATAATTGAA

GGAGATGCTTATGTAGTTGCAACTCCAGTTGATATCTTCAAGTTACTTTTGCCTGAAGACTGGAGAGAAA

TTTCATACTTCAAGAAATTAGATAAATTAGTTGGAGTTCCAGTTATCAACGTTCACATCTGGTTTGATAG

GAAATTGAAGAACACCTATGATCATCTACTGTTCAGCAGAAGTTCGCTTCTAAGTGTTTATGCTGACATG

TCTGTAACGTGTAAGGAATATTACAATCCGAACCAATCCATGTTAGAGTTAGTTTTTGCCCCGGCAGAAG

AATGGATTGCACAAAGTGATTCGGAAATTATTGATGCTACAATGAAGGAGCTTGCAAAGCTCTTCCCTGA

TGAAATATCTGCAGATCAGAGTAAAGCAAAAGTTGTAAAGTACCATATCGTTAAAACACCAAGATCAGTA

TATAAAACTGTTCCAAATTGTGAACCCTGCCGTCCCGTGCAAAGATCTCCGATACAGGGGTTCTATCTAG

CAGGTGATTACACAAAGCAAAAGTATTTAGCTTCCATGGAAGGAGCTGTCCTCTCAGGGAAGTTTTGTGC

ACAGTCTATTGTACAGGATTATGAGTTGCTTCAAACCCTGGGACAAAGAAAGTTGACCGAAGCAAGCATT

CACTGA

>Arachis hypogaea

ATGGCCACGTGTATATCTGCTGTGAACTTGAATTACCAAATTGCCCCAAGAACCGTTTCGAAATTCAGTT

CTGCGACGAGCTCGGACCAAACGGCGTCGTTTTCGCTTGGCGCGAGCGAGTCGATGGGACCGAGTCTCAG

ACTCGCTTTGACTCGTGCTGCTAAGAGCACCACCACCACCACTAGGTTGTTGAGGAAGAAGCATGGC

TCTCTTCCGTTGCGAGTGTTTTGCGTCGATTACCCTCGGCCGGAGCTTGAGAACACCGTGAATTTCCTCG

AAGCAGCGTTCTTGTCTTCGACTTTTCGTGATTCACCACGACCAGCGAAACCGTTGAAGGTCGTTGTTGC

TGGAGCAGGATTGGCTGGTTTATCGACTGCAAAATATTTGGCAGATGCTGGTCACAAGCCTGTATTACTG

GAGGCAAGAGATGTTCTAGGTGGAAAGGTTGCTGCATGGCAAGATGAAGATGGAGACTGGTATGAGACAG

GCCTGCATATATTCTTTGGGGCATACCCTAATGTGCAGAATCTATTTGGAGAACTTGGTATTAATGATCG

GTTACAATGGAAGGAACATTCTATGATTTTTGCAATGCCAAGTAAACCTGGAGAATTTAGTCGATTTGAT

TTTCCTGAAGCCCTACCAGCTCCACTAAATGGAATATTGGCAATATTGAGGAACAATGAGATGCTTACTT

GGCCAGAAAAAGTCAAGTTTGCAATTGGGCTTCTGCCAGCTATGCTTGGTGGTCAGTCTTATGTTGAAGC

TCAAGATGGCCTTTCTGTTAAAGATTGGATGAGAAAGCAGGGTGTACCTGATCGAGTAACTGATGAGGTG

TTCATAGCCATGTCAAAGGCACTAAATTTCATCAACCCGGATGAACTTTCAATGCAATGTATATTGATTG

CCTTAAACCGATTTCTTCAGGAAAAGCATGGTTCTAAAATGGCCTTTTTGGATGGTAATCCCCCTGAAAG

ACTTTGTATGCCTATTGTTGATCATATTCAATCCTTGGGTGGTGAAGTTCATCTAAATTCTCGCATTCAA

AAGATCGATCTAAATGATGATGGCACTGTGAAGAGCTTCTTGCTAAATAATGGGAAGGTGATTGAAGGGG

ATGCTTATGTGTTTGCAACTCCAGTTGATATTCTGAAGCTTCTTGTGCCTAACAATTGGAAAGAGATTCC

ATATTTCCAAAGATTGGAGAAACTAGTAGGAGTCCCGGTTATAAATGTTCATATATGGTTTGACAGAAAA

CTGAAGAACACATATGATCATCTTCTCTTCAGCAGAAGTCCACTTTTGAGTGTATATGCTGACATGTCAG

TAACTTGTAAGGAATATTATAACCCAAACCAGTCTATGTTGGAGTTGGTTTTTGCACCTGCTGAAGAATG

GGTTTCTCGAAGTGATGAAGACATCATTGCTGCTACGATGTCTGAACTTGCCAAACTGTTCCCTGATGAA

ATTTGTGCAGACCAGACAAAAGCAAAGATTGTTAAGTACCATGTTGTTAAAACACCCAGGTCGGTTTACA

AAACTGTTCCAAATTGTGAACCTTGTCGTCCCATACAACGATCTCCTATAGAAGGTTTCTATTTAGCTGG

AGATTACACAAAACAAAAATATTTAGCTTCAATGGAAGGTGCTGTTCTGTCAGGGAAGCTATGTGCACAG

GCTATTGTACAGGATTCTGAGCTACTTGCTGCTCGGAGCCAGAAAGCTGTGGCCCAAGCAAGTGTTATTT

AA

>Castaneasativa

GACAGGAAATCAAACTCTGAGATGTGGGTTTCTTAATAATTCGGTGAAAACCAATGCATTAGCATTTGGA

GGTTGTGAATCCATGGGTCATATTTtGAGAATTCCACATACAAAGGCTATTAGATTGAGGCCGAGGAAGG

GTGTCTCTCCTTTGCAGGTAGTATGTATGGACTTTCCAAGACCAGAGCTTGAGAATACTGTTAATTTCTT

AGAGGCTGCTTATTTGTCTTCTTCCCTCCGTGCATCTGCTCGTCCATCTAAaCCCCTAACAGTTGTAATT

GCTGGTGCAGGTTTGGCTGGTTTGTCTACTGCAAAGTATTTGGCAGATGCTGGTCACAAACCTATACTAT

TGGAGTCAAGAGATGTGCTAggaGGAaaGGTGGCTGCATGGAAAGATGACGATGGAGActGGTATGAGAC

TGGATTACATATATTCTTTGGGGCTTACCCAAATGTGCAGAATCTTTTtGGAGAACTTGGTATTGATGAT

CGGTTGCAATGGAaGGAACATTCTATGATTTTtGCAATGCCAAATAAGCCGGGAGAGTTCAGCCGATTTG

ATTTTCCTGAAGTTCTTCCTGCACCATTAAATGGAATATGGGCTATTTTGAAGAACAATGAGATGCTGAC

TTGGCCAGATAAAGTCAAGTTTGCAATTGGACTCTTGCCAGCAATGCTTGGTGGACAGGCTTATGTTGAA

GCACAAGATGGTTTAACTGTTAAAGAGTGGATGAGAAAGCAGGGAGTACCTGATCGTGTAACTGATGAGG

TGTTTGTAGCCATGTCAAAGGCGCTAAACTTCATTAACCCTGATGAACTTTCAATGCAATGCATATTGAT

TGCTTTGAATAGGTTTCTTCAGGAGAAGAATGGTTCCAAGATGGCTTTCTTGGATGGTAATCCCCCAGAG

AGACTCTGTATGCCAATTGTTGATCATATTCAATCACTCGGcGGTGAAGTAAGACTGAATTCGAGAATAC

AAAAAATCGAGCTAAATAATGATGGAACAGTGAAAAGCTTTTTACtGAATAATGGGAACATGATTGAAGG

AGATGCTTATGTATTtgCTaCTCCAGTTGATATCCTGAAGCTTCTTTTGCCGGAAAACTGGAAAGAGATT

CCATATTTtCAGAGATTAAAGAAATTAGTTGGAGTTCCAGTTATTAATGTCCACATaTGGTTTGACAGAA

AACTGAAGAACACCTATGATCACCTACTGTTTAGCAGAAGTCcACTTCTCAGTGTGTATGCTGACATGTC

ACTAACgTGTAAGGAATATTACAACCCAAACcAATCTATGCTGGAGTTGGTTTTTGCgCCTGCAGAAGAA

TGGATTTCATGCAGTGACTCAGACATTATTGACGCTACAATGAATGAACTTGCAAGACTCTTTCCCGATG

AAATTTCCACGgATCAAAGCAAAGCAAAGATTGTGAAGTACCATGTTGTTAAAACACCAAGGTCTGTTTACAAAATTGT
CCCAGACTGTGAACCTTGC

>Arabidopsis thaliana

ATGGTTGTGTTTGGGAATGTTTCTGCGGCGAATTTGCCTTATCAAAACGGGTTTTTGGAGGCACTTTCTT

CATCTGGAGGTATTGAATTGATGGGACACAGCTTTAGGGTTCCGACTTTTCAAGCACCTAAGACAAGAAC

AAGGAGGAGAAGTACTGCTGGTCCTTTGCAGGTAGTTTGTGTGGATATTCCAAGGCCAGAGCTAGAGAAC

ACTGTCAATTTCTTGGAAGCTGCTAGTTTATCTGCATCTTTCCGTAGTGCTCCTCGTCCTGCAAAGCCTT

TAAAAGTTGTAATTGCTGGTGCTGGATTGGCTGGATTGTCAACTGCAAAGTACCTGGCTGATGCCGGCCA

CAAACCTCTGTTGCTTGAAGCAAGAGATGTTCTTGGTGGAAAGATAGCTGCATGGAAGGATGAAGATGGG

GACTGGTATGAAACTGGTTTACATATATTCTTCGGTGCTTATCCAAATGTGCAGAACTTATTTGGAGAAC

TTGGGATCAATGATCGGTTGCAGTGGAAGGAACACTCCATGATTTTCGCTATGCCAAGTAAACCTGGAGA

GTTTAGTAGATTTGACTTCCCAGATGTCCTACCAGCACCCTTAAACGGTATTTGGGCTATTTTGCGGAAC

AACGAGATGCTGACATGGCCAGAGAAAATAAAGTTTGCTATTGGACTTTTGCCAGCCATGGTCGGCGGCC

AGGCTTATGTTGAAGCCCAAGATGGCTTATCAGTCAAAGAATGGATGGAAAAGCAGGGAGTACCTGAGCG

TGTGACTGACGAGGTGTTTATTGCCATGTCAAAGGCACTAAACTTTATCAACCCTGATGAACTGTCAATG

CAATGCATTTTGATAGCTTTGAACCGGTTTCTTCAGGAAAAACATGGTTCCAAGATGGCATTCTTGGATG

GTAATCCTCCGGAAAGGCTTTGTATGCCAATAGTGGATCATATTCGATCACTAGGTGGGGAAGTGCAACT

TAATTCTAGGATAAAGAAAATTGAGCTCAATAACGATGGCACGGTTAAGAGTTTCTTACTAACTAATGGA

AGCACTGTCGAAGGAGACGCTTATGTGTTTGCCGCTCCAGTCGATATCCTGAAGCTCCTTTTACCAGATC

CCTGGAAAGAAATACCGTACTTCAAGAAATTGGATAAATTAGTTGGAGTGCCAGTTATTAATGTTCATAT

ATGGTTTGATCGAAAACTGAAGAACACATATGATCACCTACTCTTTAGCAGAAGTAACCTTCTTAGCGTG

TATGCGGACATGTCCTTAACTTGTAAGGAATATTACGATCCTAACCGGTCGATGCTGGAGCTAGTATTTG

CACCAGCAGAGGAGTGGATATCACGGACTGACTCTGACATCATAGATGCAACAATGAAAGAACTCGAGAA

ACTCTTCCCTGATGAAATCTCAGCTGACCAAAGCAAAGCTAAAATCCTGAAGTACCATGTCGTTAAAACA

CCAAGGTCTGTGTATAAGACCATCCCAAACTGTGAACCATGTCGTCCTCTACAGAGATCACCTATTGAAG

GATTCTACTTAGCTGGAGATTACACAAAACAGAAGTACTTAGCTTCCATGGAAGGCGCGGTTCTCTCTGG

CAAATTCTGCTCACAGTCTATTGTGCAGGATTACGAGCTACTGGCTGCATCTGGATCGAGAAAGTTATCG

GAGGCAACAGTATCATCATCATCATCATGA

>Solanum lycopersicum

ATGCCGCCGTGTCTCTGCCTTCCCGCCACTCTCCCTCACCCTTCCTCAACTCTTTTCTCCACGCGCCGCC

GCTTCCCACTACTAAAACCGCCGTATGCTTCCTCAAATATACCCGTTGGCTCAACTTCTCAGCCTAATAC

CACCGGAGTCATTGTCATCGGCGGCGGTTTAGCTGGCCTAGCAGCTGCCATTCGGCTTCAAGCTGACAAC

ATCCCCTTTCTCCTTCTCGAAGCTTCTGATGCCGTTGGTGGTCGCGTTCGGTCCGATGTAGTTGACGGAT

ATACCCTTGATCGTGGTTTCCAGATTTTCATTACTGGTTATCCTGAAGCCCGAAAAGTCCTTGATTATGA

CTCTTTAGACCTCAGAAAATTCTATTCGGGGGCTCAGGTTTACTATGGTGGTCGTTTCCACACCGTTGCT

GATCCTCTCCGGCATTTCGCAGATGCACTGCAATCTTTAACTAACCCAATTGGTTCAGTTGTGGATAAAT

TACTTATAGGATTGACTAGATTGAAAGTTTTGACGCAAGGTGATGATGAAATATTGAGTGCTGATGAAGA

ACCTACGATGAATTTATTAAAGAAGATCGGTTTTTCTGATGCAATATTGGAAAGGTTCTTTCGACCGTTT

TTTGGTGGAATTTTCTTCGATAGAGAGCTTGAAACAACGTCGCGGCTGTTCAATTTCATCTTCAAATGCC

TAGCTCTTGGTGACAACACACTTCCGGCGAAGGGCATTGCGGCCATTCCGGAACAGTTGGCGGCAAAACT

GCCGTCGAATTCAATATTGTTCAATACACGTGTTGTTTCCGTTGATTCAGGATCGGATTCCAGCACAAAA

ATAAGAGTGACACTACAAAATGGGGAAATGTTGGAAAGTGAATTTGGGGTAATATTGGCAGTTGAAGAGC

CTGAAGCTGTCAAGTTGTTGGCGGGAGAAAAAACCGGTGAGGTTCGGCAACCGGTTCGGAGCACAGTATG

TTTGTATTTTTCAGCTGACCAAGGCAAAGTTCCGGTGCAGGATCCGGTTCTTCTTCTTAACGGATCGGGT

AAAGGTATCGTTAATAACATGTTCTTCGCGACCAATGTGGCTCCCTCTTATGCTCCGGCAGGGAAGGCAC

TGGTTTCTGTCACGCTTGTGGGGCTTTATGGTGATGTGGCAGATGAGGATTTGGTGGATCGGGTCGTGAA

GGAGCTATCGGGTTGGTTTGGGGAGTCGGTAGTTGGGTCATGGGGTTACTTGAGGATGTACAGGATCGGG

TTTGCCCAACCGAACCAATGCCCACCCACTAACTTGAAGAAGAACCCGAAAGTGAAACCGGGCTTGTACA

TTTGTGGAGATTATGTGACTAGCGCTACTTTTGATGGAGCTTTAGTTTCTGGGAAAAAAGCAGCAGAAAC

TTTGTTACAAGATAAAGCTCTGGTTATTGTATAG

>Nicotiana tabacum

ATGCCCCAAATTGGACTTGTTTCTGCCGTTAATTTGAGAGTCCAAGGTAATTCAGCTTATCTTTGGAGCT

CGAGGTCTTCTTTGGGAACTGAAAGTCAAGATGGTCACTTGCAAAGGAATTTGTTATGTTTTGGTAGTAG

CGACTCCATGGGGCATAAGTTAAGGATTCGTACTCCCAGTGCCATGACCAGAAGATTGACAAAGGACTTT

AATCCTTTAAAGGTAGTCTGCATTGATTATCCAAGACCAGAGCTAGACAATACAGTTAACTATTTGGAGG

CGGCGTTATTATCATCATCATTTCGTACTTCCTCACGCCCAACTAAACCATTGGAGATTGTTATTGCTGG

TGCAGGTTTGGGTGGTTTGTCTACAGCAAAATATCTGGCTGATGCTGGTCACAAACCGATATTGCTGGAG

GCAAGAGATGTCCTAGGTGGAAAGGTAGCTGCATGGAAAGATGATGATGGAGATTGGTATGAGACTGGGT

TGCACATATTCTTTGGGGCTTACCCAAATATGCAGAACTTGTTTGGAGAACTAGGGATAAACGATCGGTT

GCAGTGGAAGGAACATTCAATGATATTTGCGATGCCTAACAAGCCAGGGGAGTTCAGCCGCTTTGATTTT

CCTGAAGCTCTTCCTGCGCCATTAAATGGAATTTTGGCCATACTAAAGAACAACGAAATGCTTACGTGGC

CCGAAAAAGTCAAATTTGCTATTGGACTCTTGCCAGCAATGCTTGGAGGGCAATCTTATGTTGAAGCTCA

AGACGGTTTAAGTGTTAAGGACTGGATGAGAAAGCAAGGTGTGCCTGATAGGGTGACAGATGAGGTGTTC

ATTGCCATGTCAAAGGCACTTAACTTCATAAACCCTGACGAGCTTTCGATGCAGTGCATTTTGATTGCTT

TGAACAGATTTCTTCAGGAGAAACATGGTTCAAAAATGGCCTTTTTAGATGGTAACCCTCCTGAGAGACT

TTGCATGCCGATTGTTGAACATATTGAGTCAAAAGGTGGCCAAGTCAGACTAAACTCACGAATAAAAAAG

ATTGAGCTGAATGAGGATGGAAGTGTCAAATGTTTTATACTGAATAATGGCAGTACAATTAAAGGAGATG

CTTTTGTGTTTGCCACTCCAGTGGATATCTTCAAGCTTCTTTTGCCTGAAGAGTGGAAAGAGATCCCATA

TTTCCAAAAGTTGGAGAAGCTAGTGGGAGTTCCTGTGATAAATGTCCATATATGGTTTGACAGAAAACTG

AAGAACACATCTGATAATCTGCTCTTCAGCAGAAGCCCATTGCTCAGTGTGTATGCTGACATGTCTGTTA

CATGTAAGGAATATTACAACCCCAATCAGTCTATGTTGGAATTGGTATTTGCACCTGCAGAAGAGTGGAT

AAATCGTAGTGACTCAGAAATTATTGATGCTACAATGAAGGAACTAGCAAAGCTTTTCCCTGACGAAATT

TCGGCAGATCAGAGCAAAGCAAAAATATTGAAGTATCACATTGTCAAAACTCCAAGGTCTGTTTATAAAA

CTGTGCCAGGTTGTGAACCCTGTCGGCCCTTGCAAAGATCTCCTATTGAGGGGTTTTATTTAGCTGGTGA

CTACACAAAACAGAAATACTTGGCTTCAATGGAAGGTGCTGTCTTATCAGGAAAGCTTTGTGCCCAAGCT

ATTGTACAGGATTACGAGTTACTTCTTGGCCGGAGCCAGAAGAAGTTGGCAGAAGCAAGCGTAGTTTAG
