# Supplementary figures and images for: First Report of CRISPR/Cas9 Gene Editing in Castanea sativa Mill
Source: Front Plant Sci. 2021 Aug 25;12:728516. doi: 10.3389/fpls.2021.728516 (PMC8424114; doi:10.3389/fpls.2021.728516)

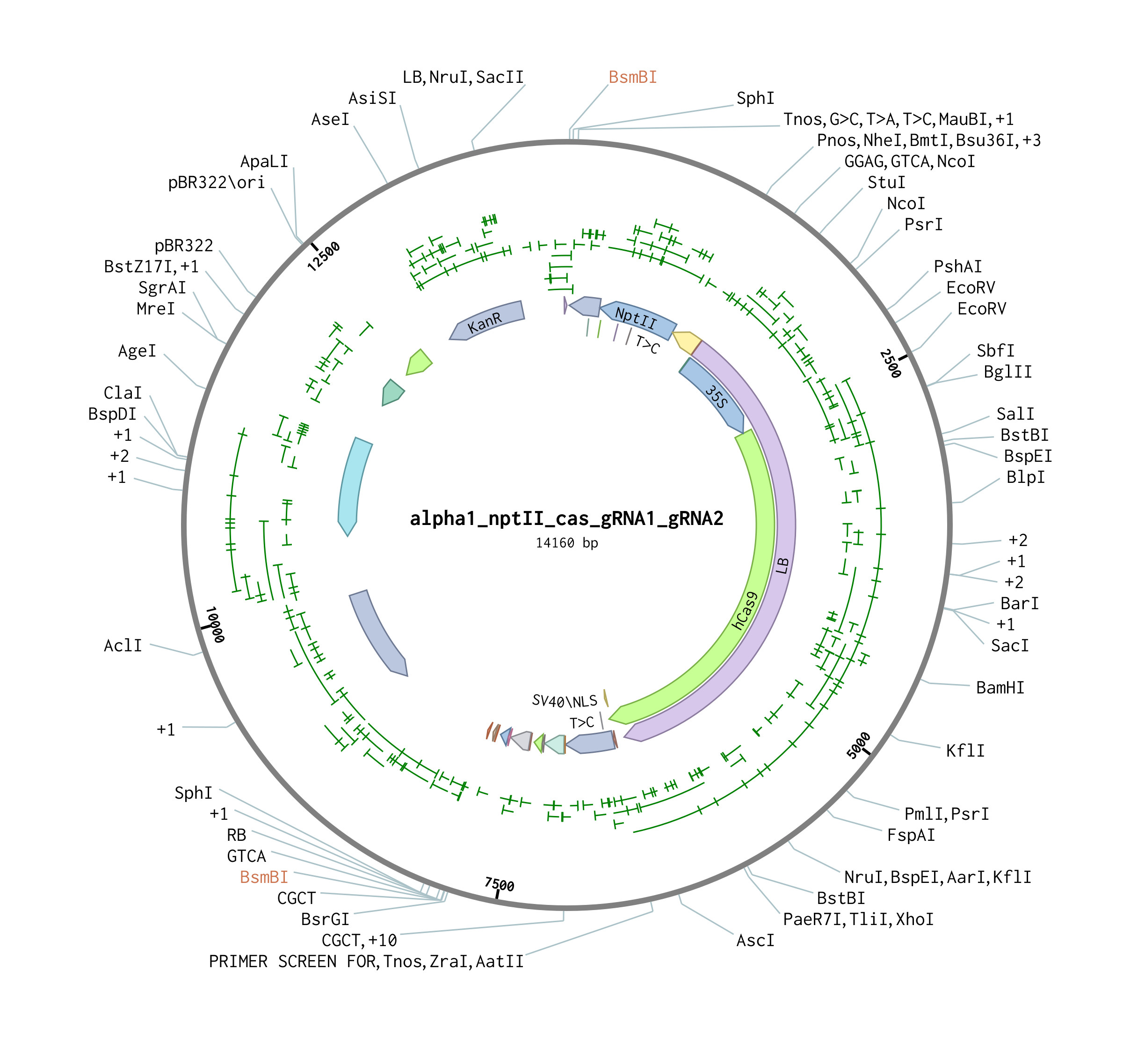

Supplement: Supplementary File 1 — Selected gRNA sequences predicted from C. sativa pds (Correspond to Data sheet 1). [file Data_Sheet_1.zip › Supplemetary File 2.JPEG]

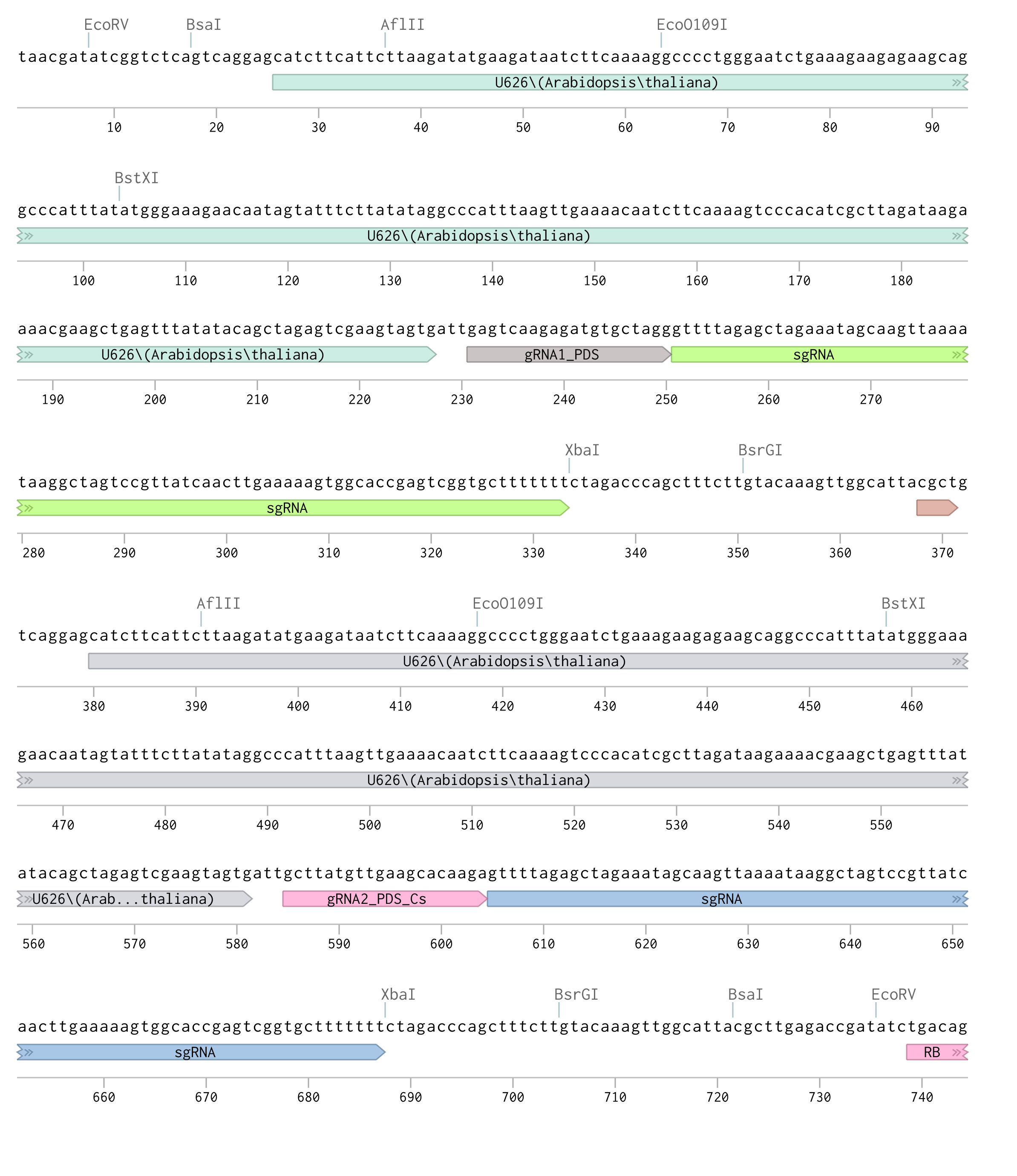

Supplement: Supplementary File 1 — Selected gRNA sequences predicted from C. sativa pds (Correspond to Data sheet 1). [file Data_Sheet_1.zip › Supplemetary File 5.JPEG]

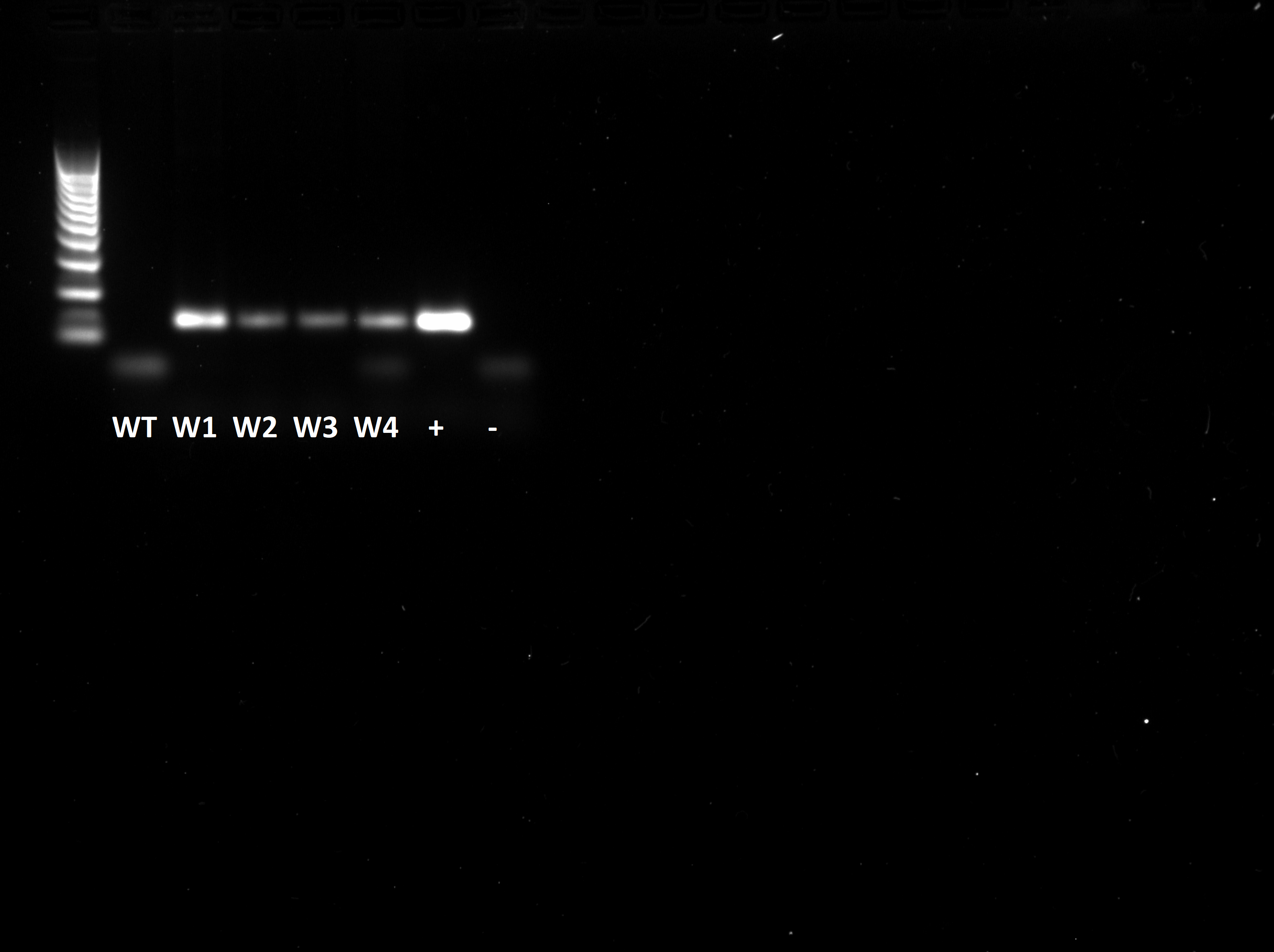

Supplement: Supplementary File 1 — Selected gRNA sequences predicted from C. sativa pds (Correspond to Data sheet 1). [file Data_Sheet_1.zip › Supplemetary File 6.TIF]
